# Supplementary material for: Glucocorticoid Receptor Activation Reprograms NK Cells to Drive AREG‐Mediated Immunosuppression: A Pan‐Cancer Role for AREG
Source: Adv Sci (Weinh). 2025 Oct 13;13(2):e12620. doi: 10.1002/advs.202512620 (PMC12786305; doi:10.1002/advs.202512620)
Supplement: Supplementary file 1 — Supporting Information [file ADVS-13-e12620-s001.pdf]

Supporting information

Glucocorticoid Receptor Activation Reprograms NK Cells to Drive AREG-Mediated Immunosuppression: A Pan-Cancer Role for AREG

Qin Wei, Guirong Liang, Rui zeng, Yuancheng Li, Anlan Hong, Hongsheng Wang, Suying Feng, Yan Wang, Yetao Wang**

Supplementary Figures

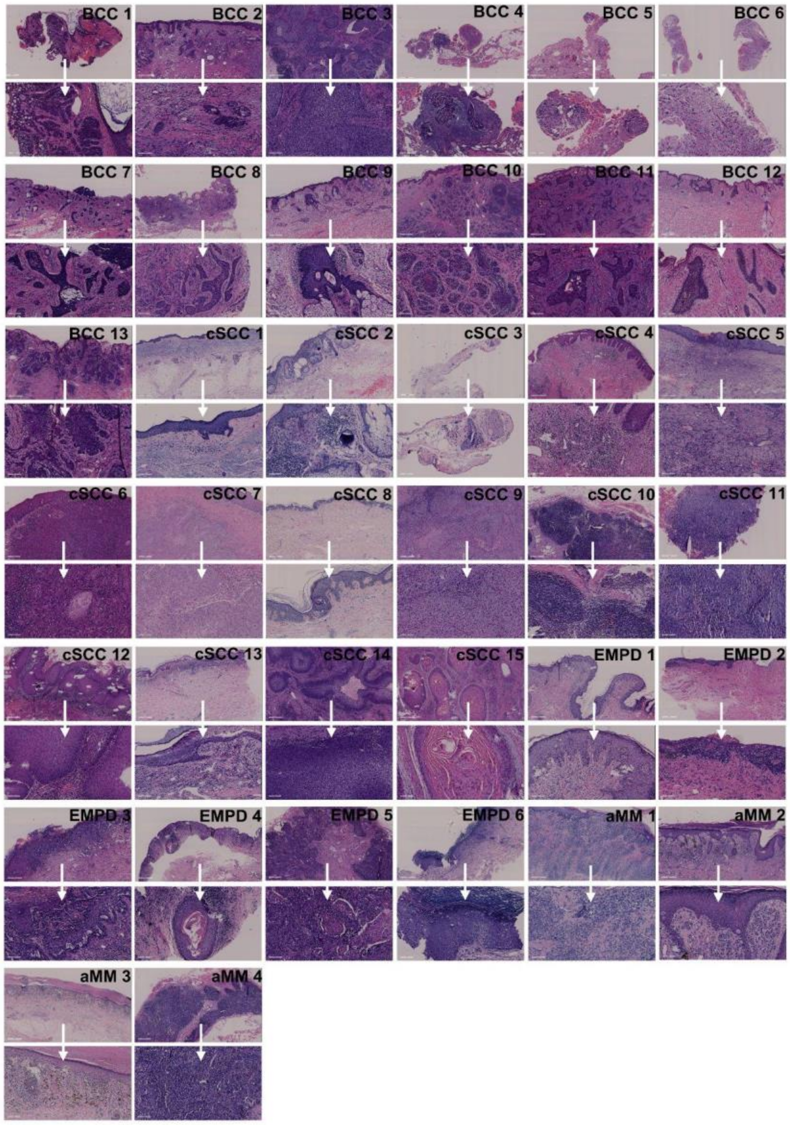

Figure S1. Histological pictures of skin cancer donors. Histological pictures of skin cancer donors for BCC (n=13), cSCC (n=15), EMPD (n=6) and aMM (n=4).

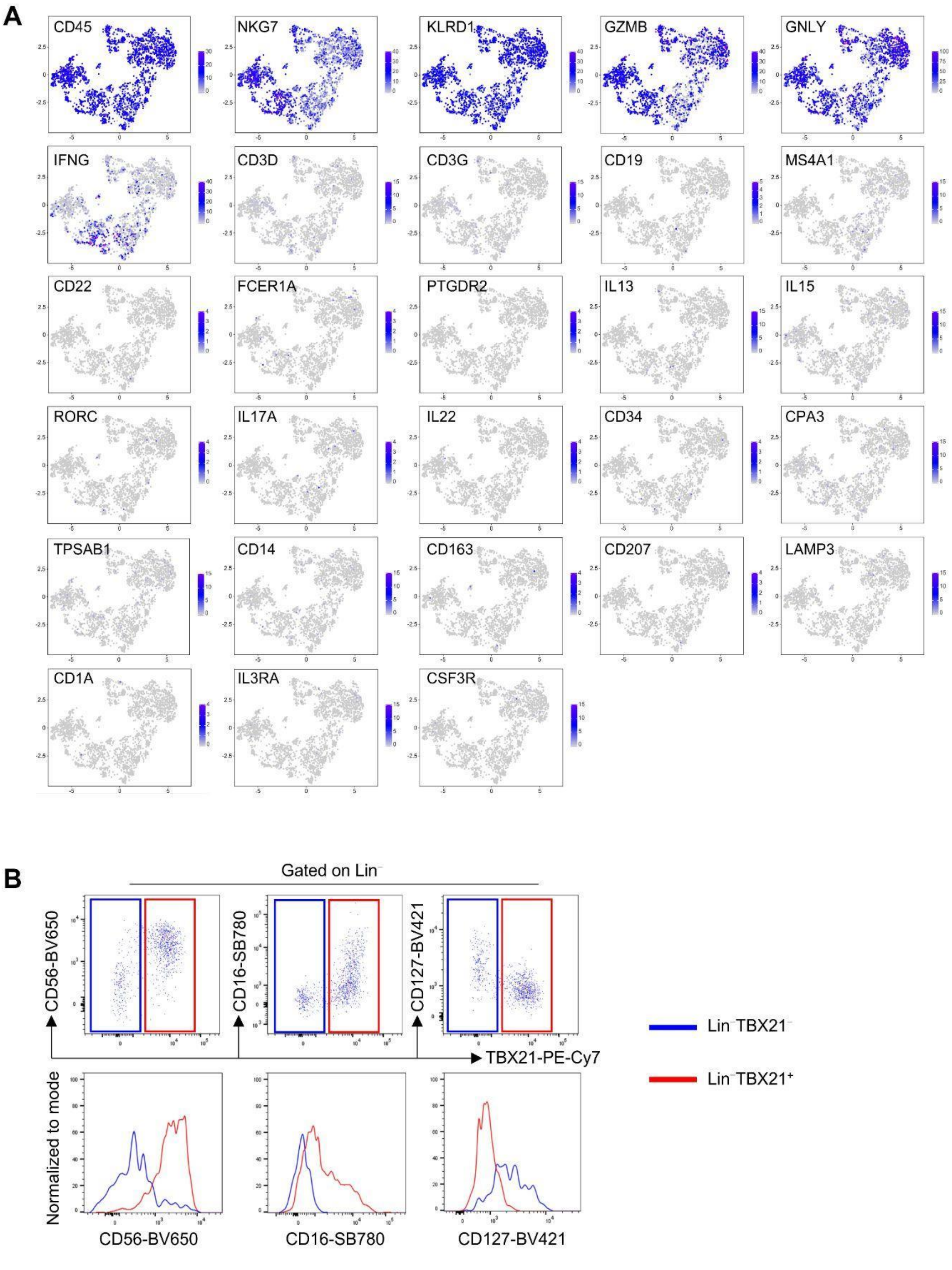

Figure S2. Detection of NK cells by scRNA-Seq. (A) The expression of markers for NK and other cell types in NK cells clusters. (B) CD56, CD16, and CD127 were detected in the Lin^{-}TBX21^{+} or Lin^{-}TBX21^{-} population in the BCC dermis by flow cytometry.

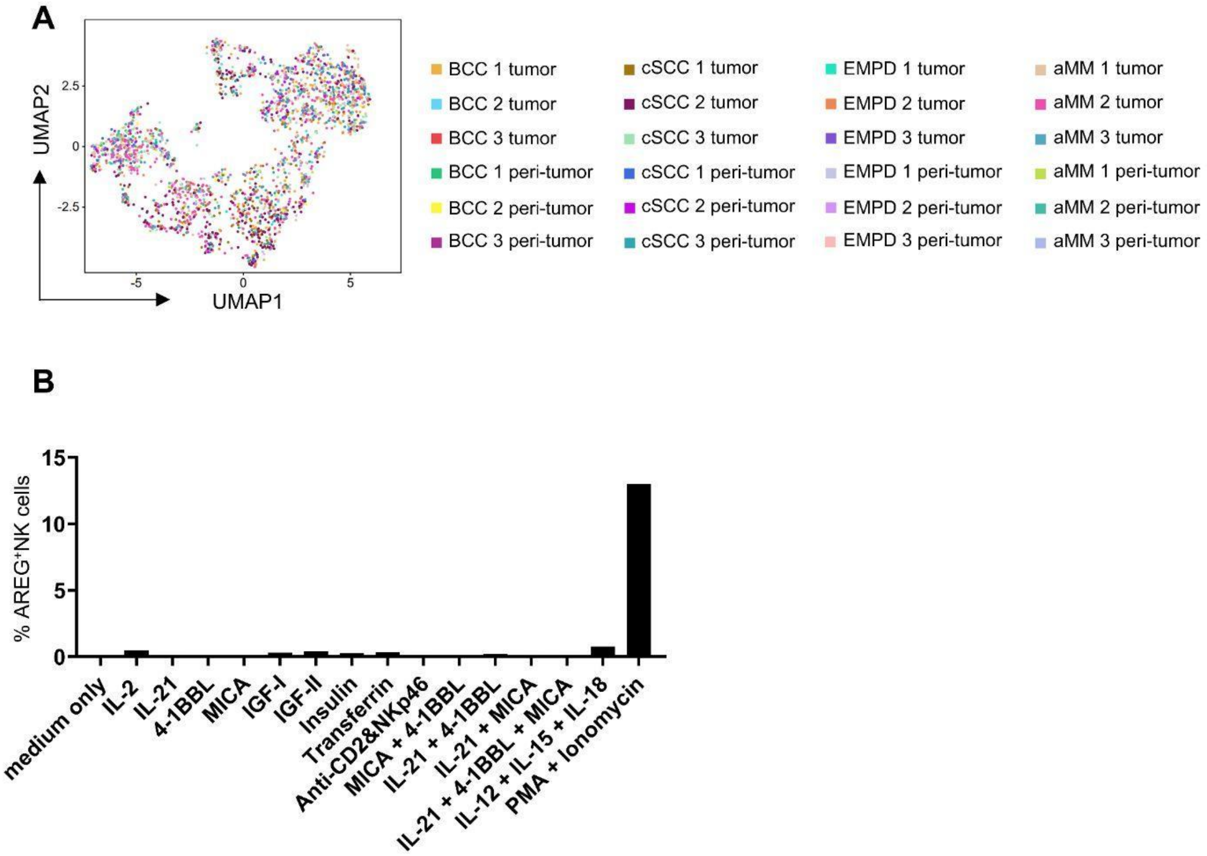

Figure S3. Cluster unique DEgenes of NK cells in skin cancers. (A) Distribution of NK cells in skin tumors and peri-tumor regions across donors. (B) PBMCs were stimulated in indicated conditions for 16 hrs, the AREG production from NK cells (Lin^{-}TBX21^{+}) were detected using flow cytometry.

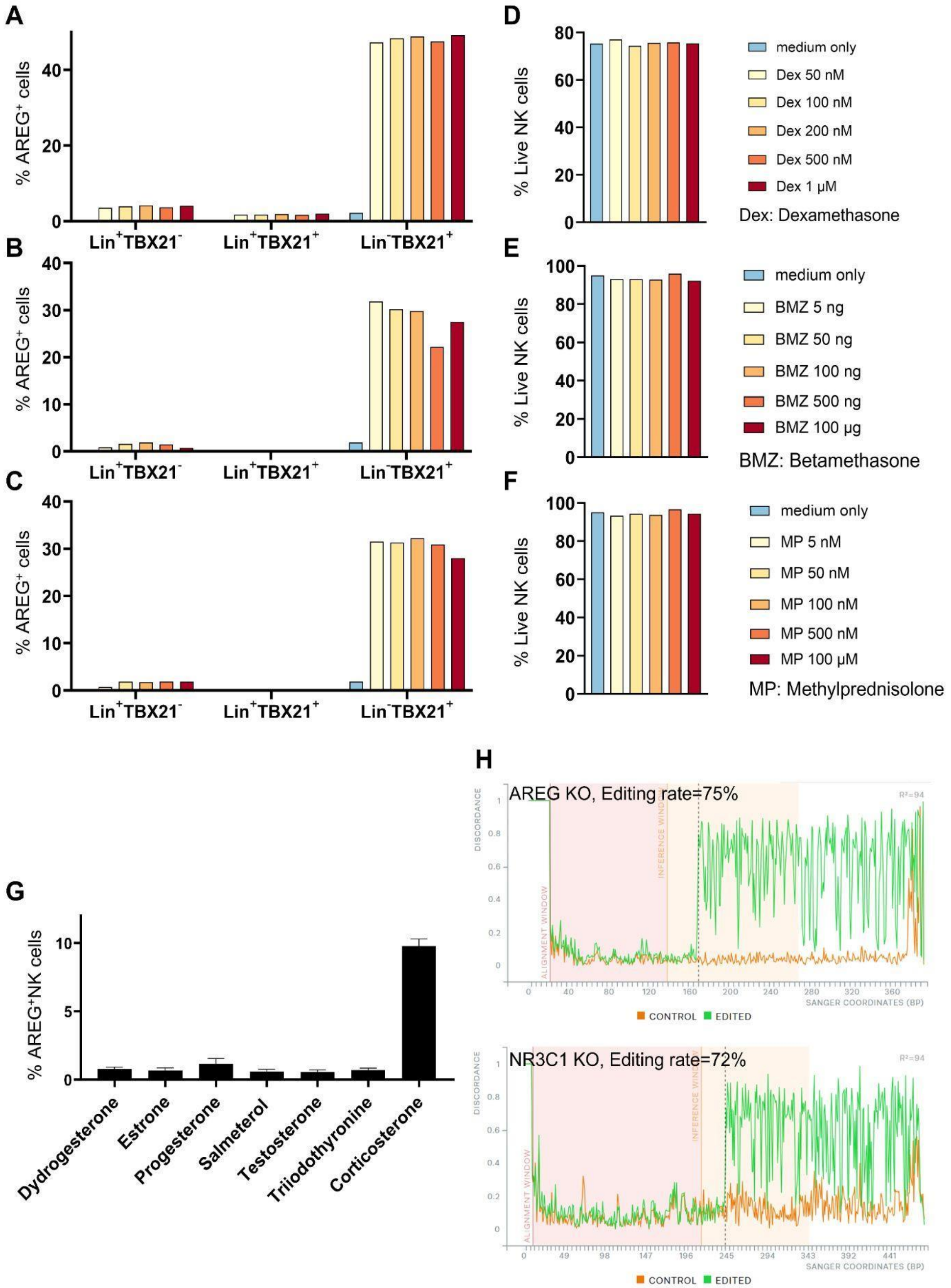

Figure S4. Glucocorticoid stimulation induces NK cell AREG production. (A-C) PBMCs were stimulated in different doses of dexamethasone (A), betamethasone (B) and methylprednisolone (C) for 16 hrs, NK cells produced AREG were detected using flow cytometry. (D-F) Proportion of live NK cells corresponding to (A–C). (G) PBMCs were treated with indicated hormones or hormone analogs for 16 hrs, NK cells produced AREG were detected by flow cytometry. (H) ICE analysis for knockout effect of AREG and NR3C1 in NK cells.

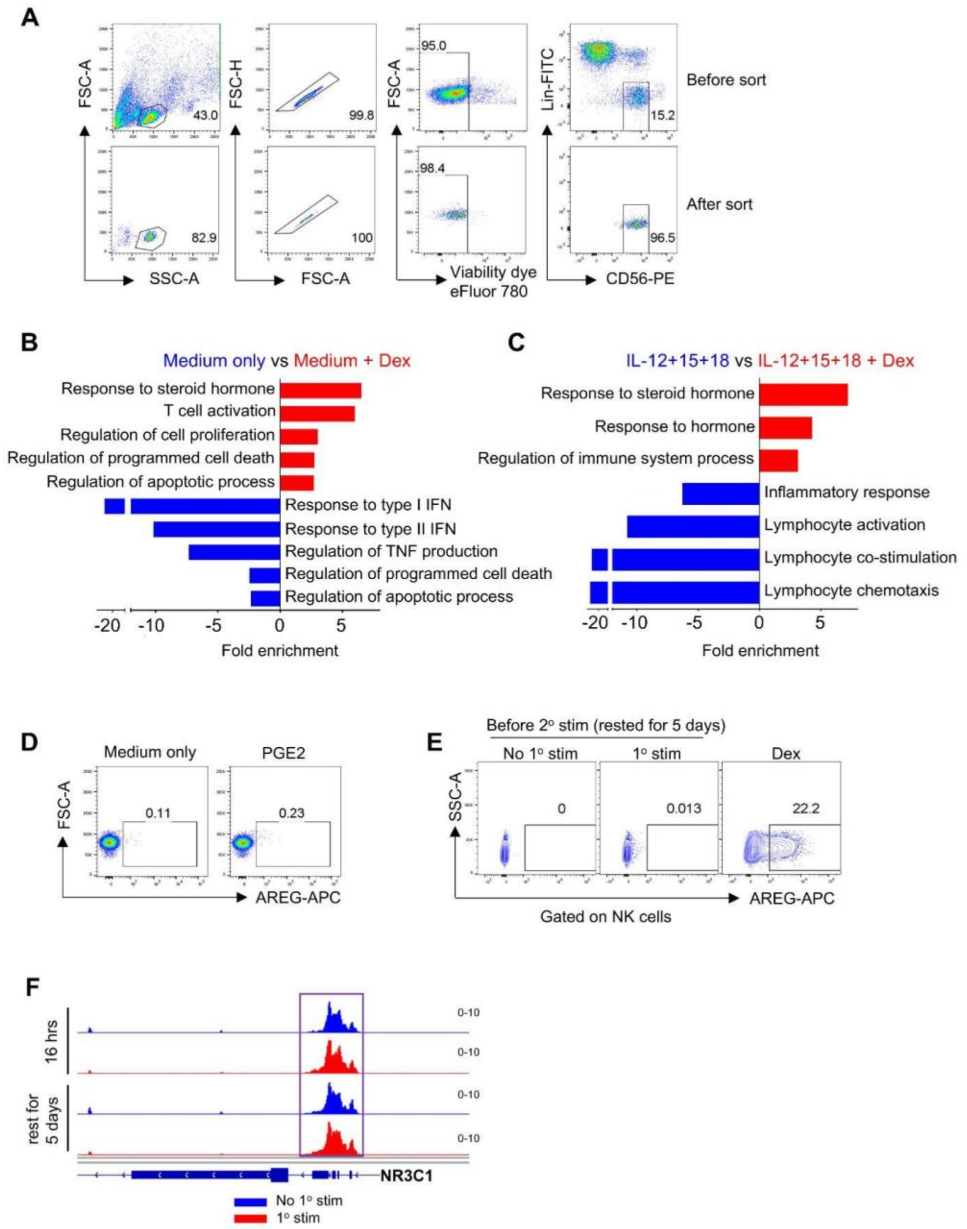

Figure S5. GR activation induces transcriptional alteration in NK cells. (A) Flow cytometry analysis of PBMC-derived NK cells before and after sorting. (B) Enriched pathways of Dex up (red) and down (blue) regulated genes by GO enrichment analysis. (C) Enriched pathways of Dex up (red) and down (blue) regulated genes in the condition of IL-12+IL-15+IL-18 stimulation by GO enrichment analysis. (D) PMBCs were treated with or without PGE2 for 16 hrs, AREG in the NK cell was detected by flow cytometry. (E) PBMCs with or without primary Dex stimulation were cultured in low dose IL-15 for 5 days, before secondary Dex stimulation, the production of AREG by NK cells were detected by flow cytometry. (F) ATAC-Seq analysis of NR3C1 loci of sorted blood NK cells with or without primary Dex stimulation for 16 hrs, and after rest for 5 days.

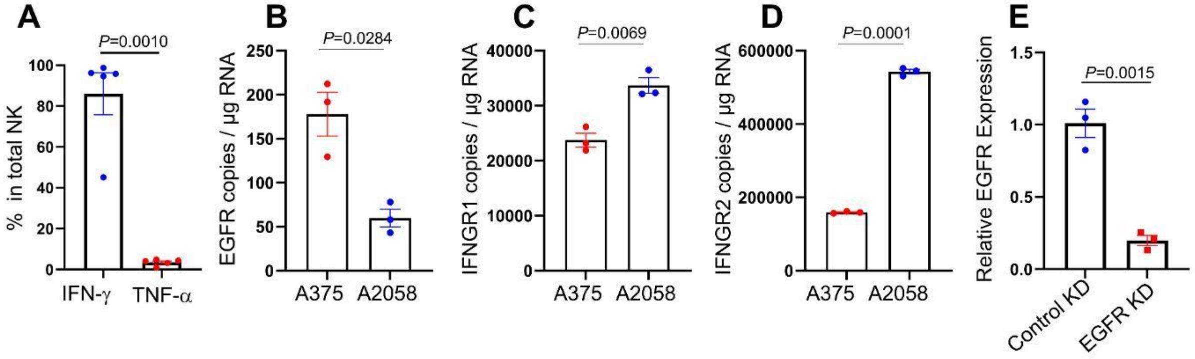

Figure S6. NK cell–induced apoptosis is primarily mediated by IFN-γ. (A) PBMCs were stimulated with IL-12, IL-15, and IL-18 for 16 h, and IFN-γ^+ (n = 5) and TNF-α^+ (n = 5) NK cells were quantified by flow cytometry. (B–D) Expression levels of EGFR, IFNGR1, and IFNGR2 in A375 and A2058 cells (n=3). (E) Efficiency of EGFR knockdown (n=3). For (A), paired t-test; for (B-E), unpaired t-test. Data are mean ± s.e.m.

Table S1 | Skin cancer samples for scRNA-Seq and flow cytometry					
**Sample ID#**	**Sex**	**Age**	**Primary site**	**Experiment**	**Related Figure**
BCC 1	Male	41	Face	scRNA-Seq, flow cytometry	Figure 1, 2, 3, 4, 5
BCC 2	Male	71	Retroauricular region	scRNA-Seq, flow cytometry	Figure 1, 2, 3, 4, 5
BCC 3	Female	81	Upper lip	scRNA-Seq, flow cytometry	Figure 1, 2, 3, 4, 5
BCC 4	Female	82	Ala of nose	flow cytometry	Figure 1, 4
BCC 5	Male	82	Root of nose	flow cytometry	Figure 1, 4
BCC 6	Female	83	Face	flow cytometry	Figure 1, 4
BCC 7	Female	60	Face	flow cytometry	Figure 1, 4
BCC 8	Female	76	Face	flow cytometry	Figure 1, 4
BCC 9	Male	55	Root of nose	flow cytometry	Figure 1, 4
BCC 10	Male	69	Posterior neck region	flow cytometry	Figure 1, 4
BCC 11	Female	69	Temporal region	flow cytometry	Figure 1, 4
BCC 12	Male	59	Axilla	flow cytometry	Figure 1, 4
BCC 13	Male	68	Lip	flow cytometry	Figure 1, 4
cSCC 1	Male	86	Vertex of head	scRNA-Seq, flow cytometry	Figure 1, 2, 3, 4, 5
cSCC 2	Male	86	Craniofacial region	scRNA-Seq, flow cytometry	Figure 1, 2, 3, 4, 5
cSCC 3	Female	87	Temporal region	scRNA-Seq, flow cytometry	Figure 1, 2, 3, 4, 5
cSCC 4	Female	51	Occipital region	flow cytometry	Figure 1, 4
cSCC 5	Male	51	Penis	flow cytometry	Figure 1, 4
cSCC 6	Male	85	Lower lip	flow cytometry	Figure 1, 4
cSCC 7	Male	73	Penis	flow cytometry	Figure 1, 4
cSCC 8	Male	71	Auricle	flow cytometry	Figure 1, 4
cSCC 9	Male	71	Face	flow cytometry	Figure 1, 4
cSCC 10	Female	66	Vulva	flow cytometry	Figure 1, 4
cSCC 11	Female	83	Nose	flow cytometry	Figure 1, 4
cSCC 12	Female	86	Upper eyelid	flow cytometry	Figure 1, 4
cSCC 13	Female	67	Upper arm	flow cytometry	Figure 1, 4
cSCC 14	Male	87	Vertex of head	flow cytometry	Figure 1, 4
cSCC 15	Male	89	Temporal region	flow cytometry	Figure 1, 4
EMPD 1	Male	73	Scrotum	scRNA-Seq , flow cytometry	Figure 1, 2, 3, 4, 5
EMPD 2	Female	42	Labium majus	scRNA-Seq	Figure 1, 2, 3, 5
EMPD 3	Male	70	Penis	scRNA-Seq	Figure 1, 2, 3, 5
EMPD 4	Female	48	Labium majus	flow cytometry	Figure 1, 4
EMPD 5	Male	66	Penis	flow cytometry	Figure 1, 4
EMPD 6	Female	53	Axilla	flow cytometry	Figure 1, 4
aMM 1	Female	68	Plantar region	scRNA-Seq, flow cytometry	Figure 1, 2, 3, 4, 5
aMM 2	Male	81	Hallux	scRNA-Seq	Figure 1, 2, 3, 5
aMM 3	Female	74	Toe	scRNA-Seq	Figure 1, 2, 3, 5
aMM 4	Male	50	Plantar region	flow cytometry	Figure 1, 4

Table S2 | Quality control for scRNA-Seq			
**Sample**	**Estimated Number of Cells**	**Fraction Reads in Cells**	**Mean Reads per Cell**
BCC1_Tumor	6,048	83.10%	77,529
BCC2_Tumor	7,561	91.30%	48,144
BCC3_Tumor	14,042	89.20%	32,057
BCC1_Peri-tumor	7,778	89.50%	57,147
BCC2_Peri-tumor	6,793	93.00%	53,915
BCC3_Peri-tumor	9,240	83.90%	56,502
Empd1_Tumor	9,510	92.90%	37,954
Empd2_Tumor	9,234	90.90%	47,750
Empd3_Tumor	6,338	80.50%	58,405
Empd1_Peri-tumor	10,055	95.50%	43,816
Empd2_Peri-tumor	6,421	93.90%	72,505
Empd3_Peri-tumor	10,311	84.90%	39,961
aMM1_Tumor	10,538	93.60%	38,782
aMM2_Tumor	10,100	94.20%	36,491
aMM3_Tumor	8,237	86.30%	61,182
aMM1_Peri-tumor	14,367	84.50%	26,931
aMM2_Peri-tumor	10,906	92.10%	42,746
aMM3_Peri-tumor	11,242	87.20%	37,815
cSCC1_Tumor	7,542	90.20%	56,883
cSCC2_Tumor	7,163	85.10%	51,865
cSCC3_Tumor	8,007	90.30%	46,905
cSCC1_Peri-tumor	10,643	92.40%	38,657
cSCC2_Peri-tumor	7,472	85.40%	57,480
cSCC3_Peri-tumor	7,489	88.80%	54,668

**Sample**	**Median Genes per Cell**	**Total Genes Detected**	**Median UMI Counts per Cell**
BCC1_Tumor	1,057	24,822	2,643
BCC2_Tumor	1,364	24,078	3,781
BCC3_Tumor	1,098	24,904	2,996
BCC1_Peri-tumor	1,572	28,182	4,338
BCC2_Peri-tumor	1,266	22,840	3,841
BCC3_Peri-tumor	1,389	24,876	3,447
Empd1_Tumor	1,123	26,550	2,855
Empd2_Tumor	1,254	25,411	3,241
Empd3_Tumor	1,227	26,045	3,159
Empd1_Peri-tumor	1,612	27,531	4,546
Empd2_Peri-tumor	1,176	25,222	2,887
Empd3_Peri-tumor	1,093	27,491	2,613
aMM1_Tumor	1,303	25,560	3,623
aMM2_Tumor	1,288	25,834	3,963
aMM3_Tumor	1,122	27,273	3,811
aMM1_Peri-tumor	1,120	27,043	2,407
aMM2_Peri-tumor	1,370	28,223	3,882
aMM3_Peri-tumor	1,072	29,071	2,882
cSCC1_Tumor	1,085	23,422	3,109
cSCC2_Tumor	1,032	24,861	2,316
cSCC3_Tumor	1,526	24,816	4,647
cSCC1_Peri-tumor	1,250	27,538	3,551
cSCC2_Peri-tumor	1,056	24,628	2,979

cSCC3_Peri-tumor	1,372	25,074	4,437

Sample	Reads Mapped Confidently to Genome	Reads Mapped Confidently to Intergenic Regions	Reads Mapped Confidently to Intronic Regions
BCC1_Tumor	91.40%	4.50%	47.50%
BCC2_Tumor	90.90%	6.10%	16.70%
BCC3_Tumor	92.10%	5.50%	23.20%
BCC1_Peri-tumor	90.40%	6.10%	40.30%
BCC2_Peri-tumor	91.70%	6.80%	16.00%
BCC3_Peri-tumor	91.10%	5.50%	17.60%
Empd1_Tumor	91.80%	5.20%	22.00%
Empd2_Tumor	91.90%	4.30%	23.10%
Empd3_Tumor	90.90%	5.30%	32.70%
Empd1_Peri-tumor	92.00%	4.00%	18.20%
Empd2_Peri-tumor	92.60%	5.00%	20.80%
Empd3_Peri-tumor	92.60%	4.50%	31.10%
aMM1_Tumor	93.30%	3.90%	24.40%
aMM2_Tumor	90.00%	5.20%	28.00%
aMM3_Tumor	92.70%	4.70%	36.60%
aMM1_Peri-tumor	90.50%	4.30%	15.80%
aMM2_Peri-tumor	88.90%	5.60%	21.40%
aMM3_Peri-tumor	88.30%	6.80%	40.00%
cSCC1_Tumor	91.80%	6.00%	23.80%
cSCC2_Tumor	90.80%	6.60%	25.80%
cSCC3_Tumor	93.70%	3.30%	32.80%
cSCC1_Peri-tumor	93.10%	5.30%	14.80%
cSCC2_Peri-tumor	92.80%	5.00%	17.20%
cSCC3_Peri-tumor	93.10%	3.80%	34.30%

Sample	Reads Mapped Confidently to Exonic Regions	Reads Mapped Confidently to Transcriptome
BCC1_Tumor	39.40%	33.40%
BCC2_Tumor	68.10%	63.00%
BCC3_Tumor	63.40%	58.30%
BCC1_Peri-tumor	44.10%	37.60%
BCC2_Peri-tumor	68.90%	63.80%
BCC3_Peri-tumor	68.00%	62.60%
Empd1_Tumor	64.70%	58.70%
Empd2_Tumor	64.50%	58.90%
Empd3_Tumor	52.90%	47.70%
Empd1_Peri-tumor	69.80%	63.70%
Empd2_Peri-tumor	66.70%	61.20%
Empd3_Peri-tumor	57.00%	50.40%
aMM1_Tumor	65.00%	59.00%
aMM2_Tumor	56.70%	50.50%
aMM3_Tumor	51.50%	45.30%
aMM1_Peri-tumor	70.40%	64.40%
aMM2_Peri-tumor	61.90%	55.90%
aMM3_Peri-tumor	41.50%	36.00%
cSCC1_Tumor	61.90%	56.00%
cSCC2_Tumor	58.40%	52.70%

cSCC3_Tumor	57.60%	52.20%
cSCC1_Peri-tumor	73.10%	67.00%
cSCC2_Peri-tumor	70.60%	65.30%
cSCC3_Peri-tumor	55.00%	49.40%

Table S3 | Skin tumor NK cells highly expressed genes								
GeneName	Peri-tumor	Tumor	cells.1	cells.2	pct.1	pct.2	log2FC	p_value
GZMA	2.37394802	6.88231292	549	1733	0.335	0.49	1.53560514	3.33E-14
DUSP1	3.8074024	10.2655046	549	1733	0.485	0.66	1.43092559	4.23E-18
GZMK	1.01937565	2.61254397	549	1733	0.193	0.331	1.35776953	6.13E-12
CCL3	3.83741468	8.89263658	549	1733	0.259	0.373	1.21247655	1.16E-07
CCL4L2	3.5935138	8.02218912	549	1733	0.188	0.29	1.15860075	1.80E-06
NR4A1	1.96685609	4.2034709	549	1733	0.268	0.398	1.09568969	8.44E-10
DNAJB1	13.7464422	28.3640824	549	1733	0.514	0.698	1.04500692	2.52E-16
RGS1	1.82462571	3.52305124	549	1733	0.237	0.403	0.94922491	2.40E-13
CCL4	13.5545009	26.1518622	549	1733	0.464	0.579	0.94814169	5.91E-07
IFNG	4.18527632	7.96759147	549	1733	0.293	0.413	0.9288208	1.81E-06
MIR23AHG	1.99385372	3.76334104	549	1733	0.361	0.443	0.91645446	1.52E-05
EMB	0.53596628	1.00315018	549	1733	0.164	0.293	0.90432347	3.40E-09
CTSD	0.71422889	1.33224909	549	1733	0.209	0.327	0.89940546	6.08E-08
AREG	3.03869477	5.64697285	549	1733	0.313	0.419	0.89402593	2.31E-07
EVL	0.88822243	1.64175028	549	1733	0.22	0.367	0.88624179	3.53E-10
PPP1R15A	5.50945361	9.38606215	549	1733	0.579	0.697	0.76861076	5.25E-08
DNAJB4	0.80546541	1.3458864	549	1733	0.175	0.253	0.74066212	4.91E-04
DDX3Y	1.68447835	2.81270006	549	1733	0.259	0.463	0.73965383	2.55E-17
ACTB	7.60056178	12.5133739	549	1733	0.78	0.88	0.71929287	7.85E-13
ERN1	1.07070921	1.74744579	549	1733	0.226	0.321	0.70668098	6.47E-05
TSC22D3	4.89605992	7.98639562	549	1733	0.523	0.719	0.70592332	5.66E-18
SH3BGRL	0.5831246	0.94333689	549	1733	0.166	0.279	0.69396889	1.82E-07
RNF213	2.30794093	3.69097808	549	1733	0.45	0.589	0.67739687	4.49E-09
LY6E	0.7961081	1.26524577	549	1733	0.222	0.329	0.66838141	7.02E-06
JUN	6.18872063	9.78707524	549	1733	0.448	0.588	0.66123659	5.96E-09
COTL1	1.62636851	2.57133882	549	1733	0.299	0.441	0.66086553	1.07E-08
ALOX5AP	1.04885935	1.65103454	549	1733	0.208	0.319	0.65454908	1.63E-06
HAVCR2	1.02130445	1.6023851	549	1733	0.211	0.342	0.64980792	2.58E-08
BCAS2	1.07468549	1.67998833	549	1733	0.279	0.37	0.6445367	2.80E-04
FMNL1-DT	0.91720939	1.39065038	549	1733	0.239	0.351	0.60043674	3.35E-06
HLA-F	1.49440673	2.26191423	549	1733	0.352	0.509	0.59797137	6.71E-10
GBP5	1.03536311	1.55028312	549	1733	0.222	0.313	0.58239489	1.36E-04
DDX27	0.68677486	1.01293698	549	1733	0.195	0.282	0.56063528	2.03E-04
KLF6	6.35710117	9.34269225	549	1733	0.689	0.761	0.5554693	5.62E-08
FOS	8.23548385	12.0921447	549	1733	0.424	0.569	0.55414483	3.35E-09
NFKBIA	11.1144311	16.2850364	549	1733	0.778	0.842	0.55111284	2.18E-08
USP16	0.61090606	0.89489702	549	1733	0.175	0.263	0.55077113	1.00E-04
NFKBID	0.98713586	1.44179499	549	1733	0.244	0.301	0.54654549	0.00472093
IFITM1	3.10930492	4.45595974	549	1733	0.534	0.666	0.5191441	2.02E-08
CALM3	0.68578978	0.98242984	549	1733	0.209	0.285	0.51858797	0.00169549
MATK	1.36638421	1.95037941	549	1733	0.341	0.462	0.51339159	2.27E-06
ISG20	3.11961645	4.44981327	549	1733	0.505	0.604	0.51237613	1.26E-05
KLHL6	0.75105806	1.06962538	549	1733	0.186	0.254	0.51010926	0.00328905
CEBPD	1.44636305	2.0502869	549	1733	0.255	0.312	0.50339608	0.01400605
IL4R	0.58591764	0.82843268	549	1733	0.166	0.253	0.49968659	3.14E-05
HCST	4.21131853	5.91227775	549	1733	0.627	0.754	0.48944205	1.26E-10
VPS28	0.61534058	0.86328243	549	1733	0.173	0.256	0.48844748	1.81E-04
PLP2	0.85956896	1.20119762	549	1733	0.246	0.336	0.48278822	3.11E-04
LAT2	0.92003739	1.28505909	549	1733	0.237	0.327	0.48207031	2.74E-04
GZMB	10.8556317	15.1440735	549	1733	0.628	0.705	0.48030964	0.00373448
TXK	0.84817716	1.17967304	549	1733	0.208	0.278	0.47594952	0.00356026
PARP8	1.18306293	1.64263195	549	1733	0.304	0.402	0.47348245	1.60E-04
BIN2	0.93188136	1.29170766	549	1733	0.233	0.338	0.47106141	1.18E-05
SYAP1	2.13292361	2.95164808	549	1733	0.426	0.526	0.46868843	2.87E-05
CD69	9.38268978	12.9804097	549	1733	0.701	0.818	0.46826245	7.33E-09
HSPA1A	63.2868414	87.4251219	549	1733	0.763	0.85	0.46614234	1.03E-07
CARD16	0.81044413	1.116349	549	1733	0.193	0.278	0.46200348	2.78E-04

TYROBP	4.29654766	5.9070877	549	1733	0.628	0.784	0.45926914	8.56E-13
BBLN	0.98250373	1.34990977	549	1733	0.279	0.368	0.45832818	5.35E-04
HSP90AA1	101.880215	139.762354	549	1733	0.913	0.977	0.4561019	7.58E-17
FCER1G	2.58251206	3.52933141	549	1733	0.399	0.548	0.45061983	7.76E-09
IRF8	1.38398139	1.8856344	549	1733	0.253	0.373	0.44622544	5.55E-07
KDM5B	1.14582969	1.5592968	549	1733	0.288	0.362	0.44450293	0.00361016
FOXN3	0.6008135	0.81695791	549	1733	0.175	0.253	0.44334452	3.97E-04
NOP58	1.16878634	1.58709227	549	1733	0.301	0.394	0.44137478	3.33E-04
ADGRE5	3.96858555	5.35940923	549	1733	0.648	0.735	0.43344907	2.81E-08
SIAH2	0.64223152	0.86718606	549	1733	0.193	0.253	0.43324809	0.01438455
BST2	1.2105129	1.62109303	549	1733	0.313	0.39	0.42134843	0.0024844
RIN3	0.87679332	1.17276153	549	1733	0.246	0.329	0.41960097	7.68E-04
NSG0000028288	0.80562622	1.07737187	549	1733	0.197	0.299	0.41933377	1.30E-06
NAMPT	3.01505579	4.02803994	549	1733	0.468	0.565	0.41789329	3.12E-04
CORO1A	2.27493389	3.038773	549	1733	0.486	0.593	0.41766429	2.62E-05
ANAPC16	0.88542398	1.18167387	549	1733	0.235	0.317	0.41639156	9.83E-04
STARD3NL	0.69276715	0.92400614	549	1733	0.208	0.278	0.41553192	0.00374946
IFITM3	1.56001422	2.08043497	549	1733	0.319	0.412	0.41532601	4.18E-04
CRYBG1	1.02592335	1.36814847	549	1733	0.25	0.337	0.41530186	3.85E-04
PAXX	1.26820989	1.69079586	549	1733	0.333	0.396	0.41490895	0.01311021
UBC	19.9553414	26.6001303	549	1733	0.909	0.947	0.41465835	7.13E-06
CRIP1	2.71517672	3.61505445	549	1733	0.395	0.555	0.41297128	2.65E-10
ZBTB16	1.13875942	1.51577254	549	1733	0.211	0.295	0.41259029	3.71E-04
NSG0000027225	1.0315028	1.3678913	549	1733	0.22	0.317	0.40720586	5.72E-05
COX20	0.67885821	0.89999305	549	1733	0.208	0.269	0.40680358	0.0131369
MYADM	0.97535994	1.29180191	549	1733	0.224	0.317	0.40537823	7.97E-05
HSPE1	16.7095421	22.1004467	549	1733	0.783	0.869	0.40340333	3.46E-06
HSPA1B	18.4223471	24.3565565	549	1733	0.475	0.594	0.4028533	2.08E-07
RESF1	1.43188377	1.88964449	549	1733	0.344	0.398	0.40020045	0.01947754
RPS4Y1	2.14446295	2.82930824	549	1733	0.319	0.525	0.39983297	3.57E-21
CASP4	0.7147167	0.93910683	549	1733	0.222	0.291	0.39391778	0.00616202
SLA2	0.98985915	1.29620705	549	1733	0.242	0.307	0.38900102	0.00834108
VPS13C	1.0654196	1.39434674	549	1733	0.259	0.345	0.38816764	3.63E-04
FAM177A1	3.78798343	4.95004557	549	1733	0.503	0.63	0.38601179	9.21E-07
LSP1	1.20102258	1.56916636	549	1733	0.286	0.388	0.38573504	5.32E-05
TLN1	1.71558508	2.24070114	549	1733	0.393	0.511	0.38524956	9.52E-06
CTSW	3.34551612	4.36802389	549	1733	0.532	0.653	0.38475195	2.17E-06
PDE4A	1.44154898	1.88204786	549	1733	0.308	0.384	0.38468345	0.00344216
JUNB	8.33735788	10.8800854	549	1733	0.705	0.776	0.38402771	0.00157884
GMFG	1.00156708	1.30644483	549	1733	0.268	0.348	0.38338715	0.00196988
GRK2	1.00985569	1.31713843	549	1733	0.27	0.362	0.38325783	1.53E-04
ATP6V1F	0.71384692	0.93041211	549	1733	0.224	0.297	0.38225514	0.0034022
GZMM	1.85344923	2.41548909	549	1733	0.353	0.481	0.38210274	6.87E-07
IL2RB	2.63601462	3.43064765	549	1733	0.483	0.609	0.38012259	1.31E-06
ANKRD44	0.74785743	0.97085586	549	1733	0.191	0.279	0.37649385	1.93E-05
ARID1B	0.94910964	1.23144813	549	1733	0.246	0.323	0.3757092	0.00253769
APOL6	1.00384767	1.30136036	549	1733	0.231	0.336	0.37448015	3.37E-06
HSPH1	13.3515375	17.2114447	549	1733	0.627	0.752	0.36636232	1.50E-07

Table S4 | Highly expressed genes for each NK cell cluster							
Target Cluster	Gene Name	Target Cluster pct	Other Cluster pct	Target Cluster mean	Other Cluster mean	Log2FC	Pvalue
0	CD44	0.95	0.69	15.97	5.59	1.51	5.86E-161
0	XCL1	0.89	0.40	32.55	11.26	1.53	1.45E-138
0	IL7R	0.70	0.28	15.95	2.82	2.50	2.71E-116
0	GPR183	0.71	0.27	7.54	1.83	2.04	1.12E-111
0	REL	0.94	0.77	25.38	10.33	1.30	9.26E-110
0	TNFRSF18	0.82	0.38	6.92	2.27	1.61	1.76E-109
0	TCF7	0.68	0.25	4.54	1.15	1.98	7.10E-105
0	XCL2	0.92	0.58	40.98	17.18	1.25	2.59E-98
0	NFKB1	0.85	0.51	9.71	3.62	1.42	9.71E-96
0	JAK1	0.93	0.75	12.87	6.04	1.09	2.77E-90
0	BTG1	0.99	0.96	54.60	30.51	0.84	8.07E-86
0	KLRC1	0.75	0.34	8.50	3.18	1.42	1.06E-81
0	TPT1	0.99	0.99	71.66	48.84	0.55	3.94E-80
0	AHI1	0.67	0.34	7.23	1.84	1.97	1.82E-75
0	PABPC1	0.96	0.87	16.69	9.14	0.87	3.49E-72
0	ITM2C	0.51	0.17	2.65	0.70	1.92	1.94E-70
0	SRGN	0.97	0.96	54.53	28.77	0.92	2.42E-69
0	VIM	0.95	0.87	35.28	16.11	1.13	2.31E-68
0	SPTBN1	0.62	0.31	4.15	1.31	1.66	1.98E-66
0	IL2RA	0.29	0.04	1.11	0.12	3.19	6.72E-66
0	PDE4A	0.56	0.24	3.03	0.95	1.68	1.40E-61
0	NFE2L2	0.80	0.53	6.48	3.08	1.07	8.51E-60
0	SNHG16	0.59	0.29	3.36	1.16	1.54	2.58E-58
0	AGO2	0.60	0.29	3.20	1.14	1.49	2.98E-57
0	BACH2	0.32	0.07	1.29	0.21	2.63	1.76E-55
0	STAM	0.50	0.20	2.03	0.72	1.49	6.50E-54
0	TIAM1	0.30	0.07	1.22	0.22	2.45	3.81E-52
0	IFITM3	0.57	0.27	3.17	1.15	1.46	1.64E-50
0	IL12RB2	0.31	0.08	1.28	0.24	2.40	3.16E-50
0	SMAP2	0.81	0.56	6.87	3.62	0.92	5.26E-50
0	CD55	0.69	0.41	4.91	2.21	1.15	5.67E-50
0	FCER1G	0.70	0.39	4.71	2.37	0.99	6.70E-49
0	LMNA	0.79	0.50	8.11	4.82	0.75	4.57E-48
0	CRTAM	0.58	0.30	7.19	2.68	1.43	7.22E-48
0	FES	0.29	0.07	1.24	0.24	2.37	1.82E-47
0	MYC	0.32	0.09	1.76	0.51	1.79	8.66E-47
0	NINJ1	0.56	0.27	2.72	1.12	1.28	3.78E-46
0	SKP1	0.84	0.67	6.99	3.97	0.81	4.97E-46
0	FURIN	0.41	0.15	1.52	0.51	1.58	1.29E-44
0	KDM6B	0.73	0.48	5.72	2.89	0.98	8.19E-42
0	METRNL	0.86	0.64	10.44	6.07	0.78	1.03E-41
0	ECE1	0.32	0.10	1.16	0.29	1.98	1.50E-41
0	FMNL1-DT	0.48	0.22	2.00	0.80	1.33	3.60E-41
0	SELL	0.31	0.09	1.30	0.33	1.96	5.32E-41
0	RPLP1	0.99	0.99	70.09	54.23	0.37	4.09E-40
0	SATB1	0.50	0.25	2.67	0.95	1.50	1.16E-39
0	CXXC5	0.42	0.17	1.64	0.63	1.38	1.65E-39
0	ABHD2	0.48	0.23	2.09	0.82	1.35	2.42E-39
0	FAM177A1	0.73	0.51	6.84	3.24	1.08	3.08E-39
0	BIRC3	0.77	0.55	15.92	7.50	1.09	3.27E-38
0	GOLIM4	0.34	0.12	1.35	0.40	1.75	1.23E-37
0	RPS24	0.99	0.98	40.59	31.47	0.37	1.26E-37
0	PGK1	0.72	0.49	4.88	2.61	0.90	1.86E-37
0	FOSL2	0.76	0.54	5.50	3.02	0.86	2.37E-37

0	ANXA11	0.53	0.28	2.32	0.99	1.22	2.70E-37
0	SERBP1	0.84	0.65	6.33	3.92	0.69	2.12E-36
0	NFKB2	0.59	0.35	2.97	1.45	1.04	2.01E-35
0	RPS5	0.95	0.91	15.15	10.98	0.46	2.57E-35
0	LEPROTL1	0.68	0.45	4.09	2.05	1.00	2.92E-35
0	TMEM123	0.51	0.27	2.37	1.01	1.23	3.43E-35
0	TGFB1	0.84	0.69	8.31	4.91	0.76	4.46E-35
0	RAMP1	0.27	0.08	1.20	0.34	1.81	4.79E-35
0	PDE4B	0.63	0.36	3.40	1.91	0.83	1.17E-34
0	PAK1	0.26	0.08	0.79	0.22	1.86	2.38E-34
0	SPAG1	0.29	0.10	0.97	0.29	1.75	5.08E-34
0	RPL9	0.98	0.96	22.33	17.02	0.39	1.18E-33
0	LPXN	0.51	0.29	2.49	1.04	1.26	3.80E-33
0	TAMALIN	0.44	0.20	2.15	1.00	1.10	4.67E-33
0	WSB1	0.69	0.50	5.62	2.64	1.09	5.98E-33
0	KLHL6	0.37	0.15	1.49	0.66	1.17	6.73E-33
0	ZFPM1	0.29	0.10	1.04	0.30	1.78	7.47E-33
0	IRAG2	0.50	0.26	2.19	1.03	1.09	1.04E-32
0	AREG	0.55	0.29	6.84	3.82	0.84	1.44E-32
0	ERGIC1	0.36	0.15	1.30	0.49	1.40	9.54E-32
0	PPP1CB	0.71	0.49	4.04	2.32	0.80	1.53E-31
0	ATP1B1	0.42	0.19	2.26	0.95	1.25	1.67E-31
0	TAGLN2	0.77	0.61	5.94	3.55	0.74	1.72E-31
0	MRPS6	0.61	0.39	3.90	1.92	1.02	1.93E-31
0	CREM	0.86	0.69	12.29	8.38	0.55	3.10E-31
0	DUSP4	0.47	0.22	2.55	1.44	0.82	2.08E-30
0	CD7	0.84	0.70	8.98	5.65	0.67	5.65E-30
0	RIN3	0.44	0.22	1.65	0.74	1.17	1.63E-29
0	PRKX	0.51	0.29	2.48	1.19	1.06	2.22E-29
0	B4GALT1	0.70	0.48	4.03	2.41	0.74	2.38E-29
0	FGFR1OP2	0.75	0.56	5.15	3.05	0.75	3.03E-29
0	ZEB1	0.28	0.10	1.02	0.31	1.70	5.43E-29
0	RPS2	0.97	0.97	33.35	24.99	0.42	5.79E-29
0	RHBDF2	0.33	0.14	1.22	0.46	1.41	6.05E-29
0	ZBTB16	0.40	0.19	2.23	0.89	1.32	6.31E-29
0	RAB21	0.63	0.42	3.33	1.81	0.88	7.80E-29
0	PTMA	0.99	0.99	57.45	44.70	0.36	2.14E-28
0	SYPL1	0.34	0.15	1.14	0.44	1.37	2.22E-28
0	EML4	0.65	0.42	3.48	2.00	0.80	2.39E-28
0	TRAF1	0.36	0.16	1.47	0.56	1.40	2.50E-28
0	DENND4A	0.47	0.26	2.21	0.94	1.24	2.95E-28
0	SARAF	0.86	0.75	8.59	5.87	0.55	1.21E-27
0	STAG2	0.63	0.42	3.27	1.85	0.82	1.29E-27
0	B3GNT7	0.43	0.22	1.69	0.86	0.97	1.49E-27
0	SLC16A3	0.31	0.12	1.01	0.39	1.37	2.98E-27
0	SEC14L1	0.48	0.27	2.14	0.99	1.11	4.16E-27
0	ZFP36L2	0.90	0.80	15.96	10.27	0.64	9.52E-27
0	MKNK2	0.62	0.41	3.42	1.91	0.84	1.43E-26
0	TNFRSF9	0.44	0.24	4.41	1.72	1.36	2.61E-26
0	GPX4	0.71	0.51	4.21	2.61	0.69	3.03E-26
0	RALGAPA1	0.57	0.37	3.03	1.58	0.94	6.93E-26
0	NFAT5	0.56	0.37	3.62	1.76	1.04	2.38E-25
0	IKZF2	0.29	0.12	1.21	0.47	1.37	2.39E-25
0	RPLP0	0.91	0.84	11.82	8.49	0.48	2.93E-25
0	KDM5B	0.47	0.26	2.04	1.07	0.93	2.99E-25
0	SIPA1L1	0.29	0.12	1.11	0.38	1.56	3.51E-25
0	SNX9	0.26	0.10	0.94	0.32	1.53	6.68E-25
0	SESN1	0.30	0.13	1.23	0.49	1.33	8.65E-25
0	WHRN	0.36	0.18	1.75	0.74	1.24	1.08E-24

0	FYN	0.81	0.65	6.26	4.21	0.57	5.40E-24
0	IFITM2	0.89	0.83	13.37	9.12	0.55	6.39E-24
0	PIK3R1	0.84	0.71	11.88	7.91	0.59	6.41E-24
0	ADGRE5	0.80	0.66	6.34	4.15	0.61	6.44E-24
0	IL2RB	0.68	0.51	4.17	2.63	0.67	6.57E-24
0	RBPJ	0.49	0.29	2.48	1.19	1.06	7.00E-24
0	RERE	0.31	0.14	1.08	0.47	1.21	1.03E-23
0	LRRFIP1	0.78	0.65	7.89	4.90	0.69	1.27E-23
0	CAPG	0.27	0.11	0.97	0.40	1.29	2.46E-23
0	VDAC1	0.48	0.28	1.81	0.96	0.92	2.87E-23
0	EMD	0.50	0.30	1.91	1.06	0.86	3.32E-23
0	SLC38A1	0.75	0.59	5.01	3.32	0.59	3.52E-23
0	SMAD3	0.27	0.11	0.93	0.34	1.47	6.23E-23
0	PSMD13	0.41	0.22	1.56	0.74	1.07	6.94E-23
0	PPP1R14B	0.43	0.23	1.76	0.97	0.86	1.23E-22
0	CTNNB1	0.53	0.33	2.60	1.36	0.94	2.04E-22
0	ZHX2	0.28	0.12	0.96	0.36	1.43	3.39E-22
0	TMSB4X	0.99	0.99	118.30	87.21	0.44	4.49E-22
0	FBXO34	0.43	0.24	1.69	0.84	1.01	4.67E-22
0	BID	0.31	0.14	0.94	0.43	1.11	5.99E-22
0	EZR	0.87	0.74	11.80	8.33	0.50	7.33E-22
0	KLRD1	0.91	0.87	13.01	9.47	0.46	1.34E-21
0	SKIL	0.56	0.36	2.65	1.51	0.82	2.01E-21
0	OGT	0.58	0.38	2.77	1.65	0.75	2.24E-21
0	FOXP1	0.46	0.27	2.37	1.11	1.09	2.49E-21
0	ANKRD11	0.56	0.38	3.25	1.77	0.87	3.09E-21
0	BICDL1	0.40	0.22	1.91	0.89	1.10	3.40E-21
0	CHMP4B	0.30	0.14	0.94	0.40	1.23	5.93E-21
0	IL4R	0.34	0.16	1.11	0.55	1.01	6.06E-21
0	SYTL3	0.70	0.55	5.15	3.24	0.67	8.59E-21
0	CLDND1	0.59	0.40	3.59	2.11	0.77	1.01E-20
0	NCK2	0.30	0.14	0.93	0.42	1.15	1.01E-20
0	GSPT1	0.56	0.36	2.78	1.66	0.74	1.49E-20
0	BRD1	0.46	0.27	1.91	0.99	0.95	1.59E-20
0	BCL2A1	0.49	0.31	3.24	1.79	0.86	2.00E-20
0	SLC7A5	0.52	0.31	2.38	1.49	0.68	2.33E-20
0	USP47	0.46	0.28	1.89	0.99	0.93	2.36E-20
0	CXCR4	0.85	0.72	14.66	9.63	0.61	2.87E-20
0	PGRMC2	0.27	0.12	0.95	0.40	1.24	6.93E-20
0	BZW1	0.82	0.69	6.29	4.43	0.51	7.68E-20
0	H3-3A	0.94	0.87	12.52	9.57	0.39	8.46E-20
0	HNRNPC	0.86	0.72	6.72	5.01	0.43	1.33E-19
0	TMEM120B	0.47	0.29	1.82	0.98	0.89	1.43E-19
0	TRGC1	0.52	0.33	2.66	1.42	0.90	1.46E-19
0	CYLD	0.70	0.52	4.19	2.66	0.65	1.74E-19
0	CYSTM1	0.36	0.19	1.24	0.61	1.02	2.15E-19
0	MSN	0.79	0.69	6.80	4.59	0.57	2.64E-19
0	CNOT6L	0.83	0.68	7.65	5.43	0.49	3.03E-19
0	SERPINB9	0.64	0.49	6.09	3.32	0.87	3.04E-19
0	TNFRSF4	0.27	0.12	1.29	0.50	1.37	3.50E-19
0	RHOF	0.58	0.41	2.71	1.61	0.75	6.14E-19
0	CPNE1	0.43	0.25	1.57	0.92	0.78	6.36E-19
0	ASAP1	0.26	0.11	0.83	0.33	1.33	7.39E-19
0	SYAP1	0.62	0.43	3.55	2.23	0.67	8.22E-19
0	RANBP2	0.55	0.36	2.63	1.61	0.71	1.22E-18
0	ELL2	0.48	0.29	2.00	1.23	0.69	1.73E-18
0	AGPAT4	0.32	0.16	1.15	0.58	0.98	1.80E-18
0	LYST	0.62	0.44	3.75	2.50	0.59	2.06E-18
0	PIM2	0.36	0.19	1.26	0.67	0.91	2.07E-18

0	RUNX3	0.87	0.74	8.22	5.99	0.46	3.90E-18
0	OTULIN	0.58	0.42	3.17	1.79	0.83	4.76E-18
0	FTH1	0.97	0.96	62.43	34.80	0.84	6.39E-18
0	NAP1L1	0.72	0.57	4.56	3.02	0.59	1.37E-17
0	SIK3	0.51	0.33	2.26	1.36	0.73	1.64E-17
0	FXYD5	0.68	0.52	3.61	2.36	0.62	1.68E-17
0	JARID2	0.50	0.32	2.12	1.22	0.80	3.85E-17
0	MCTP2	0.50	0.32	2.31	1.43	0.70	4.14E-17
0	TRAF4	0.33	0.18	1.10	0.60	0.88	9.35E-17
0	IL21R	0.37	0.21	1.36	0.75	0.86	1.07E-16
0	STK4	0.91	0.84	10.39	7.88	0.40	2.06E-16
0	GNA13	0.54	0.37	2.39	1.50	0.67	2.50E-16
0	MRPL14	0.33	0.18	1.08	0.59	0.87	2.81E-16
0	KMT2E	0.87	0.77	7.85	6.02	0.38	3.25E-16
0	COTL1	0.51	0.34	2.99	1.92	0.64	4.73E-16
0	SKI	0.36	0.20	1.24	0.72	0.78	6.63E-16
0	PLP2	0.41	0.25	1.50	0.87	0.79	7.00E-16
0	EIF5B	0.51	0.35	2.43	1.46	0.74	7.23E-16
0	CHD4	0.56	0.41	2.76	1.76	0.65	9.44E-16
0	IRF8	0.45	0.28	2.30	1.41	0.70	9.53E-16
0	LARP1	0.39	0.23	1.49	0.76	0.97	1.12E-15
0	FAM107B	0.65	0.51	4.68	2.96	0.66	1.72E-15
0	AKNA	0.64	0.49	3.63	2.44	0.57	2.43E-15
0	PAK2	0.53	0.37	2.47	1.56	0.66	2.45E-15
0	HIVEP2	0.34	0.19	1.18	0.65	0.85	2.64E-15
0	QKI	0.50	0.34	2.21	1.31	0.75	2.80E-15
0	GABARAPL	0.77	0.63	5.67	4.02	0.50	4.20E-15
0	UBE2F	0.42	0.28	1.87	1.03	0.86	4.51E-15
0	GOLGB1	0.65	0.50	4.34	2.90	0.58	5.51E-15
0	GALNT11	0.31	0.17	1.07	0.58	0.88	6.83E-15
0	RPL23	0.87	0.79	8.99	6.69	0.43	7.94E-15
0	HDGF	0.51	0.34	2.05	1.32	0.63	1.10E-14
0	RAP1B	0.81	0.69	5.99	4.57	0.39	1.10E-14
0	HBS1L	0.31	0.17	1.05	0.55	0.94	1.53E-14
0	TNIP1	0.39	0.24	1.50	0.87	0.79	2.19E-14
0	TAB2	0.28	0.15	0.87	0.47	0.87	2.25E-14
0	RALA	0.35	0.20	1.19	0.69	0.78	3.63E-14
0	CDC37	0.66	0.53	3.81	2.59	0.56	4.36E-14
0	NOTCH2NL	0.28	0.15	0.94	0.51	0.88	4.39E-14
0	MBP	0.69	0.55	4.55	3.26	0.48	4.50E-14
0	CBLB	0.49	0.32	2.13	1.37	0.64	4.92E-14
0	STAT4	0.53	0.38	2.58	1.68	0.62	5.07E-14
0	BRAF	0.39	0.24	1.50	0.86	0.80	6.60E-14
0	HDLBP	0.28	0.15	0.97	0.51	0.93	6.85E-14
0	PRRC2C	0.84	0.75	7.14	5.43	0.40	6.95E-14
0	ZC3H18	0.35	0.21	1.13	0.63	0.85	9.29E-14
0	HIF1A	0.59	0.42	2.83	1.98	0.51	9.40E-14
0	ID2	0.74	0.64	8.41	5.74	0.55	9.48E-14
0	CNOT2	0.53	0.40	2.87	1.73	0.73	1.23E-13
0	TES	0.48	0.33	2.20	1.37	0.69	1.82E-13
0	BCL6	0.34	0.20	1.29	0.74	0.80	2.38E-13
0	NFKBIZ	0.51	0.36	2.93	1.95	0.59	2.76E-13
0	STK17A	0.85	0.75	7.69	5.80	0.41	3.12E-13
0	ARID4B	0.76	0.63	5.16	3.88	0.41	3.17E-13
0	TRAF5	0.30	0.17	1.13	0.62	0.86	4.19E-13
0	NR4A3	0.27	0.15	1.00	0.53	0.93	5.11E-13
0	LDHA	0.79	0.67	6.54	4.60	0.51	5.75E-13
0	DHRS3	0.27	0.15	0.93	0.53	0.80	5.93E-13
0	PBXIP1	0.36	0.23	1.52	0.86	0.83	8.92E-13

0	CMTM6	0.51	0.36	2.00	1.32	0.60	9.87E-13
0	TUBA4A	0.79	0.65	9.35	6.90	0.44	9.91E-13
0	RPL13A	0.91	0.86	21.63	16.41	0.40	1.15E-12
0	YBX3	0.37	0.23	2.27	1.33	0.78	1.19E-12
0	DAAM1	0.27	0.15	1.12	0.58	0.94	1.53E-12
0	ZNF331	0.63	0.47	4.63	3.46	0.42	1.60E-12
0	PRDX6	0.57	0.43	2.82	1.79	0.66	1.61E-12
0	GPR65	0.60	0.45	3.33	2.26	0.56	1.84E-12
0	RAB8B	0.81	0.71	6.76	5.12	0.40	2.02E-12
0	ACTN4	0.64	0.48	3.20	2.21	0.53	2.03E-12
0	RAD21	0.57	0.42	2.61	1.77	0.56	2.37E-12
0	RBM17	0.41	0.26	1.50	0.93	0.69	2.53E-12
0	RHOG	0.56	0.40	2.32	1.61	0.52	2.53E-12
0	RALGDS	0.35	0.22	1.29	0.74	0.80	3.00E-12
0	LPIN1	0.34	0.21	1.24	0.72	0.78	3.23E-12
0	S100A11	0.69	0.57	4.92	3.58	0.46	3.49E-12
0	PIM3	0.62	0.46	3.32	2.40	0.47	3.69E-12
0	AKAP13	0.79	0.72	6.68	4.99	0.42	3.88E-12
0	SACM1L	0.42	0.29	1.56	1.00	0.64	4.79E-12
0	CRYBG1	0.40	0.26	1.73	0.99	0.81	4.96E-12
0	SLFN13	0.32	0.19	1.11	0.70	0.66	5.02E-12
0	PABPC4	0.36	0.22	1.21	0.74	0.71	5.38E-12
0	PHF20	0.58	0.45	3.02	2.06	0.55	7.14E-12
0	USP12	0.35	0.22	1.31	0.76	0.78	9.31E-12
0	RSL1D1	0.50	0.37	1.98	1.31	0.60	9.51E-12
0	ST3GAL1	0.34	0.21	1.26	0.72	0.80	1.08E-11
0	ETS1	0.76	0.63	5.16	3.91	0.40	1.21E-11
0	ABHD17B	0.25	0.14	0.78	0.40	0.96	1.36E-11
0	SEC11A	0.55	0.40	2.16	1.54	0.48	1.37E-11
0	CTSZ	0.25	0.14	0.79	0.44	0.86	1.82E-11
0	SURF4	0.44	0.29	1.63	1.06	0.63	2.61E-11
0	POLR2L	0.54	0.40	2.35	1.58	0.57	2.72E-11
0	KDM2B	0.25	0.14	0.76	0.43	0.81	2.97E-11
0	SPRY1	0.28	0.16	1.40	1.09	0.36	2.97E-11
0	CD63	0.75	0.63	4.64	3.59	0.37	3.78E-11
0	IPO7	0.29	0.18	0.95	0.54	0.81	4.49E-11
0	TUT4	0.49	0.35	2.00	1.37	0.55	4.90E-11
0	MARCHF7	0.37	0.24	1.33	0.84	0.66	5.41E-11
0	CARD19	0.32	0.20	1.13	0.71	0.67	5.99E-11
0	TIPARP	0.51	0.37	2.31	1.69	0.46	6.37E-11
0	CSNK1D	0.51	0.35	1.93	1.38	0.48	7.01E-11
0	PTK2B	0.32	0.20	1.41	0.72	0.96	8.29E-11
0	OTUD5	0.31	0.19	1.06	0.63	0.75	9.34E-11
0	CCDC93	0.29	0.18	1.06	0.61	0.81	1.06E-10
0	WDR48	0.29	0.18	0.92	0.53	0.80	1.09E-10
0	KTN1	0.61	0.48	3.11	2.20	0.50	1.10E-10
0	HOOK3	0.29	0.17	0.99	0.58	0.77	1.14E-10
0	USP15	0.56	0.44	2.84	1.94	0.55	1.27E-10
0	PDXK	0.28	0.16	0.87	0.50	0.78	1.32E-10
0	PCMTD1	0.37	0.23	1.31	0.84	0.65	1.41E-10
0	PDCD4	0.57	0.42	2.85	2.11	0.44	1.81E-10
0	AHR	0.32	0.20	1.18	0.87	0.44	2.10E-10
0	IRF2BP2	0.49	0.34	2.04	1.47	0.47	2.12E-10
0	EDF1	0.76	0.67	4.62	3.54	0.39	2.21E-10
0	ARRDC2	0.27	0.16	0.88	0.53	0.74	2.34E-10
0	CMIP	0.40	0.27	1.62	1.16	0.48	2.43E-10
0	PSMD7	0.33	0.20	1.09	0.67	0.71	2.51E-10
0	SOS2	0.32	0.20	1.09	0.68	0.69	2.66E-10
0	LRPAP1	0.31	0.18	0.91	0.59	0.63	2.93E-10

0	RAB9A	0.49	0.37	2.44	1.54	0.66	3.30E-10
0	SINHCAF	0.29	0.18	0.95	0.58	0.71	4.25E-10
0	LAPTM4A	0.42	0.28	1.55	1.06	0.55	4.71E-10
0	STT3B	0.37	0.24	1.20	0.80	0.59	5.81E-10
0	EHD1	0.38	0.25	1.43	0.95	0.59	5.92E-10
0	SLC25A36	0.35	0.23	1.21	0.77	0.66	6.52E-10
0	HCG18	0.33	0.21	1.20	0.78	0.63	6.60E-10
0	SPAG9	0.48	0.35	2.08	1.39	0.58	6.94E-10
0	NR3C1	0.67	0.55	3.97	3.04	0.39	7.09E-10
0	BIRC2	0.44	0.31	1.77	1.26	0.50	7.12E-10
0	EIF4G2	0.73	0.62	4.27	3.26	0.39	9.11E-10
0	FNBP1	0.69	0.57	4.72	3.48	0.44	9.66E-10
0	PRMT1	0.38	0.26	1.37	0.89	0.62	1.10E-09
0	LFNG	0.30	0.19	1.03	0.67	0.62	1.32E-09
0	LAT2	0.38	0.26	1.48	1.01	0.54	1.74E-09
0	IL18RAP	0.30	0.19	1.24	0.76	0.70	1.98E-09
0	RPS6KA3	0.38	0.27	1.54	1.01	0.61	2.00E-09
0	SEPTIN11	0.27	0.16	0.95	0.54	0.80	2.16E-09
0	ZCCHC2	0.36	0.24	1.23	0.86	0.51	2.17E-09
0	CSNK2A1	0.28	0.17	0.92	0.59	0.65	2.35E-09
0	CLPP	0.26	0.16	0.82	0.45	0.85	2.53E-09
0	APMAP	0.61	0.46	2.88	2.24	0.37	3.30E-09
0	MAPKAPK2	0.46	0.33	1.73	1.22	0.50	4.34E-09
0	DDX21	0.64	0.53	3.80	2.88	0.40	4.34E-09
0	NPEPPS	0.33	0.22	1.06	0.73	0.53	4.40E-09
0	TRABD	0.40	0.27	1.48	1.02	0.54	4.72E-09
0	TMBIM6	0.70	0.58	3.70	2.88	0.36	5.18E-09
0	IFNGR1	0.46	0.33	1.99	1.45	0.46	5.30E-09
0	GAS5	0.64	0.53	3.36	2.59	0.38	5.46E-09
0	ZNF267	0.39	0.26	1.47	0.96	0.62	6.49E-09
0	CD300A	0.46	0.35	2.46	1.59	0.63	6.65E-09
0	RPS17	0.52	0.40	2.16	1.58	0.45	7.08E-09
0	CEBPD	0.37	0.25	2.30	1.64	0.49	7.67E-09
0	UBE2M	0.42	0.29	1.44	1.02	0.50	1.12E-08
0	NDUFS5	0.60	0.47	2.77	2.08	0.41	1.16E-08
0	UBE2I	0.51	0.37	1.85	1.42	0.38	1.22E-08
0	ARHGEF2	0.32	0.21	1.11	0.69	0.68	1.25E-08
0	PDE7A	0.41	0.29	1.61	1.10	0.54	1.45E-08
0	KMT2C	0.50	0.38	2.28	1.69	0.43	1.76E-08
0	NARF	0.32	0.21	1.15	0.69	0.73	1.76E-08
0	RELA	0.27	0.17	0.92	0.54	0.76	1.89E-08
0	HMGA1	0.33	0.22	1.05	0.72	0.54	2.07E-08
0	POLR2K	0.48	0.36	2.03	1.47	0.46	2.29E-08
0	NBEAL1	0.26	0.16	0.87	0.53	0.71	2.43E-08
0	FLNA	0.70	0.59	4.51	3.35	0.43	2.50E-08
0	IL10RA	0.48	0.37	2.23	1.49	0.58	2.58E-08
0	ZC3H7A	0.30	0.19	0.95	0.63	0.61	3.12E-08
0	FMNL1	0.64	0.51	3.24	2.38	0.44	3.42E-08
0	MAFF	0.36	0.24	1.43	1.10	0.38	3.58E-08
0	ARFGAP3	0.42	0.31	1.66	1.16	0.52	4.24E-08
0	ZNF706	0.44	0.33	1.69	1.17	0.53	4.37E-08
0	ELOVL5	0.48	0.37	2.05	1.48	0.47	4.40E-08
0	CLINT1	0.39	0.27	1.44	0.96	0.58	4.43E-08
0	NIBAN1	0.28	0.19	1.23	0.72	0.79	5.14E-08
0	AKAP17A	0.38	0.28	1.44	0.90	0.67	5.18E-08
0	CEBPZ	0.42	0.31	1.53	1.10	0.48	5.31E-08
0	EIF3E	0.61	0.49	2.78	2.15	0.37	5.32E-08
0	N4BP1	0.35	0.24	1.27	0.91	0.48	5.35E-08
0	MORF4L2	0.48	0.35	1.85	1.39	0.41	5.87E-08

0	NFKBID	0.35	0.25	1.65	1.13	0.55	6.71E-08
0	ATP1B3	0.66	0.56	6.78	4.92	0.46	7.57E-08
0	TUBB	0.60	0.47	2.91	2.24	0.38	7.95E-08
0	TM9SF3	0.44	0.33	1.65	1.18	0.49	8.67E-08
0	CAST	0.61	0.51	4.16	2.81	0.57	9.28E-08
0	RNF145	0.35	0.25	1.22	0.84	0.54	9.39E-08
0	GSTO1	0.38	0.26	1.35	0.98	0.46	9.43E-08
0	FTX	0.28	0.18	1.01	0.62	0.70	9.70E-08
0	CIAO2B	0.43	0.32	1.63	1.11	0.56	1.02E-07
0	SEMA4D	0.46	0.34	1.90	1.35	0.50	1.03E-07
0	ASH1L	0.48	0.36	2.11	1.55	0.45	1.04E-07
0	TACC1	0.44	0.33	1.79	1.26	0.51	1.06E-07
0	LUC7L	0.32	0.21	1.01	0.72	0.49	1.13E-07
0	NIPBL	0.47	0.36	1.95	1.44	0.43	1.30E-07
0	THRAP3	0.48	0.35	1.86	1.40	0.41	1.48E-07
0	USP1	0.27	0.18	0.90	0.59	0.59	1.48E-07
0	ITM2A	0.45	0.35	2.60	1.80	0.53	1.62E-07
0	CBX6	0.26	0.16	0.85	0.54	0.65	1.78E-07
0	MIR23AHG	0.48	0.38	4.31	2.70	0.68	1.84E-07
0	SETD5	0.42	0.31	1.70	1.20	0.50	1.86E-07
0	VAPA	0.56	0.46	2.39	1.79	0.41	2.27E-07
0	BCLAF1	0.55	0.44	2.67	2.04	0.38	2.29E-07
0	UBA6	0.28	0.18	0.87	0.57	0.59	2.48E-07
0	EIF1AX	0.53	0.42	2.63	1.95	0.43	2.85E-07
0	GYPC	0.45	0.34	1.91	1.42	0.43	2.93E-07
0	PHF1	0.40	0.29	1.39	1.00	0.48	2.93E-07
0	CAB39	0.26	0.17	0.81	0.54	0.58	2.95E-07
0	OXSR1	0.27	0.18	0.83	0.60	0.47	3.62E-07
0	SMARCC1	0.27	0.18	0.86	0.60	0.51	3.66E-07
0	HSP90B1	0.75	0.67	6.02	4.60	0.39	4.08E-07
0	ANP32A	0.47	0.36	1.86	1.36	0.45	4.25E-07
0	PA2G4	0.50	0.39	2.11	1.56	0.44	4.42E-07
0	APLP2	0.37	0.27	1.41	0.99	0.51	4.53E-07
0	EIF5A	0.57	0.45	2.50	1.91	0.39	5.82E-07
0	RWDD1	0.40	0.29	1.55	1.08	0.52	6.04E-07
0	PUM1	0.38	0.28	1.30	0.93	0.49	6.23E-07
0	EIF3J	0.43	0.33	1.89	1.33	0.51	7.41E-07
0	PCMT1	0.32	0.22	1.00	0.72	0.47	7.64E-07
0	FBL	0.35	0.25	1.13	0.78	0.52	7.82E-07
0	HUWE1	0.43	0.32	1.69	1.26	0.43	8.05E-07
0	RNPS1	0.43	0.32	1.57	1.13	0.48	8.63E-07
0	H2AJ	0.50	0.40	2.87	1.96	0.55	8.90E-07
0	BEX4	0.27	0.17	0.83	0.60	0.47	9.18E-07
0	SLC39A10	0.34	0.24	1.37	0.95	0.53	9.55E-07
0	SAFB2	0.37	0.27	1.31	0.95	0.46	1.01E-06
0	PRKAR1A	0.45	0.35	1.76	1.29	0.45	1.06E-06
0	NPAT	0.28	0.19	0.87	0.65	0.42	1.08E-06
0	DHX9	0.32	0.23	1.09	0.78	0.48	1.21E-06
0	CSNK1G3	0.34	0.24	1.08	0.81	0.41	1.27E-06
0	TXNRD1	0.32	0.23	1.32	0.90	0.56	1.34E-06
0	NAP1L4	0.52	0.41	2.32	1.78	0.38	1.37E-06
0	TENT4B	0.39	0.29	1.53	1.13	0.43	1.53E-06
0	SEPTIN2	0.35	0.26	1.20	0.85	0.50	1.57E-06
0	MED13	0.40	0.29	1.42	1.05	0.43	1.58E-06
0	YARS1	0.32	0.22	1.07	0.73	0.55	1.63E-06
0	MAT2A	0.37	0.27	1.41	1.06	0.42	1.70E-06
0	RPL7L1	0.33	0.24	1.09	0.77	0.51	1.76E-06
0	DDAH2	0.30	0.21	1.08	0.73	0.56	1.97E-06
0	UBE2R2	0.36	0.26	1.16	0.89	0.39	2.33E-06

0	PSME4	0.36	0.26	1.26	0.94	0.41	2.37E-06
0	CLK4	0.26	0.17	0.76	0.55	0.46	2.61E-06
0	CSNK1A1	0.41	0.31	1.44	1.05	0.45	2.63E-06
0	CUL3	0.35	0.25	1.21	0.85	0.52	2.76E-06
0	KAT6A	0.42	0.32	1.56	1.19	0.39	2.92E-06
0	KCTD20	0.27	0.19	0.89	0.63	0.50	2.96E-06
0	AHNAK	0.58	0.50	3.68	2.75	0.42	2.97E-06
0	PLEKHO1	0.32	0.22	1.07	0.81	0.40	3.02E-06
0	GNL3	0.32	0.23	1.07	0.82	0.38	3.24E-06
0	RANGAP1	0.30	0.21	1.05	0.73	0.52	3.60E-06
0	MAPK1IP1L	0.28	0.19	0.85	0.60	0.52	3.77E-06
0	SETD2	0.45	0.36	1.89	1.41	0.42	4.12E-06
0	RNF19A	0.72	0.66	6.23	4.85	0.36	4.33E-06
0	TAX1BP1	0.59	0.50	3.01	2.32	0.38	4.57E-06
0	MAT2B	0.29	0.20	0.95	0.68	0.48	5.50E-06
0	TNRC6B	0.48	0.39	2.37	1.76	0.43	5.58E-06
0	SELENOS	0.37	0.27	1.37	1.03	0.40	5.73E-06
0	COPB1	0.31	0.22	1.08	0.75	0.53	8.60E-06
0	IL27RA	0.27	0.18	0.86	0.62	0.48	1.07E-05
0	TTC39C	0.30	0.22	1.10	0.74	0.56	1.12E-05
0	LINC01138	0.27	0.19	1.03	0.78	0.41	1.12E-05
0	OFD1	0.54	0.44	3.01	2.08	0.53	1.19E-05
0	CRCP	0.28	0.19	0.83	0.62	0.44	1.21E-05
0	RELB	0.45	0.35	1.80	1.36	0.40	1.23E-05
0	SRPK1	0.29	0.21	0.97	0.71	0.46	1.35E-05
0	EIF2AK3	0.27	0.19	1.04	0.74	0.49	1.37E-05
0	CCNT1	0.30	0.22	1.02	0.71	0.52	1.39E-05
0	G3BP2	0.59	0.50	3.09	2.35	0.39	1.46E-05
0	MACROH2A	0.25	0.18	0.80	0.55	0.55	1.67E-05
0	LAMTOR5	0.34	0.25	1.09	0.82	0.40	1.92E-05
0	DYNLT1	0.29	0.20	0.98	0.71	0.46	1.96E-05
0	PIK3AP1	0.30	0.22	1.10	0.73	0.60	2.07E-05
0	BDP1	0.42	0.33	1.83	1.42	0.37	2.12E-05
0	KPNA4	0.44	0.35	1.70	1.27	0.42	2.26E-05
0	DNAJC1	0.47	0.37	1.90	1.46	0.38	2.36E-05
0	ZC3H15	0.42	0.33	1.62	1.22	0.40	2.67E-05
0	RLF	0.33	0.25	1.18	0.90	0.39	2.80E-05
0	FLOT1	0.26	0.18	0.81	0.59	0.46	3.10E-05
0	ATP11B	0.33	0.25	1.14	0.81	0.50	3.45E-05
0	PCNP	0.36	0.27	1.17	0.89	0.39	3.87E-05
0	TMED2	0.26	0.19	0.80	0.59	0.44	4.77E-05
0	WDR82	0.38	0.29	1.31	1.00	0.39	4.98E-05
0	ETF1	0.41	0.33	1.51	1.17	0.37	5.43E-05
0	SCFD1	0.28	0.20	0.93	0.70	0.41	6.30E-05
0	POLE3	0.29	0.21	0.97	0.73	0.40	6.34E-05
0	C1QBP	0.27	0.19	0.88	0.63	0.49	6.70E-05
0	EPB41L4A-/	0.34	0.25	1.05	0.81	0.38	6.84E-05
0	C11orf58	0.46	0.37	1.83	1.42	0.36	7.03E-05
0	PPA1	0.32	0.24	1.10	0.83	0.40	7.44E-05
0	SMC4	0.29	0.21	1.06	0.80	0.39	7.57E-05
0	FYTTD1	0.33	0.25	1.10	0.82	0.41	7.77E-05
0	CELF1	0.32	0.24	1.08	0.84	0.37	7.85E-05
0	CCT8	0.36	0.28	1.23	0.95	0.38	8.01E-05
0	RSF1	0.38	0.30	1.47	1.13	0.38	8.60E-05
0	IRF7	0.26	0.19	0.98	0.66	0.57	9.03E-05
0	TNRC6C	0.36	0.28	1.38	1.04	0.41	9.57E-05
0	PPM1G	0.41	0.33	1.60	1.22	0.40	9.85E-05
0	ENO1	0.57	0.50	2.88	2.24	0.36	0.00010431
0	H1-4	0.33	0.25	1.40	1.07	0.39	0.00011201

0	DDX27	0.31	0.23	1.12	0.82	0.45	0.00012746
0	MRFAP1	0.47	0.38	1.75	1.34	0.39	0.00013098
0	RTF2	0.27	0.20	0.86	0.63	0.46	0.00015176
0	NT5C3A	0.26	0.19	0.79	0.61	0.37	0.00015349
0	ZFC3H1	0.40	0.32	1.62	1.23	0.39	0.00017484
0	BRD4	0.44	0.35	1.65	1.28	0.37	0.00022065
0	BNIP3L	0.30	0.24	1.15	0.82	0.48	0.00022671
0	TOR1AIP2	0.29	0.21	0.91	0.71	0.36	0.00023246
0	SIVA1	0.32	0.25	1.07	0.80	0.41	0.00025112
0	CPD	0.33	0.26	1.28	0.97	0.40	0.0003038
0	CDK17	0.28	0.21	0.98	0.70	0.47	0.00031372
0	TMEM243	0.30	0.22	1.00	0.76	0.39	0.00034123
0	USP9X	0.27	0.20	0.85	0.66	0.37	0.0004343
0	RB1CC1	0.29	0.22	1.02	0.77	0.39	0.00046941
0	COX5A	0.37	0.31	1.30	0.99	0.40	0.00060783
0	SAR1B	0.28	0.22	1.00	0.73	0.46	0.00060854
0	CD83	0.28	0.22	1.79	1.31	0.45	0.00062001
0	SH2D1A	0.43	0.37	2.18	1.58	0.46	0.00069527
0	SERINC3	0.26	0.19	0.81	0.63	0.37	0.00085305
0	INPP5D	0.32	0.25	1.17	0.90	0.38	0.00093172
0	RANBP1	0.27	0.21	0.93	0.71	0.39	0.00136047
0	UBR5	0.30	0.23	1.01	0.78	0.37	0.00144087
0	UQCRC2	0.29	0.23	0.97	0.76	0.36	0.00144527
0	HSH2D	0.28	0.22	1.13	0.87	0.38	0.00148124
0	INSIG1	0.39	0.33	2.46	1.59	0.63	0.00189876
0	CWC25	0.28	0.22	0.92	0.71	0.37	0.00206462
0	SLA2	0.33	0.27	1.41	1.10	0.37	0.0039543
0	CEMIP2	0.55	0.52	4.59	3.39	0.44	0.00517124
0	RAB5A	0.25	0.20	0.81	0.60	0.44	0.00558094
1	FGFBP2	0.71	0.14	6.11	1.10	2.47	2.58E-139
1	NKG7	0.99	0.91	44.83	18.03	1.31	8.41E-111
1	FCGR3A	0.75	0.25	5.76	1.53	1.91	1.53E-97
1	GZMH	0.78	0.29	7.48	2.05	1.87	4.94E-96
1	S1PR5	0.53	0.13	2.64	0.53	2.32	2.78E-81
1	RORA	0.80	0.44	7.86	2.89	1.44	2.05E-64
1	PPP2R5C	0.83	0.53	7.35	2.83	1.38	1.26E-63
1	SYNE2	0.75	0.36	5.90	2.17	1.44	2.06E-59
1	CCL5	0.96	0.78	36.09	18.12	0.99	4.15E-59
1	GZMB	0.96	0.62	20.72	12.50	0.73	5.53E-59
1	B2M	1.00	1.00	131.57	89.35	0.56	6.95E-59
1	PRDM1	0.56	0.20	3.11	1.01	1.62	1.04E-57
1	DSTN	0.62	0.28	4.22	1.16	1.86	1.42E-57
1	TGFBR3	0.42	0.12	2.14	0.43	2.32	4.37E-55
1	PYHIN1	0.54	0.20	3.02	0.85	1.83	7.73E-55
1	CST7	0.93	0.76	15.05	6.97	1.11	4.68E-53
1	HLA-C	0.99	0.97	29.55	18.82	0.65	1.75E-52
1	PRF1	0.80	0.52	8.30	3.21	1.37	3.73E-52
1	HLA-E	0.97	0.91	17.87	10.51	0.77	6.70E-52
1	HLA-B	1.00	0.99	48.36	33.05	0.55	5.54E-50
1	IGF2R	0.58	0.26	3.07	0.95	1.69	2.41E-49
1	ZEB2	0.83	0.57	9.34	4.09	1.19	5.49E-49
1	GNG2	0.75	0.41	6.17	2.47	1.32	4.30E-48
1	GTF3C1	0.46	0.17	2.28	0.61	1.89	6.54E-47
1	PTPRC	0.98	0.91	22.45	13.37	0.75	3.25E-45
1	ENC1	0.34	0.09	1.48	0.36	2.02	5.54E-44
1	ARL4C	0.87	0.64	9.65	5.09	0.92	9.49E-43
1	SPON2	0.37	0.10	1.93	0.57	1.75	2.46E-42
1	PLEK	0.68	0.39	5.01	2.08	1.27	3.98E-40
1	TNFRSF1B	0.80	0.56	6.15	3.00	1.04	1.10E-39

1	FTL	0.98	0.97	46.89	25.33	0.89	9.95E-39
1	S100A6	0.84	0.66	9.88	5.01	0.98	1.43E-38
1	CCL4	0.82	0.49	32.30	20.88	0.63	3.16E-37
1	ABHD17A	0.75	0.51	4.78	2.28	1.07	3.69E-34
1	ZBTB38	0.38	0.14	1.64	0.51	1.70	3.79E-33
1	EFHD2	0.77	0.55	5.98	2.97	1.01	4.74E-33
1	TFDP2	0.37	0.14	1.60	0.48	1.74	5.23E-33
1	CYBA	0.89	0.75	11.04	6.27	0.82	1.11E-31
1	C12orf75	0.48	0.23	2.38	0.89	1.42	1.79E-31
1	ABI3	0.33	0.12	1.37	0.40	1.79	3.82E-31
1	DTHD1	0.28	0.09	1.39	0.31	2.17	1.03E-30
1	CTSC	0.65	0.40	4.19	2.02	1.05	3.16E-30
1	CD48	0.74	0.55	4.99	2.62	0.93	5.09E-29
1	CHST12	0.55	0.30	2.96	1.28	1.21	9.19E-29
1	HLA-A	0.98	0.96	29.21	21.01	0.48	1.09E-28
1	LGALS1	0.85	0.64	10.61	6.23	0.77	1.41E-28
1	ARPC2	0.92	0.83	10.21	6.47	0.66	2.83E-28
1	IFI16	0.77	0.58	6.10	3.23	0.92	8.56E-28
1	PTMS	0.39	0.17	2.02	0.72	1.49	1.59E-27
1	S100A4	0.78	0.61	9.18	4.81	0.93	8.59E-27
1	CCL4L2	0.47	0.22	11.80	5.78	1.03	2.83E-26
1	ITGB2	0.56	0.32	3.09	1.38	1.16	1.01E-25
1	PTP4A2	0.72	0.54	4.70	2.43	0.95	2.33E-25
1	GK5	0.33	0.14	1.58	0.46	1.79	3.47E-25
1	FGL2	0.26	0.09	1.39	0.44	1.67	1.17E-24
1	CELF2	0.53	0.32	3.07	1.26	1.28	1.22E-24
1	CLEC2D	0.65	0.44	5.58	2.66	1.07	2.83E-24
1	ORAI1	0.49	0.28	2.40	0.98	1.30	2.93E-24
1	PRKCH	0.57	0.36	2.84	1.35	1.07	3.06E-24
1	BCL11B	0.39	0.18	1.75	0.71	1.31	6.34E-24
1	SLC15A4	0.39	0.19	1.71	0.64	1.41	9.22E-24
1	F2R	0.29	0.11	1.09	0.37	1.58	1.23E-23
1	TMSB10	0.98	0.96	28.93	20.28	0.51	1.81E-23
1	SYNE1	0.46	0.24	2.48	1.00	1.31	4.03E-23
1	MT2A	0.65	0.44	10.88	5.31	1.03	4.88E-23
1	C1orf21	0.45	0.24	1.99	0.83	1.26	9.37E-23
1	ADGRG1	0.27	0.10	1.17	0.35	1.72	1.50E-22
1	KLF2	0.62	0.37	4.81	3.03	0.67	1.65E-22
1	ISG20	0.72	0.55	6.23	3.62	0.78	1.97E-22
1	GCLM	0.40	0.21	2.14	0.78	1.45	4.35E-22
1	RPL3	0.98	0.98	36.82	28.46	0.37	3.94E-21
1	IRF1-AS1	0.33	0.15	1.49	0.49	1.62	4.12E-21
1	MAF	0.32	0.14	1.54	0.61	1.33	2.02E-20
1	FKBP11	0.32	0.15	1.32	0.48	1.47	2.57E-20
1	LITAF	0.85	0.69	8.19	5.26	0.64	2.76E-20
1	MCOLN2	0.25	0.10	1.18	0.38	1.62	5.09E-20
1	PRKCB	0.26	0.10	1.02	0.31	1.71	6.75E-20
1	GNPTAB	0.43	0.23	2.23	0.95	1.22	9.72E-20
1	LPCAT1	0.28	0.12	1.03	0.39	1.40	1.44E-19
1	SLFN12L	0.46	0.26	2.38	1.09	1.13	1.77E-19
1	CD247	0.76	0.63	7.04	4.20	0.75	2.51E-19
1	IKZF3	0.54	0.33	3.13	1.84	0.76	1.76E-18
1	STAT5B	0.29	0.13	1.22	0.42	1.55	3.43E-18
1	ARID5B	0.55	0.36	3.42	1.90	0.85	6.06E-18
1	PBX4	0.27	0.12	1.15	0.41	1.47	8.85E-18
1	PCED1B-AS	0.49	0.31	2.34	1.12	1.06	9.13E-18
1	ERP29	0.48	0.31	2.00	1.00	1.00	1.44E-17
1	LYAR	0.36	0.18	1.48	0.67	1.13	2.77E-17
1	ADRB2	0.30	0.14	1.21	0.56	1.12	7.06E-17

1	MYL12B	0.83	0.72	7.03	4.73	0.57	8.77E-17
1	RAPGEF1	0.28	0.13	1.22	0.42	1.53	1.69E-16
1	TLE5	0.64	0.47	3.22	1.99	0.70	3.75E-16
1	SH3KBP1	0.42	0.26	1.81	0.89	1.03	1.15E-15
1	IQGAP2	0.52	0.36	2.79	1.39	1.01	1.54E-15
1	SLA	0.57	0.40	3.38	1.98	0.78	2.40E-15
1	SH3BGRL3	0.82	0.74	8.04	5.46	0.56	3.60E-15
1	MGAT4A	0.32	0.17	1.49	0.58	1.36	3.89E-15
1	ARL6IP5	0.78	0.65	6.53	4.21	0.64	9.20E-15
1	APMAP	0.65	0.49	3.65	2.21	0.72	1.16E-14
1	CAPN2	0.27	0.13	1.04	0.42	1.29	1.24E-14
1	CALM1	0.93	0.88	15.19	10.80	0.49	1.26E-14
1	LIMD2	0.58	0.41	2.75	1.63	0.75	2.67E-14
1	EPG5	0.27	0.13	1.16	0.46	1.33	2.87E-14
1	ARPC5	0.50	0.35	2.39	1.25	0.93	3.72E-14
1	MYO1F	0.32	0.17	1.36	0.65	1.07	7.39E-14
1	SELPLG	0.26	0.13	0.98	0.39	1.32	8.60E-14
1	MAN1A1	0.29	0.15	1.06	0.48	1.14	8.88E-14
1	CEP78	0.28	0.14	1.41	0.49	1.52	1.05E-13
1	SAMD3	0.62	0.46	3.59	2.23	0.69	1.19E-13
1	ITGAL	0.42	0.26	1.92	0.97	0.98	1.98E-13
1	TERF1	0.35	0.20	1.52	0.71	1.09	2.14E-13
1	NFATC2	0.33	0.19	1.18	0.59	0.98	2.77E-13
1	PFN1	0.87	0.84	10.42	7.41	0.49	3.13E-13
1	CYFIP2	0.31	0.17	1.21	0.53	1.18	3.25E-13
1	LBH	0.42	0.26	2.02	1.11	0.87	3.85E-13
1	GZMM	0.58	0.42	3.30	2.03	0.70	6.01E-13
1	TRBC1	0.52	0.38	3.31	1.80	0.88	7.86E-13
1	HLA-DPB1	0.56	0.41	2.67	1.73	0.63	8.57E-13
1	PDIA3	0.71	0.60	4.41	2.97	0.57	8.80E-13
1	LCP1	0.86	0.77	8.80	6.57	0.42	9.00E-13
1	ARRB2	0.30	0.16	1.13	0.50	1.18	1.01E-12
1	HAVCR2	0.43	0.28	2.40	1.23	0.96	1.59E-12
1	PPIB	0.64	0.49	3.27	2.13	0.62	1.65E-12
1	RASGRP1	0.33	0.19	1.61	0.66	1.30	1.75E-12
1	TRG-AS1	0.46	0.31	2.35	1.32	0.84	2.14E-12
1	RNF213	0.66	0.53	4.48	3.09	0.54	2.45E-12
1	PTPRE	0.34	0.20	1.47	0.70	1.07	3.22E-12
1	RAP1B	0.83	0.72	6.59	4.78	0.46	7.61E-12
1	APLP2	0.42	0.28	1.81	1.00	0.85	8.86E-12
1	ENSG00000	0.30	0.16	1.34	0.86	0.63	1.29E-11
1	YBX1	0.84	0.78	7.92	5.91	0.42	1.83E-11
1	LYN	0.35	0.22	1.64	0.73	1.17	2.04E-11
1	KLF3	0.45	0.31	2.17	1.20	0.85	2.46E-11
1	SUB1	0.80	0.73	5.86	4.30	0.45	3.69E-11
1	MXRA7	0.32	0.20	1.36	0.64	1.10	4.43E-11
1	DGKD	0.33	0.21	1.50	0.68	1.14	4.69E-11
1	RASA3	0.25	0.14	0.96	0.41	1.23	5.55E-11
1	ARNTL	0.26	0.14	1.00	0.48	1.06	5.75E-11
1	ARAP2	0.49	0.36	2.58	1.60	0.68	7.37E-11
1	LCK	0.40	0.28	1.66	0.92	0.85	8.64E-11
1	TMEM50A	0.55	0.44	2.70	1.68	0.68	9.25E-11
1	GAPDH	0.91	0.91	18.63	14.49	0.36	1.11E-10
1	FLNA	0.74	0.61	4.67	3.60	0.37	1.17E-10
1	PDIA6	0.39	0.26	1.60	0.88	0.85	1.19E-10
1	GIMAP7	0.37	0.23	1.66	0.98	0.76	1.71E-10
1	DIAPH1	0.56	0.42	2.61	1.71	0.61	2.03E-10
1	PGAM1	0.37	0.24	1.45	0.79	0.87	2.05E-10
1	CD99	0.60	0.48	3.26	2.18	0.58	2.57E-10

1	PTPN22	0.64	0.50	3.85	2.55	0.59	3.09E-10
1	PTPRA	0.29	0.17	1.05	0.59	0.83	3.13E-10
1	MYO1G	0.28	0.16	1.12	0.54	1.05	3.27E-10
1	FYN	0.79	0.69	6.45	4.68	0.46	3.82E-10
1	RRBP1	0.49	0.37	2.30	1.42	0.70	5.20E-10
1	AAK1	0.55	0.42	2.59	1.71	0.60	5.51E-10
1	HMGB2	0.58	0.45	3.85	2.56	0.59	5.54E-10
1	ARHGAP25	0.30	0.18	1.19	0.56	1.08	6.27E-10
1	TGFBR2	0.43	0.30	1.95	1.14	0.77	7.17E-10
1	PRR5L	0.30	0.18	1.25	0.62	1.01	8.53E-10
1	TBX21	0.34	0.22	1.45	0.79	0.88	1.12E-09
1	LBR	0.45	0.32	2.01	1.22	0.72	1.19E-09
1	MYH9	0.66	0.55	3.78	2.71	0.48	1.60E-09
1	GGA2	0.30	0.18	1.14	0.59	0.96	1.82E-09
1	CD53	0.69	0.59	4.19	2.90	0.53	2.06E-09
1	BCL7C	0.26	0.15	0.94	0.47	1.00	2.06E-09
1	DEK	0.60	0.48	3.29	2.19	0.59	2.38E-09
1	CEBPB	0.36	0.23	1.51	1.01	0.58	2.74E-09
1	GIMAP4	0.35	0.23	1.71	0.91	0.91	3.07E-09
1	APBB1IP	0.37	0.26	1.73	0.90	0.94	3.68E-09
1	VTI1B	0.25	0.15	0.98	0.42	1.20	3.88E-09
1	STK38	0.32	0.20	1.22	0.64	0.94	4.72E-09
1	METRNL	0.80	0.71	9.54	7.39	0.37	5.58E-09
1	PSAP	0.32	0.20	1.12	0.60	0.90	6.12E-09
1	EIF4G3	0.31	0.20	1.14	0.63	0.84	7.70E-09
1	STK10	0.37	0.25	1.47	0.88	0.75	1.11E-08
1	DUSP5	0.42	0.28	1.81	1.39	0.38	1.54E-08
1	GLRX	0.37	0.25	1.58	0.99	0.67	1.88E-08
1	UPP1	0.40	0.29	1.76	1.12	0.65	2.14E-08
1	ITK	0.30	0.20	1.29	0.64	1.02	2.19E-08
1	TNIP2	0.28	0.17	1.01	0.55	0.89	2.49E-08
1	LAPTM5	0.52	0.41	2.21	1.50	0.56	2.76E-08
1	DHRS7	0.39	0.28	1.67	0.99	0.75	3.33E-08
1	UQCRB	0.74	0.67	4.90	3.57	0.46	3.70E-08
1	WNK1	0.45	0.34	1.99	1.27	0.64	4.46E-08
1	RAB27A	0.45	0.33	2.04	1.26	0.69	5.09E-08
1	SYTL3	0.70	0.59	5.00	3.76	0.41	5.18E-08
1	SH2D2A	0.41	0.29	1.92	1.16	0.72	5.54E-08
1	APOL6	0.40	0.29	1.83	1.08	0.76	5.59E-08
1	GLIPR1	0.42	0.29	1.86	1.30	0.52	5.68E-08
1	PSMB8	0.52	0.42	2.37	1.57	0.60	6.07E-08
1	ADD3	0.26	0.16	1.02	0.53	0.95	6.17E-08
1	TUT7	0.36	0.25	1.40	0.84	0.73	9.10E-08
1	PITPNC1	0.38	0.27	1.59	1.02	0.64	1.02E-07
1	CCDC88C	0.36	0.25	1.56	0.90	0.79	1.14E-07
1	SDCBP	0.55	0.45	2.98	2.07	0.53	1.18E-07
1	ATP1B3	0.67	0.58	6.98	5.34	0.39	1.33E-07
1	EMP3	0.68	0.58	4.24	3.09	0.46	1.34E-07
1	ENSG00000	0.36	0.25	1.48	0.91	0.71	1.40E-07
1	SSR2	0.62	0.54	3.14	2.21	0.51	1.52E-07
1	SLC9A3R1	0.37	0.25	1.44	0.97	0.58	2.13E-07
1	SRPK2	0.28	0.18	1.05	0.56	0.92	2.23E-07
1	OSBPL8	0.43	0.32	1.94	1.26	0.62	2.75E-07
1	MORC3	0.38	0.27	1.43	0.90	0.67	2.90E-07
1	HCLS1	0.41	0.30	1.61	1.04	0.63	2.94E-07
1	TNFRSF14	0.27	0.17	1.03	0.54	0.94	3.00E-07
1	IRF2	0.25	0.16	0.95	0.55	0.78	3.23E-07
1	MYL12A	0.76	0.67	6.09	4.67	0.38	4.10E-07
1	CD47	0.36	0.26	1.54	0.88	0.80	4.13E-07

1	SLA2	0.38	0.27	1.66	1.12	0.57	4.15E-07
1	PTPN12	0.28	0.19	1.14	0.59	0.94	4.94E-07
1	DIP2A	0.30	0.21	1.44	0.76	0.91	5.07E-07
1	SP140	0.27	0.17	0.92	0.55	0.74	5.55E-07
1	TMEM59	0.53	0.44	2.41	1.60	0.59	5.93E-07
1	RO60	0.31	0.22	1.28	0.70	0.87	6.03E-07
1	SPCS2	0.46	0.35	1.92	1.27	0.60	6.26E-07
1	ANKRD12	0.65	0.57	4.92	3.52	0.48	6.82E-07
1	MOB2	0.29	0.19	1.05	0.58	0.84	7.15E-07
1	STAT5A	0.33	0.23	1.45	0.92	0.66	7.36E-07
1	SPATA13	0.25	0.16	0.90	0.48	0.89	7.49E-07
1	TMA7	0.77	0.70	5.42	4.18	0.38	8.80E-07
1	CCNI	0.73	0.67	4.88	3.69	0.40	9.41E-07
1	CD3E	0.31	0.21	1.40	0.80	0.80	9.65E-07
1	UBL3	0.31	0.21	1.12	0.67	0.75	1.16E-06
1	PIP4K2A	0.53	0.45	2.74	1.78	0.62	1.33E-06
1	LSM14A	0.40	0.30	1.60	1.00	0.69	1.34E-06
1	OPTN	0.53	0.45	2.74	1.91	0.52	1.47E-06
1	CMC1	0.59	0.50	5.13	3.62	0.51	1.49E-06
1	NSD3	0.60	0.53	3.36	2.48	0.44	1.77E-06
1	ZBTB1	0.49	0.40	2.22	1.55	0.52	1.80E-06
1	TCF25	0.62	0.55	3.65	2.54	0.52	1.81E-06
1	MANF	0.33	0.24	1.35	0.77	0.81	1.84E-06
1	RAP2B	0.35	0.25	1.43	0.88	0.70	1.89E-06
1	SEC11C	0.27	0.18	1.04	0.60	0.81	1.90E-06
1	ARHGEF3	0.29	0.20	1.15	0.70	0.72	2.11E-06
1	SIRT2	0.33	0.23	1.28	0.79	0.69	2.20E-06
1	PKM	0.44	0.35	2.23	1.44	0.63	2.24E-06
1	PTPN4	0.26	0.17	1.09	0.57	0.94	2.27E-06
1	MBP	0.68	0.59	4.61	3.57	0.37	2.32E-06
1	ISCU	0.37	0.27	1.47	0.91	0.69	2.42E-06
1	PPP1R16B	0.36	0.26	1.38	0.89	0.64	2.57E-06
1	CMIP	0.40	0.30	1.86	1.22	0.61	2.72E-06
1	PSMA3	0.34	0.24	1.25	0.79	0.66	2.90E-06
1	BLOC1S1	0.27	0.18	0.93	0.57	0.70	3.03E-06
1	SLAMF7	0.40	0.30	1.81	1.22	0.57	3.06E-06
1	AUTS2	0.29	0.20	1.25	0.75	0.74	3.18E-06
1	MAPRE2	0.47	0.37	2.26	1.56	0.54	3.68E-06
1	MTPN	0.50	0.41	2.25	1.56	0.53	4.21E-06
1	TRAPPC10	0.42	0.34	1.86	1.19	0.65	5.20E-06
1	CCND3	0.34	0.24	1.34	0.85	0.66	5.30E-06
1	PRMT2	0.37	0.28	1.69	1.02	0.72	5.39E-06
1	HNRNPR	0.53	0.46	2.56	1.77	0.53	5.86E-06
1	ADAM10	0.27	0.19	1.03	0.57	0.85	6.19E-06
1	ATP6V0E1	0.59	0.52	2.93	2.12	0.47	6.40E-06
1	ANXA2	0.41	0.32	1.96	1.29	0.60	6.53E-06
1	MGAT1	0.33	0.23	1.27	0.80	0.66	6.70E-06
1	MYBL1	0.37	0.27	1.86	1.21	0.63	7.36E-06
1	TRAM1	0.38	0.30	1.59	1.03	0.63	8.51E-06
1	WASF2	0.43	0.35	1.85	1.22	0.60	9.46E-06
1	C1orf35	0.30	0.21	1.18	0.73	0.70	1.02E-05
1	RNF166	0.31	0.23	1.27	0.77	0.72	1.16E-05
1	KLRF1	0.41	0.31	1.97	1.43	0.46	1.23E-05
1	NAP1L4	0.52	0.44	2.56	1.86	0.46	1.36E-05
1	RHOC	0.31	0.22	1.10	0.69	0.66	1.64E-05
1	NDFIP1	0.37	0.28	1.25	0.88	0.50	1.70E-05
1	IL2RG	0.63	0.56	3.34	2.50	0.42	1.78E-05
1	ATXN1	0.37	0.29	1.70	1.09	0.64	1.80E-05
1	PPDPF	0.48	0.40	2.10	1.47	0.52	2.13E-05

1	SPN	0.38	0.29	1.59	1.07	0.57	2.25E-05
1	OSTF1	0.43	0.35	1.85	1.27	0.54	2.25E-05
1	CCNDBP1	0.31	0.23	1.17	0.69	0.77	2.37E-05
1	ODC1	0.32	0.23	1.50	0.93	0.69	2.39E-05
1	HMGN2	0.51	0.44	2.46	1.78	0.47	2.89E-05
1	RNF168	0.42	0.33	1.75	1.24	0.50	3.02E-05
1	SETX	0.35	0.26	1.46	0.96	0.61	3.12E-05
1	CDC42SE2	0.70	0.67	4.73	3.62	0.39	3.14E-05
1	EBP	0.31	0.23	1.23	0.83	0.57	3.47E-05
1	SF3B2	0.45	0.38	1.82	1.27	0.53	3.72E-05
1	ORMDL1	0.40	0.32	1.60	1.09	0.55	3.73E-05
1	CIB1	0.50	0.42	2.22	1.62	0.46	3.79E-05
1	STAU1	0.38	0.30	1.41	0.99	0.51	3.80E-05
1	CAPZA1	0.51	0.43	2.12	1.59	0.41	3.85E-05
1	CCSER2	0.54	0.46	2.61	2.00	0.38	3.91E-05
1	ENSG00000	0.33	0.24	1.38	0.95	0.54	3.98E-05
1	TOMM20	0.55	0.47	2.39	1.82	0.39	4.24E-05
1	MECP2	0.44	0.37	2.04	1.33	0.61	4.40E-05
1	N4BP2L2	0.64	0.57	3.93	2.80	0.49	4.53E-05
1	CMPK1	0.29	0.21	1.04	0.64	0.69	4.55E-05
1	PSMB9	0.43	0.35	1.96	1.33	0.56	4.60E-05
1	DYNLT1	0.30	0.22	1.12	0.74	0.58	4.87E-05
1	PLAC8	0.38	0.29	1.77	1.23	0.53	5.09E-05
1	EIF4B	0.52	0.47	2.44	1.79	0.44	5.37E-05
1	ZNF292	0.40	0.31	1.84	1.27	0.53	5.60E-05
1	ERBIN	0.28	0.20	1.07	0.65	0.72	5.87E-05
1	HLA-DPA1	0.47	0.38	2.14	1.63	0.40	6.20E-05
1	LYST	0.56	0.50	4.02	2.75	0.55	7.48E-05
1	PRELID1	0.51	0.44	2.38	1.71	0.48	7.81E-05
1	RASA2	0.42	0.35	1.90	1.28	0.57	8.76E-05
1	UBE2R2	0.36	0.28	1.35	0.91	0.57	8.89E-05
1	TECR	0.32	0.24	1.22	0.83	0.56	9.16E-05
1	BRD7	0.38	0.31	1.69	1.14	0.57	9.27E-05
1	BTN3A2	0.37	0.29	1.53	1.03	0.56	9.80E-05
1	WDR1	0.31	0.23	1.18	0.73	0.68	9.90E-05
1	EPS15	0.27	0.20	0.95	0.57	0.74	0.00010693
1	IKZF1	0.55	0.49	2.82	2.13	0.41	0.00010742
1	CSGALNAC	0.30	0.23	1.09	0.70	0.64	0.00011168
1	PLAAT4	0.41	0.33	1.87	1.30	0.53	0.00011487
1	SPOCK2	0.39	0.33	2.48	1.45	0.78	0.00011618
1	MACF1	0.59	0.55	3.95	2.99	0.40	0.00011952
1	PSME1	0.59	0.51	2.93	2.27	0.37	0.00012012
1	ENSG00000	0.44	0.38	2.03	1.43	0.51	0.0001335
1	UTRN	0.49	0.43	2.69	1.87	0.53	0.00015015
1	SLTM	0.43	0.37	1.96	1.37	0.52	0.00015138
1	PRKACB	0.34	0.26	1.40	0.97	0.54	0.0001523
1	CSNK1G2	0.40	0.33	1.75	1.20	0.54	0.00015367
1	ADAR	0.40	0.32	1.60	1.17	0.45	0.00015435
1	GHITM	0.42	0.34	1.63	1.21	0.43	0.00015481
1	KLRC2	0.41	0.35	2.64	1.68	0.65	0.0001604
1	TMEM160	0.26	0.19	0.94	0.61	0.62	0.00016284
1	SMC3	0.39	0.32	1.70	1.20	0.51	0.00020393
1	RFC1	0.32	0.26	1.35	0.85	0.67	0.00023674
1	CCDC186	0.40	0.32	1.82	1.24	0.55	0.00023682
1	RABGAP1L	0.30	0.23	1.26	0.76	0.73	0.00027201
1	SPEN	0.43	0.37	2.19	1.49	0.56	0.00027289
1	LNPEP	0.37	0.31	1.71	1.15	0.57	0.00027292
1	STAT3	0.59	0.54	3.39	2.61	0.38	0.00027306
1	RIPOR2	0.36	0.30	1.97	1.22	0.70	0.00027686

1	EID1	0.33	0.26	1.28	0.88	0.54	0.00028027
1	MSL1	0.26	0.19	0.91	0.58	0.65	0.00029683
1	SRSF9	0.50	0.45	2.25	1.73	0.38	0.00031647
1	SRP9	0.31	0.25	1.16	0.78	0.58	0.00034183
1	TMED9	0.28	0.22	1.10	0.68	0.71	0.00036091
1	TXNRD1	0.32	0.25	1.45	0.97	0.58	0.00041471
1	CLTA	0.32	0.24	1.14	0.80	0.50	0.00041781
1	BPTF	0.45	0.40	2.31	1.72	0.42	0.0004487
1	MVD	0.29	0.22	1.03	0.75	0.46	0.00046199
1	LINC-PINT	0.59	0.54	3.51	2.73	0.36	0.00048982
1	ENSG00000	0.40	0.34	2.10	1.42	0.57	0.00050313
1	ARIH2	0.33	0.26	1.15	0.87	0.41	0.00053001
1	NSMCE3	0.32	0.25	1.16	0.86	0.44	0.00056076
1	GCC2	0.56	0.51	3.29	2.53	0.38	0.00058421
1	SH3BGRL	0.30	0.24	1.18	0.78	0.60	0.00060557
1	CRBN	0.30	0.23	1.21	0.76	0.66	0.00061465
1	RPS27L	0.41	0.35	1.88	1.31	0.52	0.00061544
1	KMT2A	0.46	0.40	2.05	1.60	0.36	0.00069906
1	EIF3A	0.52	0.47	2.51	1.95	0.37	0.00073418
1	NFIL3	0.32	0.25	1.42	1.02	0.48	0.00073516
1	PTPN7	0.37	0.30	1.53	1.09	0.49	0.00076054
1	THRAP3	0.45	0.39	1.93	1.50	0.36	0.00076458
1	LASP1	0.35	0.28	1.23	0.92	0.43	0.00079551
1	CYTH1	0.46	0.41	2.33	1.74	0.43	0.0008159
1	IGBP1	0.26	0.20	0.90	0.59	0.60	0.00081769
1	TP53BP2	0.25	0.19	0.95	0.58	0.73	0.00084223
1	RAC2	0.60	0.57	3.72	2.84	0.39	0.00084864
1	CREBRF	0.45	0.40	2.18	1.64	0.41	0.00091898
1	LPIN2	0.26	0.20	1.00	0.69	0.54	0.00092231
1	ARHGAP30	0.37	0.31	1.47	1.07	0.46	0.00096256
1	CD300A	0.45	0.38	2.42	1.82	0.41	0.00101143
1	FYB1	0.36	0.30	1.77	1.25	0.50	0.00105403
1	CDK2AP2	0.43	0.38	1.84	1.36	0.43	0.0010722
1	ATP2B1	0.39	0.33	1.82	1.37	0.40	0.00120002
1	CYRIB	0.40	0.33	1.59	1.21	0.40	0.001216
1	RAP1A	0.41	0.37	1.81	1.24	0.55	0.00122792
1	YPEL3	0.43	0.39	2.12	1.53	0.47	0.00123289
1	RNF149	0.38	0.34	1.75	1.24	0.50	0.0013228
1	FAM117A	0.25	0.19	0.86	0.62	0.48	0.00135771
1	SMARCA2	0.30	0.24	1.14	0.82	0.47	0.00137302
1	ZC3H13	0.25	0.19	1.01	0.67	0.59	0.00137707
1	PTPN1	0.26	0.20	1.03	0.61	0.76	0.00140424
1	TAOK3	0.35	0.28	1.39	1.07	0.38	0.00142403
1	HP1BP3	0.48	0.43	2.17	1.67	0.38	0.0014479
1	SPCS3	0.39	0.33	1.65	1.18	0.49	0.00149539
1	JADE2	0.27	0.21	1.02	0.68	0.59	0.00168434
1	ATP5MK	0.39	0.33	1.57	1.18	0.41	0.00177005
1	UBE2K	0.30	0.24	1.15	0.79	0.55	0.00180393
1	TIAL1	0.35	0.30	1.61	1.03	0.65	0.00183819
1	USP7	0.29	0.24	1.01	0.70	0.53	0.00191207
1	ARL8A	0.25	0.20	0.99	0.62	0.66	0.00195257
1	EPB41	0.29	0.23	1.05	0.77	0.44	0.00198978
1	NDUFB2	0.43	0.38	1.96	1.48	0.41	0.00202167
1	UQCRFS1	0.32	0.26	1.14	0.83	0.46	0.0022053
1	HERPUD2	0.29	0.23	1.15	0.80	0.53	0.00235153
1	XRN1	0.35	0.30	1.38	1.02	0.44	0.00235359
1	ATP1A1	0.42	0.37	1.95	1.48	0.40	0.00243385
1	CREBBP	0.30	0.25	1.19	0.78	0.62	0.00256062
1	ST8SIA4	0.27	0.21	1.06	0.77	0.46	0.00281847

1	XRN2	0.35	0.30	1.39	1.00	0.48	0.00287647
1	PHF20L1	0.37	0.31	1.53	1.13	0.44	0.00289084
1	TMEM258	0.35	0.29	1.37	1.03	0.41	0.0029457
1	ANXA6	0.29	0.24	1.10	0.73	0.60	0.00312489
1	TGOLN2	0.41	0.36	1.75	1.32	0.41	0.00334636
1	SPSB3	0.33	0.28	1.31	0.96	0.45	0.00344732
1	UQCR10	0.35	0.30	1.44	1.07	0.43	0.0036636
1	KIF2A	0.41	0.36	1.72	1.34	0.36	0.00391588
1	KIAA1109	0.32	0.28	1.37	0.97	0.49	0.00398201
1	RNF7	0.41	0.37	1.71	1.32	0.38	0.00405259
1	ARPC4	0.29	0.24	1.01	0.76	0.41	0.00448107
1	TRIM22	0.32	0.26	1.29	0.98	0.40	0.00454269
1	ATP6V0B	0.40	0.35	1.58	1.22	0.37	0.00459941
1	DCP2	0.29	0.24	1.12	0.78	0.53	0.00462152
1	FBXW7	0.27	0.22	1.09	0.69	0.65	0.00465303
1	DGKZ	0.37	0.32	1.52	1.15	0.41	0.00465955
1	SRPK1	0.28	0.23	1.12	0.74	0.61	0.00471112
1	BAX	0.26	0.21	0.98	0.67	0.54	0.00500572
1	P4HB	0.34	0.28	1.33	0.95	0.48	0.00515277
1	RAB14	0.37	0.32	1.43	1.08	0.40	0.00519948
1	ARPC1B	0.37	0.32	1.56	1.13	0.47	0.00547615
1	RAB18	0.29	0.24	1.04	0.74	0.49	0.00582041
1	RNF115	0.38	0.34	1.67	1.20	0.47	0.00584525
1	ADNP	0.26	0.21	0.84	0.63	0.41	0.00612887
1	MRPS34	0.26	0.21	0.93	0.65	0.52	0.0061957
1	PTBP3	0.32	0.27	1.20	0.91	0.40	0.00626365
1	ATP5IF1	0.38	0.34	1.69	1.29	0.38	0.00671242
1	GABARAPL	0.39	0.35	1.57	1.14	0.47	0.00726552
1	ISCA1	0.40	0.35	1.54	1.18	0.38	0.00733717
1	RAB10	0.30	0.25	1.07	0.81	0.39	0.00733989
1	ADD1	0.28	0.23	1.12	0.76	0.56	0.00774527
1	ZBTB7A	0.37	0.32	1.57	1.15	0.45	0.00812841
1	CAP1	0.44	0.40	2.03	1.56	0.38	0.00822662
1	RBM38	0.36	0.31	1.49	1.15	0.37	0.00832976
1	NDUFA12	0.32	0.27	1.22	0.90	0.43	0.00856461
1	CDK13	0.33	0.28	1.17	0.90	0.38	0.00895537
1	FRG1	0.27	0.22	0.91	0.69	0.40	0.00912962
1	POLR1D	0.42	0.38	1.67	1.30	0.37	0.0092056
1	YAF2	0.26	0.22	1.03	0.74	0.48	0.00980904
1	KDELR2	0.25	0.21	0.88	0.61	0.52	0.0098433
1	PSMB6	0.36	0.33	1.50	1.10	0.45	0.01010445
1	COX14	0.32	0.27	1.14	0.85	0.42	0.0105363
1	TMOD3	0.35	0.30	1.28	0.97	0.40	0.01058846
1	KCTD20	0.26	0.21	0.95	0.68	0.49	0.01064416
1	TBCA	0.34	0.30	1.40	1.05	0.40	0.01098646
1	TRPM7	0.26	0.21	0.89	0.65	0.46	0.01119904
1	CAPZB	0.37	0.33	1.49	1.11	0.42	0.01123709
1	SNRNP200	0.29	0.24	1.11	0.78	0.52	0.01151479
1	SYF2	0.53	0.52	2.78	2.14	0.38	0.01182973
1	EIF2S3	0.37	0.34	1.52	1.15	0.40	0.0120087
1	PSMB3	0.27	0.23	1.01	0.69	0.56	0.01223052
1	TNFAIP8	0.26	0.22	1.16	0.86	0.44	0.01253144
1	EIF3L	0.28	0.24	0.99	0.73	0.45	0.0125859
1	ATP2B4	0.25	0.21	1.06	0.74	0.53	0.01343483
1	MDM4	0.30	0.26	1.24	0.93	0.42	0.01352462
1	PDCD7	0.26	0.22	0.97	0.69	0.49	0.01360785
1	SSH2	0.26	0.22	1.06	0.72	0.55	0.01408768
1	ERP44	0.26	0.22	0.85	0.64	0.41	0.01418818
1	NDUFA1	0.43	0.39	1.84	1.41	0.39	0.01471309

1	MED10	0.34	0.30	1.34	0.98	0.46	0.01474221
1	UHMK1	0.26	0.21	0.95	0.69	0.46	0.016979
1	SAFB	0.28	0.24	1.05	0.79	0.40	0.01729355
1	AURKAIP1	0.37	0.33	1.37	1.07	0.36	0.01772174
1	HSH2D	0.28	0.23	1.21	0.92	0.40	0.01815482
1	CXCL8	0.26	0.22	1.23	0.86	0.52	0.01947126
1	SERPINB1	0.36	0.32	1.74	1.34	0.38	0.02110081
1	PPP1CA	0.27	0.23	0.97	0.74	0.39	0.02140138
1	CSDE1	0.48	0.45	2.11	1.62	0.38	0.02170729
1	RAB29	0.30	0.25	1.21	0.89	0.44	0.02253878
1	MAP2K2	0.36	0.33	1.39	1.05	0.40	0.02319055
1	CHD3	0.33	0.30	1.63	1.08	0.60	0.02320679
1	PRDX5	0.31	0.27	1.28	0.93	0.45	0.02656727
1	TRBC2	0.41	0.39	2.07	1.56	0.41	0.03058906
1	ANXA5	0.25	0.21	1.06	0.78	0.44	0.03075889
1	MAT2B	0.26	0.23	0.97	0.74	0.40	0.03274525
1	ESYT2	0.26	0.23	1.08	0.73	0.57	0.03375164
1	GTF3A	0.35	0.31	1.45	1.11	0.39	0.03444681
1	HNRNPL	0.33	0.30	1.28	0.97	0.41	0.03489004
1	SAMD9	0.26	0.22	1.00	0.78	0.36	0.04058665
1	BCL9L	0.26	0.24	1.14	0.82	0.47	0.04159875
1	DR1	0.32	0.28	1.20	0.94	0.36	0.04387401
1	STARD3NL	0.29	0.25	1.06	0.82	0.37	0.04523085
1	GBP4	0.28	0.25	1.26	0.98	0.36	0.04970632
2	DNAJB1	0.96	0.59	70.06	15.30	2.20	4.01E-109
2	HSPA1B	0.92	0.49	68.65	13.27	2.37	5.93E-107
2	NR4A1	0.77	0.28	12.48	1.80	2.79	5.76E-103
2	PPP1R15A	0.93	0.61	22.15	5.56	1.99	1.92E-90
2	HSPA1A	0.98	0.80	200.73	56.46	1.83	6.09E-90
2	FOSB	0.64	0.26	7.40	1.32	2.48	3.71E-62
2	FOS	0.81	0.48	33.01	6.55	2.33	1.30E-61
2	SERPINH1	0.45	0.12	4.02	0.65	2.62	9.97E-61
2	HSPA6	0.66	0.31	42.36	8.63	2.29	1.20E-58
2	HSPH1	0.92	0.68	34.93	12.34	1.50	1.39E-58
2	HSP90AA1	0.99	0.96	240.15	107.52	1.16	1.92E-57
2	JUN	0.82	0.50	21.59	6.25	1.79	7.12E-57
2	CD69	0.93	0.76	25.71	9.24	1.48	2.54E-55
2	JUNB	0.90	0.73	24.28	7.31	1.73	8.81E-51
2	ZFP36	0.83	0.63	16.45	4.93	1.74	7.45E-49
2	DUSP1	0.80	0.58	23.66	5.55	2.09	7.99E-49
2	BAG3	0.41	0.12	2.74	0.46	2.56	2.00E-48
2	DNAJA1	0.88	0.78	18.12	7.60	1.25	4.67E-44
2	CLK1	0.86	0.64	9.71	3.90	1.32	1.14E-43
2	HSPA8	0.95	0.89	37.40	17.45	1.10	5.49E-43
2	ZFAND2A	0.56	0.27	5.33	1.27	2.07	3.59E-40
2	EGR1	0.27	0.06	1.57	0.22	2.86	2.20E-39
2	UBC	0.97	0.93	44.48	20.89	1.09	2.93E-38
2	RGCC	0.61	0.32	6.48	1.66	1.96	3.95E-38
2	DEDD2	0.54	0.26	3.06	0.98	1.64	2.17E-37
2	RGS1	0.58	0.32	8.91	1.89	2.24	7.00E-36
2	KLF6	0.88	0.72	15.80	7.11	1.15	8.71E-35
2	HSPE1	0.94	0.83	34.07	18.00	0.92	4.07E-34
2	HSPB1	0.75	0.52	13.40	4.84	1.47	8.48E-33
2	CACYBP	0.76	0.57	10.58	4.52	1.23	1.57E-32
2	DNAJB4	0.44	0.19	2.86	0.87	1.72	7.84E-30
2	DUSP2	0.92	0.88	32.83	14.81	1.15	5.77E-29
2	ATF3	0.37	0.15	2.69	0.69	1.97	6.33E-29
2	JUND	0.99	0.97	52.58	33.59	0.65	7.39E-27
2	IER5	0.68	0.49	8.63	2.78	1.64	9.17E-27

2	HSP90AB1	0.95	0.95	72.05	41.18	0.81	6.35E-26
2	SLC2A3	0.68	0.48	6.84	2.78	1.30	9.13E-26
2	TRA2B	0.74	0.57	6.60	2.79	1.24	1.21E-25
2	TNFSF14	0.57	0.35	4.98	1.60	1.64	2.04E-25
2	AREG	0.59	0.35	10.20	3.92	1.38	4.53E-25
2	NFKBIA	0.90	0.81	27.42	12.43	1.14	4.77E-25
2	UBB	0.94	0.85	23.03	12.05	0.93	5.06E-25
2	HSPD1	0.93	0.88	58.44	29.11	1.01	1.86E-24
2	RHOB	0.30	0.11	1.54	0.47	1.72	2.53E-24
2	MCL1	0.85	0.79	13.31	6.91	0.95	2.04E-23
2	CCNL1	0.77	0.64	6.70	3.46	0.95	2.18E-23
2	UBE2S	0.62	0.44	6.01	2.11	1.51	2.70E-23
2	BTG2	0.70	0.52	6.82	2.87	1.25	3.96E-23
2	SPRY2	0.32	0.13	1.41	0.44	1.70	1.09E-22
2	ZC3H12A	0.53	0.32	3.24	1.21	1.42	1.94E-22
2	IER5L	0.36	0.17	3.39	0.75	2.19	1.30E-21
2	TENT5A	0.36	0.17	1.88	0.56	1.74	1.61E-21
2	NKRF	0.27	0.10	1.11	0.30	1.87	2.32E-21
2	GADD45B	0.76	0.62	20.37	6.92	1.56	3.04E-21
2	SPRY1	0.37	0.17	2.99	0.83	1.84	4.64E-21
2	GADD45G	0.29	0.12	2.73	0.54	2.35	6.22E-20
2	SRSF3	0.79	0.67	7.70	3.90	0.98	7.99E-20
2	TSEN34	0.33	0.15	1.54	0.50	1.62	1.07E-18
2	ARL5B	0.37	0.19	2.34	0.68	1.78	1.27E-18
2	RASGEF1B	0.36	0.19	2.06	0.68	1.61	2.40E-17
2	NEU1	0.42	0.24	2.73	0.95	1.53	7.38E-17
2	YPEL5	0.66	0.53	5.13	2.35	1.13	1.24E-16
2	IER2	0.79	0.74	13.92	6.09	1.19	1.26E-16
2	NR4A2	0.71	0.55	8.05	3.92	1.04	1.81E-16
2	MRPL18	0.47	0.28	2.88	1.20	1.27	2.00E-16
2	ZBTB10	0.39	0.22	2.11	0.85	1.30	2.28E-16
2	H3-3B	0.98	0.98	33.71	21.48	0.65	2.91E-16
2	MYLIP	0.34	0.17	1.81	0.65	1.47	5.52E-16
2	ANKRD37	0.30	0.14	1.88	0.55	1.77	7.24E-16
2	MAFF	0.43	0.26	2.40	0.98	1.29	1.97E-15
2	TXNIP	0.69	0.58	9.70	4.66	1.06	4.72E-15
2	CHASERR	0.75	0.63	5.85	3.41	0.78	5.08E-15
2	CCT4	0.66	0.52	4.99	2.64	0.92	6.90E-15
2	CKS2	0.46	0.28	2.45	1.19	1.04	7.01E-15
2	RGS2	0.45	0.28	3.42	1.32	1.37	1.71E-14
2	LMNA	0.71	0.60	10.89	5.13	1.09	1.78E-14
2	BTG1-DT	0.34	0.18	2.07	0.73	1.50	2.65E-14
2	TAGAP	0.64	0.54	5.90	2.86	1.05	2.35E-13
2	DNAJB6	0.79	0.73	9.14	5.60	0.71	3.62E-13
2	BCAS2	0.48	0.32	2.89	1.25	1.21	4.04E-13
2	ENSG0000(	0.43	0.27	2.36	1.06	1.16	5.08E-13
2	HNRNPU	0.86	0.79	10.14	6.64	0.61	9.36E-13
2	XCL1	0.77	0.56	28.98	17.77	0.71	1.13E-12
2	TCP1	0.60	0.48	3.75	2.12	0.82	2.48E-12
2	EIF4A2	0.80	0.74	7.17	4.79	0.58	4.05E-12
2	CSRNP1	0.44	0.29	2.11	0.98	1.11	4.52E-12
2	ZC3HAV1	0.66	0.53	5.32	3.12	0.77	1.10E-11
2	CHORDC1	0.59	0.50	6.44	3.52	0.87	4.54E-11
2	IFRD1	0.42	0.28	2.27	0.98	1.21	5.68E-11
2	MYADM	0.40	0.27	2.38	0.97	1.30	6.69E-11
2	ZFP36L1	0.67	0.60	8.76	4.15	1.08	2.62E-10
2	CHD2	0.70	0.58	5.28	3.35	0.66	3.00E-10
2	SNHG8	0.66	0.57	4.97	2.97	0.74	3.72E-10
2	MIR23AHG	0.54	0.40	5.14	2.96	0.80	3.85E-10

2	PHLDA1	0.41	0.28	2.55	1.13	1.17	4.36E-10
2	TSPYL2	0.64	0.56	8.39	4.25	0.98	5.48E-10
2	CD83	0.35	0.22	2.64	1.26	1.07	7.35E-10
2	TAF7	0.64	0.53	3.91	2.45	0.67	7.98E-10
2	INTS6	0.42	0.29	2.31	1.16	0.99	1.09E-09
2	DDX3X	0.77	0.71	7.83	4.84	0.69	1.28E-09
2	GPBP1	0.85	0.82	9.44	6.91	0.45	1.44E-09
2	SERTAD1	0.39	0.27	2.77	1.04	1.41	1.64E-09
2	NFKBID	0.40	0.26	2.19	1.15	0.92	2.75E-09
2	AHSA1	0.59	0.47	3.41	2.25	0.60	2.76E-09
2	PPP1R15B	0.49	0.38	2.72	1.43	0.92	2.98E-09
2	PPP1R2	0.61	0.52	5.17	2.58	1.00	3.54E-09
2	MXD1	0.37	0.25	1.79	0.93	0.93	3.57E-09
2	CITED2	0.34	0.21	1.99	0.91	1.13	4.14E-09
2	SFPQ	0.73	0.65	5.18	3.51	0.56	8.68E-09
2	H2AX	0.37	0.25	1.75	0.87	1.01	1.34E-08
2	PPP1R10	0.41	0.30	2.71	1.18	1.19	1.45E-08
2	TUBA1A	0.56	0.48	4.94	2.27	1.12	1.85E-08
2	DYNLL1	0.64	0.56	6.36	3.29	0.95	2.75E-08
2	GATA3	0.35	0.24	1.66	0.83	1.00	2.98E-08
2	RAB11FIP1	0.50	0.39	2.70	1.64	0.72	4.11E-08
2	DOK2	0.61	0.51	4.62	2.95	0.65	5.10E-08
2	NABP1	0.34	0.23	2.01	0.83	1.27	6.37E-08
2	CGAS	0.29	0.18	1.22	0.68	0.85	6.86E-08
2	HMGCS1	0.27	0.16	1.32	0.57	1.20	8.56E-08
2	PDE4B	0.57	0.44	3.65	2.26	0.69	9.00E-08
2	PRMT9	0.26	0.16	1.26	0.55	1.20	1.26E-07
2	ABHD3	0.36	0.24	1.45	0.88	0.72	1.53E-07
2	MIDEAS	0.31	0.20	1.58	0.74	1.10	2.36E-07
2	EIF4A3	0.37	0.28	2.11	0.93	1.17	4.07E-07
2	ATP1B1	0.38	0.26	2.15	1.32	0.70	4.19E-07
2	HEXIM1	0.35	0.25	1.77	0.90	0.97	4.42E-07
2	ZNF331	0.60	0.51	5.76	3.54	0.70	4.77E-07
2	PMAIP1	0.45	0.35	3.41	2.05	0.73	4.92E-07
2	AHR	0.34	0.23	1.66	0.85	0.97	5.44E-07
2	AZIN1	0.39	0.28	1.58	0.94	0.74	5.98E-07
2	CYCS	0.61	0.53	4.14	2.61	0.66	9.60E-07
2	AMD1	0.57	0.48	3.54	2.21	0.68	1.05E-06
2	HSPA9	0.56	0.47	3.07	1.85	0.73	1.12E-06
2	DUSP10	0.29	0.19	1.39	0.62	1.15	1.24E-06
2	TSC22D3	0.72	0.66	10.35	6.59	0.65	1.47E-06
2	TRA2A	0.58	0.49	3.34	2.12	0.65	2.15E-06
2	SBDS	0.41	0.30	1.77	1.08	0.71	2.53E-06
2	EIF5	0.68	0.66	5.55	3.65	0.60	2.77E-06
2	PIK3IP1	0.27	0.17	1.01	0.56	0.86	4.15E-06
2	FUS	0.69	0.65	6.32	3.78	0.74	4.18E-06
2	NOP58	0.45	0.36	2.26	1.32	0.77	5.56E-06
2	MOB4	0.49	0.42	3.16	1.77	0.84	5.66E-06
2	DDIT4	0.61	0.56	6.61	3.65	0.86	7.57E-06
2	ZNF394	0.35	0.26	1.32	0.78	0.75	8.00E-06
2	SLC38A2	0.63	0.58	4.70	3.15	0.58	1.21E-05
2	REX1BD	0.26	0.17	0.90	0.57	0.66	1.32E-05
2	MIDN	0.47	0.39	2.67	1.58	0.76	1.69E-05
2	THAP9-AS1	0.32	0.23	1.37	0.76	0.85	1.79E-05
2	NFKBIZ	0.50	0.40	3.17	2.16	0.55	1.86E-05
2	GADD45A	0.35	0.27	2.80	1.20	1.22	2.01E-05
2	IER3	0.31	0.22	2.72	1.02	1.42	2.11E-05
2	NUDT4	0.27	0.18	1.11	0.61	0.86	3.40E-05
2	JMJD6	0.44	0.37	2.34	1.36	0.79	3.41E-05

2	TNFAIP3	0.80	0.78	12.55	8.01	0.65	3.76E-05
2	PNPLA8	0.41	0.32	1.82	1.14	0.68	3.98E-05
2	DUSP4	0.39	0.30	2.89	1.67	0.79	4.28E-05
2	KAT6B	0.30	0.21	1.12	0.72	0.65	4.31E-05
2	IRF1	0.71	0.66	6.23	4.53	0.46	4.72E-05
2	FKBP4	0.54	0.46	4.77	3.16	0.59	5.59E-05
2	BRD2	0.68	0.67	6.95	3.87	0.85	5.78E-05
2	PRR7	0.32	0.24	1.33	0.81	0.72	6.70E-05
2	ERN1	0.37	0.28	2.10	1.48	0.51	7.18E-05
2	NUFIP2	0.54	0.48	3.17	2.14	0.57	8.02E-05
2	HES4	0.25	0.17	2.09	0.92	1.18	8.22E-05
2	COTL1	0.48	0.39	2.99	2.21	0.44	8.37E-05
2	ARL4A	0.30	0.22	1.54	0.83	0.89	8.74E-05
2	CDKN1A	0.33	0.25	1.64	0.99	0.72	0.00012032
2	MAP3K8	0.51	0.44	2.96	2.09	0.50	0.00012178
2	NAMPT	0.60	0.53	5.47	3.43	0.67	0.0001579
2	CHMP1B	0.35	0.28	1.79	1.01	0.82	0.00016684
2	KLHL6	0.30	0.22	1.48	0.89	0.73	0.00017163
2	ACAP1	0.53	0.46	2.85	1.98	0.52	0.00034697
2	ENSG00000	0.27	0.20	1.16	0.80	0.54	0.00035819
2	XCL2	0.79	0.70	33.20	25.26	0.39	0.0003693
2	IFNG	0.44	0.37	10.86	6.25	0.80	0.0004254
2	SOCS1	0.34	0.27	1.75	1.07	0.71	0.00042889
2	SIAH2	0.30	0.23	1.15	0.74	0.64	0.00044051
2	EVL	0.40	0.32	1.86	1.38	0.44	0.00063532
2	BUD31	0.48	0.41	2.27	1.54	0.56	0.00078899
2	TOPORS	0.30	0.23	1.20	0.76	0.66	0.00079162
2	YTHDC1	0.49	0.46	2.92	1.96	0.57	0.00093325
2	ARRDC3	0.40	0.33	1.97	1.53	0.37	0.00099543
2	PER1	0.32	0.26	1.46	0.87	0.75	0.00121235
2	B3GNT2	0.35	0.29	1.58	0.95	0.73	0.00139369
2	NBEAL1	0.26	0.19	0.91	0.62	0.57	0.00158119
2	LINC01138	0.28	0.21	1.30	0.79	0.72	0.00203782
2	FYB1	0.36	0.30	1.90	1.24	0.62	0.00214257
2	TOB1	0.27	0.20	1.27	0.88	0.52	0.00243773
2	PIM3	0.55	0.52	3.63	2.58	0.49	0.00294377
2	CDKN1B	0.51	0.47	2.84	2.01	0.50	0.00304296
2	TAMALIN	0.34	0.28	2.05	1.33	0.62	0.00323512
2	NXT1	0.27	0.21	0.88	0.64	0.47	0.00325762
2	TSC22D2	0.25	0.19	0.97	0.64	0.60	0.00327752
2	COPA	0.53	0.50	3.35	2.32	0.53	0.00382784
2	AFF4	0.35	0.29	1.50	1.01	0.57	0.00395277
2	DDIT3	0.27	0.21	1.21	0.78	0.63	0.00504361
2	TIPARP	0.46	0.41	2.66	1.78	0.58	0.0064962
2	LUZP1	0.36	0.32	1.78	1.17	0.61	0.00709554
2	BHLHE40	0.49	0.45	2.96	2.18	0.44	0.00710718
2	RBBP6	0.36	0.30	1.45	1.05	0.46	0.00727427
2	GPX1	0.34	0.28	1.50	0.95	0.66	0.0081507
2	SPTY2D1	0.40	0.35	2.00	1.31	0.61	0.00900135
2	CEBPD	0.34	0.29	2.87	1.70	0.75	0.00994878
2	BEX4	0.25	0.20	0.94	0.64	0.54	0.01033171
2	ENSG00000	0.32	0.26	1.32	0.95	0.48	0.0106645
2	SAT1	0.82	0.84	14.37	9.87	0.54	0.01364193
2	KDM2A	0.41	0.36	1.68	1.23	0.44	0.01525502
2	EIF4A1	0.27	0.22	1.04	0.68	0.62	0.01533483
2	RPL22L1	0.47	0.43	2.47	1.89	0.38	0.01613334
2	RSRC2	0.59	0.61	4.16	3.06	0.44	0.01756043
2	ZFAND5	0.41	0.39	2.20	1.46	0.59	0.020316
2	VMP1	0.44	0.38	2.30	1.75	0.40	0.02067312

2	HECA	0.39	0.35	1.74	1.28	0.45	0.02105793
2	PAF1	0.25	0.20	0.90	0.63	0.53	0.02113271
2	CCDC59	0.32	0.26	1.22	0.91	0.42	0.02238155
2	SRSF7	0.78	0.81	9.33	6.81	0.46	0.02322384
2	CDKN2D	0.41	0.36	2.11	1.46	0.54	0.02327651
2	STIP1	0.46	0.43	2.36	1.77	0.42	0.02374072
2	BCL2	0.29	0.24	1.35	0.88	0.62	0.0242222
2	TRMT10C	0.28	0.24	1.02	0.75	0.43	0.02606188
2	ZNF326	0.25	0.21	0.91	0.68	0.42	0.02741796
2	HERPUD1	0.47	0.44	2.83	2.00	0.50	0.03448755
2	GLA	0.35	0.31	2.04	1.33	0.62	0.03600398
2	SLC39A10	0.31	0.27	1.37	1.06	0.37	0.04173611
2	NECAP2	0.31	0.27	1.16	0.88	0.40	0.04469873
3	DNAJB1	0.93	0.60	57.37	18.90	1.60	1.43E-78
3	HSPA1B	0.87	0.51	49.96	17.98	1.47	1.81E-66
3	FGFBP2	0.61	0.18	5.27	1.50	1.81	3.48E-65
3	HSPA1A	0.95	0.81	171.38	65.19	1.39	2.55E-60
3	CCL4L2	0.59	0.21	20.80	4.42	2.23	4.72E-59
3	CACYBP	0.86	0.56	11.40	4.51	1.34	7.62E-56
3	UBC	0.99	0.93	47.91	20.81	1.20	1.79E-54
3	IFNG	0.69	0.33	21.19	4.47	2.24	1.39E-53
3	CCL4	0.87	0.49	51.37	17.95	1.52	5.89E-53
3	HSPA8	0.97	0.89	38.86	17.65	1.14	7.81E-53
3	HSP90AA1	0.99	0.96	251.67	108.50	1.21	3.42E-52
3	GZMH	0.73	0.32	6.39	2.51	1.35	1.10E-48
3	DNAJA1	0.93	0.77	17.48	7.96	1.13	3.06E-48
3	HSPA6	0.66	0.32	36.47	10.50	1.80	3.26E-47
3	FCGR3A	0.67	0.29	4.82	1.91	1.34	4.05E-46
3	UBB	0.96	0.85	23.84	12.16	0.97	1.25E-43
3	NKG7	0.99	0.91	38.21	20.55	0.89	2.70E-43
3	CCL3	0.64	0.29	19.24	5.56	1.79	1.75E-41
3	SPON2	0.38	0.11	2.28	0.58	1.99	1.11E-38
3	JUN	0.80	0.51	17.49	7.35	1.25	1.04E-37
3	ACTB	0.94	0.84	20.77	9.60	1.11	1.29E-35
3	UBE2S	0.73	0.43	5.01	2.38	1.07	5.85E-35
3	ENSG00000	0.41	0.14	2.50	0.67	1.90	8.43E-35
3	CCL5	0.97	0.79	32.40	19.67	0.72	9.49E-35
3	S100A4	0.84	0.61	10.06	4.86	1.05	1.04E-32
3	HSPH1	0.87	0.69	27.92	14.15	0.98	2.11E-32
3	TXNIP	0.81	0.56	10.42	4.65	1.16	3.73E-32
3	DEDD2	0.56	0.26	2.67	1.10	1.28	3.80E-31
3	TNF	0.33	0.11	2.21	0.55	2.00	3.14E-29
3	SYNE1	0.52	0.24	2.55	1.06	1.27	3.60E-29
3	GZMA	0.71	0.41	8.59	5.29	0.70	7.09E-29
3	PRDM1	0.51	0.23	2.93	1.14	1.36	1.03E-28
3	CTSC	0.67	0.40	4.53	2.07	1.13	1.95E-27
3	BAG3	0.37	0.13	1.64	0.72	1.19	3.80E-27
3	LDLR	0.34	0.12	1.46	0.47	1.64	2.56E-26
3	APOL6	0.54	0.27	2.31	1.03	1.16	3.23E-25
3	SYNE2	0.69	0.39	4.44	2.62	0.76	7.99E-25
3	RGS2	0.53	0.27	3.08	1.43	1.10	1.89E-24
3	ENSG00000	0.28	0.09	1.13	0.45	1.33	2.11E-24
3	KLF2	0.65	0.38	6.40	2.83	1.18	2.47E-24
3	CGAS	0.38	0.16	1.85	0.58	1.69	6.91E-24
3	PPP1R15A	0.83	0.64	13.86	7.46	0.89	7.79E-24
3	ZFAND2A	0.53	0.28	3.51	1.70	1.05	8.52E-24
3	HSPE1	0.92	0.84	32.37	18.69	0.79	1.52E-23
3	C12orf75	0.49	0.24	2.19	1.00	1.13	9.91E-23
3	PMAIP1	0.56	0.33	4.94	1.80	1.46	2.05E-22

3	TMSB10	0.99	0.96	29.84	20.53	0.54	8.51E-22
3	HCST	0.86	0.70	8.59	4.94	0.80	8.63E-22
3	MRPL18	0.50	0.28	2.81	1.25	1.16	4.76E-21
3	HSP90AB1	0.99	0.95	69.88	42.30	0.72	1.34E-20
3	TOB1	0.39	0.18	2.12	0.73	1.53	1.97E-20
3	GZMM	0.66	0.41	3.59	2.04	0.81	2.12E-20
3	BIN2	0.51	0.28	2.07	1.05	0.98	6.05E-20
3	UAP1	0.33	0.14	1.28	0.47	1.45	7.36E-20
3	DNAJB6	0.87	0.72	8.97	5.72	0.65	2.08E-19
3	CCL3L1	0.28	0.10	2.20	0.90	1.29	4.06E-19
3	DOK2	0.70	0.50	5.36	2.85	0.91	4.89E-19
3	PCED1B-AS	0.54	0.31	2.37	1.17	1.01	1.06E-18
3	ITGB2	0.56	0.33	2.99	1.48	1.01	1.08E-18
3	ATF3	0.35	0.15	2.09	0.84	1.31	1.67E-18
3	SH3BGRL3	0.88	0.74	8.61	5.48	0.65	2.49E-18
3	NEU1	0.45	0.24	2.35	1.06	1.15	6.12E-18
3	IRF1	0.81	0.64	7.31	4.38	0.74	8.17E-18
3	HSPB1	0.75	0.53	9.14	5.82	0.65	1.14E-17
3	SLC20A1	0.42	0.22	2.03	0.77	1.40	1.30E-17
3	CALM1	0.96	0.88	15.25	11.00	0.47	2.96E-17
3	PFN1	0.93	0.83	11.52	7.35	0.65	3.96E-17
3	CKS2	0.48	0.28	2.81	1.15	1.28	7.28E-17
3	CDC42EP3	0.46	0.26	2.27	1.01	1.16	1.20E-16
3	H3-3B	0.99	0.97	30.79	22.30	0.47	1.60E-16
3	ADGRG1	0.28	0.11	0.94	0.44	1.12	5.18E-16
3	PYHIN1	0.45	0.23	2.05	1.13	0.86	6.13E-16
3	DNAJB4	0.40	0.21	2.23	1.03	1.12	6.45E-16
3	CD69	0.89	0.77	17.86	11.06	0.69	1.20E-15
3	PLEK	0.62	0.41	4.03	2.40	0.75	5.09E-15
3	LYAR	0.37	0.19	1.50	0.71	1.08	6.58E-15
3	ERN1	0.46	0.27	3.07	1.31	1.22	1.10E-14
3	GBP5	0.46	0.26	2.24	1.28	0.81	2.55E-14
3	RNF213	0.71	0.53	4.71	3.11	0.60	3.83E-14
3	SERPINH1	0.32	0.15	2.23	1.06	1.08	5.02E-14
3	IER5L	0.35	0.17	1.88	1.08	0.79	5.03E-14
3	KLF6	0.84	0.73	12.92	7.84	0.72	5.48E-14
3	ZC3HAV1	0.70	0.53	5.21	3.19	0.71	5.84E-14
3	AHSA1	0.64	0.46	3.88	2.19	0.83	7.78E-14
3	IDI1	0.58	0.42	3.26	1.74	0.90	2.29E-13
3	JUNB	0.83	0.75	14.15	9.56	0.57	2.36E-13
3	ANXA1	0.83	0.70	17.27	10.17	0.76	2.58E-13
3	CHORDC1	0.64	0.49	6.55	3.57	0.88	2.80E-13
3	ENC1	0.25	0.11	1.26	0.46	1.46	4.62E-13
3	GADD45B	0.76	0.62	13.75	8.44	0.70	6.58E-13
3	GNG2	0.66	0.44	4.43	2.97	0.58	6.74E-13
3	CYBA	0.90	0.75	9.68	6.75	0.52	9.35E-13
3	KMT2E-AS1	0.30	0.14	1.19	0.61	0.96	1.65E-12
3	ADRB2	0.30	0.15	1.20	0.59	1.03	1.73E-12
3	BCL11B	0.37	0.19	1.50	0.80	0.90	1.87E-12
3	DYNLL1	0.72	0.55	5.29	3.56	0.57	2.71E-12
3	SRSF3	0.81	0.67	6.07	4.29	0.50	3.75E-12
3	NEDD9	0.28	0.13	1.06	0.50	1.10	4.57E-12
3	RIPK2	0.26	0.12	0.86	0.40	1.13	4.69E-12
3	MYLIP	0.33	0.18	1.61	0.72	1.17	5.94E-12
3	MYBL1	0.45	0.26	2.06	1.20	0.77	6.54E-12
3	AMD1	0.62	0.47	3.89	2.18	0.84	7.54E-12
3	TLE5	0.65	0.48	3.23	2.05	0.66	8.49E-12
3	NCR3	0.25	0.12	0.94	0.39	1.27	1.09E-11
3	MXD1	0.42	0.24	1.60	0.99	0.69	1.74E-11

3	ARPC5L	0.64	0.48	3.24	2.05	0.66	1.93E-11
3	GNPTAB	0.41	0.24	1.86	1.08	0.78	2.12E-11
3	ARRDC3	0.48	0.31	2.83	1.38	1.04	2.36E-11
3	CCT4	0.68	0.52	4.35	2.81	0.63	3.04E-11
3	TBC1D10C	0.31	0.15	1.08	0.61	0.83	3.08E-11
3	REX1BD	0.31	0.16	1.12	0.54	1.05	3.72E-11
3	TRBC1	0.55	0.38	3.24	1.88	0.79	3.75E-11
3	CLK1	0.80	0.66	6.52	4.62	0.50	4.53E-11
3	PPP1R18	0.46	0.31	2.03	1.09	0.90	5.77E-11
3	TCP1	0.62	0.48	3.44	2.21	0.64	5.95E-11
3	DNAJA4	0.29	0.16	1.73	0.72	1.26	7.78E-11
3	NR4A2	0.69	0.55	6.53	4.29	0.61	1.13E-10
3	SBDS	0.46	0.30	1.90	1.07	0.82	1.29E-10
3	S100A6	0.83	0.67	7.38	5.70	0.37	1.77E-10
3	ACTG1	0.93	0.87	14.80	10.85	0.45	2.81E-10
3	DDIT3	0.34	0.20	1.51	0.74	1.04	3.94E-10
3	TRBC2	0.54	0.36	2.36	1.53	0.63	4.33E-10
3	AZIN1	0.44	0.28	1.64	0.95	0.80	4.44E-10
3	DUSP5	0.44	0.29	2.29	1.32	0.80	5.31E-10
3	CYCS	0.67	0.52	3.97	2.68	0.57	5.90E-10
3	MAF	0.29	0.15	1.20	0.72	0.74	6.00E-10
3	CYTOR	0.47	0.31	2.01	1.27	0.66	7.45E-10
3	ITGAL	0.42	0.27	1.74	1.05	0.73	7.49E-10
3	EFHD2	0.71	0.57	4.89	3.32	0.56	8.13E-10
3	GP6-AS1	0.27	0.14	0.99	0.47	1.06	8.64E-10
3	HSPD1	0.92	0.88	44.18	32.41	0.45	1.06E-09
3	MYL12A	0.75	0.67	7.47	4.49	0.73	1.44E-09
3	UPP1	0.44	0.29	1.92	1.12	0.77	1.48E-09
3	TRA2B	0.71	0.58	4.41	3.27	0.43	2.14E-09
3	UCP2	0.30	0.17	1.04	0.55	0.91	2.43E-09
3	CEP78	0.28	0.15	1.08	0.59	0.86	2.96E-09
3	NFIL3	0.38	0.24	1.90	0.96	0.99	2.99E-09
3	LGALS1	0.81	0.66	8.99	6.74	0.42	3.47E-09
3	CFL1	0.91	0.83	9.71	7.43	0.39	4.40E-09
3	CCND3	0.39	0.24	1.49	0.85	0.81	4.73E-09
3	PTPN4	0.30	0.17	1.05	0.60	0.81	5.02E-09
3	TNFSF9	0.26	0.14	1.20	0.68	0.81	5.68E-09
3	CARD16	0.38	0.23	1.61	0.94	0.77	6.27E-09
3	PPP2R5C	0.72	0.56	4.80	3.52	0.45	8.16E-09
3	VMP1	0.51	0.37	2.78	1.67	0.73	9.03E-09
3	EIF5	0.77	0.64	5.06	3.78	0.42	1.42E-08
3	KLRF1	0.45	0.31	2.17	1.42	0.62	1.48E-08
3	IL32	0.71	0.55	7.90	5.78	0.45	2.08E-08
3	PLAAT4	0.48	0.32	1.90	1.32	0.52	2.35E-08
3	CAPN2	0.25	0.14	0.87	0.48	0.85	4.08E-08
3	CD99	0.62	0.48	3.24	2.24	0.54	4.35E-08
3	PPP1R2	0.64	0.51	4.04	2.84	0.50	5.63E-08
3	ODC1	0.36	0.22	1.61	0.93	0.78	6.70E-08
3	CIB1	0.57	0.41	2.32	1.63	0.51	8.77E-08
3	CCDC88C	0.39	0.25	1.50	0.94	0.67	9.69E-08
3	BCAS2	0.46	0.33	2.20	1.41	0.64	1.15E-07
3	ARHGEF3	0.32	0.20	1.21	0.71	0.78	1.61E-07
3	NDUFB7	0.26	0.15	0.92	0.51	0.84	1.62E-07
3	BTG2	0.64	0.54	5.29	3.25	0.70	1.96E-07
3	BCL2	0.36	0.23	1.40	0.88	0.67	2.09E-07
3	PRNP	0.47	0.35	2.28	1.45	0.65	2.26E-07
3	ARL4C	0.78	0.67	7.69	5.67	0.44	2.26E-07
3	MYO1F	0.31	0.18	1.04	0.74	0.49	2.66E-07
3	TBCB	0.28	0.17	0.91	0.56	0.70	2.66E-07

3	ANKRD37	0.26	0.15	1.17	0.71	0.72	3.05E-07
3	PAXX	0.50	0.36	2.14	1.49	0.52	3.19E-07
3	CAPZB	0.45	0.31	1.60	1.11	0.53	3.60E-07
3	SAP18	0.66	0.54	3.74	2.37	0.66	3.94E-07
3	C12orf57	0.50	0.37	2.10	1.45	0.53	4.69E-07
3	C1orf52	0.30	0.19	0.98	0.61	0.69	1.27E-06
3	DHRS7	0.41	0.28	1.58	1.04	0.59	1.29E-06
3	MARCKSL1	0.29	0.18	1.14	0.68	0.76	1.31E-06
3	SIAH2	0.33	0.22	1.29	0.73	0.83	1.60E-06
3	HMGB2	0.58	0.46	3.57	2.67	0.42	2.22E-06
3	ST8SIA4	0.32	0.20	1.11	0.78	0.50	2.22E-06
3	CITED2	0.32	0.22	1.83	0.96	0.94	2.36E-06
3	WAS	0.26	0.16	0.85	0.52	0.72	2.49E-06
3	PLIN2	0.50	0.40	4.06	2.40	0.76	2.56E-06
3	TENT5A	0.29	0.18	1.14	0.73	0.64	3.07E-06
3	ZBTB11	0.32	0.22	1.21	0.72	0.76	4.13E-06
3	SNHG15	0.48	0.36	2.15	1.49	0.53	4.18E-06
3	GCH1	0.26	0.16	0.88	0.55	0.69	4.28E-06
3	TNFRSF14	0.28	0.18	0.85	0.59	0.52	4.43E-06
3	SIGIRR	0.31	0.20	0.95	0.66	0.52	4.85E-06
3	IVNS1ABP	0.50	0.37	2.35	1.65	0.51	4.89E-06
3	PTMS	0.30	0.19	1.24	0.93	0.42	6.69E-06
3	ZAP70	0.30	0.20	1.08	0.75	0.52	7.43E-06
3	OTUD6B-AS	0.29	0.19	1.07	0.67	0.67	7.74E-06
3	CCDC85B	0.47	0.35	1.97	1.40	0.49	7.77E-06
3	CDKN2D	0.46	0.35	2.11	1.47	0.52	1.13E-05
3	OAT	0.27	0.17	0.94	0.60	0.64	1.16E-05
3	HNRNPU	0.87	0.79	8.92	6.95	0.36	1.34E-05
3	CORO1A	0.66	0.55	3.78	2.68	0.50	1.43E-05
3	H2AZ1	0.77	0.72	8.65	6.14	0.49	1.54E-05
3	BTG3	0.42	0.32	1.98	1.33	0.57	1.70E-05
3	JPT1	0.34	0.25	1.42	0.84	0.76	1.95E-05
3	TAF7	0.62	0.54	3.70	2.52	0.55	2.06E-05
3	PRKACB	0.37	0.26	1.38	0.99	0.48	2.21E-05
3	HERPUD1	0.52	0.43	3.00	1.99	0.59	2.29E-05
3	EBP	0.33	0.23	1.32	0.83	0.67	2.65E-05
3	RASSF1	0.25	0.16	0.86	0.53	0.69	2.88E-05
3	RBM22	0.30	0.20	1.00	0.64	0.63	3.15E-05
3	ABHD3	0.35	0.25	1.35	0.91	0.57	3.42E-05
3	DSTN	0.45	0.33	2.18	1.68	0.37	3.72E-05
3	TBX21	0.33	0.23	1.25	0.86	0.53	3.99E-05
3	SKAP1	0.31	0.22	1.10	0.75	0.54	4.35E-05
3	VPS29	0.26	0.17	0.83	0.57	0.53	4.40E-05
3	HSPA5	0.69	0.65	5.55	4.03	0.46	4.58E-05
3	GMFG	0.41	0.31	1.73	1.14	0.60	4.95E-05
3	POLR1F	0.29	0.20	1.38	0.84	0.71	5.12E-05
3	CDKN1A	0.36	0.25	1.42	1.05	0.44	5.66E-05
3	EPC1	0.45	0.35	1.86	1.34	0.47	5.76E-05
3	MOB4	0.52	0.42	2.56	1.91	0.42	5.83E-05
3	TGFBR3	0.26	0.16	0.94	0.73	0.37	5.84E-05
3	DPP7	0.31	0.21	0.96	0.71	0.43	5.88E-05
3	HLA-DPB1	0.53	0.42	2.55	1.80	0.50	6.09E-05
3	FKBP11	0.26	0.17	0.80	0.61	0.40	6.11E-05
3	CD3E	0.31	0.22	1.26	0.86	0.56	6.15E-05
3	SUN2	0.44	0.34	1.72	1.24	0.47	6.22E-05
3	ITK	0.30	0.20	1.01	0.72	0.49	9.06E-05
3	TECR	0.35	0.24	1.22	0.85	0.52	9.88E-05
3	ELOC	0.36	0.27	1.25	0.87	0.53	9.95E-05
3	HLA-DPA1	0.48	0.38	2.32	1.62	0.52	0.00010411

3	NDUFB8	0.40	0.30	1.46	1.04	0.49	0.00010763
3	HSPA4	0.45	0.36	1.99	1.45	0.46	0.00010837
3	TERF1	0.31	0.21	1.17	0.81	0.53	0.00010894
3	IER2	0.79	0.74	9.17	7.14	0.36	0.00010977
3	ARPC4	0.33	0.23	1.17	0.74	0.66	0.00011099
3	FKBP4	0.54	0.47	4.89	3.18	0.62	0.00011313
3	NSD1	0.27	0.18	0.85	0.62	0.45	0.00011344
3	TAOK3	0.38	0.28	1.44	1.07	0.43	0.00011841
3	RNASEK	0.26	0.17	0.85	0.61	0.48	0.00012361
3	IER5	0.58	0.51	5.31	3.52	0.59	0.00012847
3	MBNL2	0.28	0.19	1.01	0.70	0.54	0.00013426
3	GSTK1	0.28	0.19	0.88	0.65	0.46	0.00015415
3	CASP8	0.39	0.29	1.51	1.07	0.49	0.00015675
3	MLLT6	0.37	0.27	1.38	1.03	0.42	0.0001946
3	HERPUD2	0.31	0.22	1.16	0.81	0.51	0.00026455
3	PNPLA8	0.42	0.32	1.55	1.20	0.37	0.00035991
3	CCNH	0.46	0.37	2.12	1.50	0.50	0.00037409
3	NABP1	0.32	0.24	1.35	0.98	0.46	0.00041159
3	GTF3C1	0.29	0.21	1.37	0.86	0.67	0.00044168
3	LAMTOR4	0.34	0.25	1.18	0.85	0.47	0.0004437
3	LBH	0.38	0.28	1.61	1.22	0.40	0.00046124
3	COX8A	0.52	0.43	2.18	1.64	0.41	0.00046225
3	SEC11C	0.26	0.19	0.93	0.64	0.54	0.00047905
3	SLC3A2	0.61	0.55	3.41	2.58	0.40	0.00049014
3	STIP1	0.48	0.43	2.50	1.76	0.51	0.00057561
3	COQ10B	0.33	0.26	1.15	0.85	0.43	0.00069139
3	CNN2	0.43	0.34	1.79	1.35	0.41	0.00081182
3	ARHGEF1	0.41	0.33	1.68	1.26	0.42	0.00086887
3	UGP2	0.33	0.25	1.21	0.93	0.38	0.00088648
3	HMGN2	0.51	0.44	2.38	1.83	0.38	0.00094328
3	CYRIB	0.42	0.33	1.61	1.22	0.40	0.00103401
3	CYFIP2	0.26	0.19	0.84	0.63	0.42	0.00105311
3	SNHG9	0.26	0.19	1.07	0.79	0.44	0.00114406
3	ENSG0000(	0.27	0.20	1.22	0.80	0.62	0.00116483
3	ABI1	0.32	0.23	1.02	0.78	0.39	0.00119741
3	UQCR11	0.34	0.26	1.25	0.94	0.41	0.00119926
3	CD52	0.42	0.36	2.39	1.52	0.66	0.00120802
3	PTGER4	0.51	0.46	3.39	2.34	0.54	0.00126454
3	OSER1	0.27	0.20	0.84	0.60	0.47	0.00140749
3	GLUL	0.30	0.23	1.27	0.88	0.53	0.00153683
3	TNFAIP8	0.29	0.21	1.18	0.87	0.44	0.00154824
3	VAMP2	0.51	0.44	2.40	1.81	0.41	0.0015762
3	H2AX	0.33	0.26	1.31	0.97	0.43	0.00193414
3	COPE	0.35	0.28	1.30	0.97	0.43	0.00195086
3	IER3	0.30	0.23	1.77	1.23	0.53	0.00201245
3	TAF10	0.29	0.22	0.87	0.67	0.37	0.00231477
3	DYNLL2	0.31	0.23	1.02	0.75	0.45	0.0023634
3	ENSG0000(	0.33	0.26	1.33	0.96	0.46	0.0024156
3	RASAL3	0.26	0.18	0.83	0.64	0.37	0.002529
3	SS18L2	0.31	0.24	1.12	0.77	0.54	0.00255546
3	SNHG5	0.59	0.53	3.56	2.59	0.46	0.00314941
3	DAD1	0.29	0.22	0.99	0.77	0.37	0.00333506
3	SEM1	0.27	0.21	0.92	0.68	0.43	0.00409162
3	BAX	0.28	0.21	0.92	0.70	0.40	0.00415708
3	TRAPPC4	0.25	0.19	0.82	0.60	0.44	0.00486694
3	MT2A	0.56	0.47	8.58	6.01	0.51	0.0051235
3	TSC22D4	0.26	0.20	0.92	0.68	0.45	0.00539738
3	THAP9-AS1	0.31	0.24	1.07	0.83	0.37	0.00568421
3	NDUFA12	0.34	0.27	1.22	0.92	0.41	0.00622453

3	NFKBIB	0.26	0.20	0.86	0.65	0.40	0.00642927
3	C1orf21	0.33	0.27	1.32	1.01	0.39	0.00652712
3	UHMK1	0.28	0.21	0.92	0.71	0.37	0.00674031
3	DERL1	0.31	0.24	0.98	0.76	0.37	0.00702283
3	NSMCE3	0.31	0.25	1.18	0.87	0.44	0.00737554
3	ARL6IP4	0.27	0.21	0.94	0.69	0.45	0.00810109
3	BANF1	0.28	0.22	0.96	0.70	0.46	0.00940342
3	SELENOK	0.74	0.71	6.40	4.81	0.41	0.0108514
3	HCP5	0.25	0.20	0.91	0.64	0.52	0.01280983
3	ATP5PO	0.26	0.21	0.86	0.63	0.44	0.01523144
3	POLR1H	0.31	0.25	1.11	0.85	0.39	0.01557322
3	PRR13	0.33	0.27	1.15	0.89	0.38	0.01677422
3	SNRPB2	0.33	0.27	1.22	0.92	0.40	0.01712549
3	CHMP1B	0.34	0.28	1.48	1.09	0.44	0.02469938
3	PRMT2	0.34	0.29	1.44	1.10	0.40	0.02742686
3	SMIM26	0.26	0.21	0.85	0.66	0.37	0.02825133
3	LPIN2	0.25	0.21	0.99	0.71	0.48	0.02979539
3	CMC2	0.31	0.27	1.13	0.87	0.37	0.03360671
3	GTF2B	0.28	0.24	1.03	0.79	0.38	0.0469592
3	TUBA1C	0.30	0.26	1.21	0.87	0.47	0.04772087
4	ITGA1	0.53	0.10	3.74	0.49	2.94	3.88E-55
4	ACP5	0.26	0.03	1.08	0.08	3.69	1.55E-45
4	LINC02446	0.46	0.12	6.54	1.05	2.64	1.03E-34
4	CXCR6	0.38	0.08	1.89	0.32	2.56	7.74E-34
4	LGALS3	0.51	0.17	2.48	0.61	2.03	2.19E-27
4	HLA-DQA1	0.52	0.20	3.62	0.88	2.03	3.74E-22
4	CD74	0.95	0.72	10.66	5.21	1.03	7.63E-22
4	CCL5	0.96	0.81	39.51	20.38	0.96	7.75E-22
4	HLA-DRB1	0.83	0.67	16.54	5.42	1.61	6.56E-21
4	HLA-DRA	0.74	0.47	7.03	2.34	1.59	3.24E-20
4	CSF1	0.28	0.07	1.68	0.41	2.02	7.98E-20
4	ENTPD1	0.32	0.09	2.13	0.44	2.26	1.07E-19
4	LDLRAD4	0.35	0.11	1.80	0.46	1.96	1.44E-18
4	HLA-DPA1	0.68	0.38	3.70	1.59	1.22	2.42E-17
4	LINC01871	0.73	0.43	7.25	2.89	1.33	2.98E-17
4	ALOX5AP	0.57	0.27	3.58	1.36	1.40	3.46E-16
4	GZMA	0.70	0.44	22.77	4.60	2.31	1.28E-15
4	SRGAP3	0.27	0.08	1.23	0.31	1.97	3.90E-15
4	TBCD	0.26	0.08	1.14	0.26	2.11	1.12E-14
4	GZMK	0.54	0.28	6.67	1.91	1.80	1.22E-14
4	CD7	0.91	0.74	11.66	6.65	0.81	3.58E-14
4	GZMB	0.90	0.67	25.17	13.33	0.92	3.93E-14
4	COTL1	0.68	0.39	4.44	2.20	1.01	8.30E-14
4	HLA-DMA	0.31	0.11	1.14	0.33	1.80	1.13E-13
4	C4orf48	0.26	0.08	0.79	0.25	1.64	1.43E-13
4	TTN	0.34	0.13	1.29	0.53	1.28	3.07E-13
4	GAPDH	0.97	0.90	23.29	14.73	0.66	3.97E-13
4	LAG3	0.29	0.10	1.17	0.39	1.57	7.65E-13
4	ATP8B4	0.36	0.14	1.25	0.49	1.35	1.57E-12
4	HLA-DQB1	0.47	0.23	2.34	0.89	1.40	2.25E-12
4	PRF1	0.82	0.55	7.24	4.00	0.86	4.24E-12
4	SAMSN1	0.76	0.53	5.52	2.97	0.89	9.15E-12
4	PTMS	0.41	0.20	2.40	0.87	1.46	1.56E-11
4	KLRC2	0.60	0.35	3.56	1.74	1.03	3.49E-11
4	IKZF3	0.61	0.35	4.37	1.93	1.18	3.55E-11
4	TNFRSF9	0.52	0.31	6.05	2.56	1.24	3.73E-11
4	SH2D1B	0.55	0.30	2.52	1.32	0.93	7.23E-11
4	YWHAB	0.82	0.60	5.64	3.18	0.83	8.52E-11
4	IRF4	0.33	0.14	1.65	0.56	1.56	1.05E-10

4	PIN1	0.26	0.09	0.80	0.28	1.51	1.47E-10
4	CARD16	0.47	0.24	2.03	0.97	1.06	1.88E-10
4	CKLF	0.46	0.24	2.36	0.90	1.39	3.09E-10
4	HLA-DPB1	0.64	0.43	3.41	1.81	0.91	5.48E-10
4	MXD4	0.58	0.34	2.51	1.33	0.92	6.51E-10
4	STAT5A	0.46	0.24	1.85	0.97	0.93	6.81E-10
4	GALNT2	0.27	0.11	1.03	0.32	1.66	1.20E-09
4	SLA2	0.48	0.28	2.43	1.14	1.09	1.85E-09
4	TRDC	0.70	0.50	4.98	2.74	0.86	5.30E-09
4	OST4	0.83	0.63	4.85	3.16	0.62	8.07E-09
4	OSTF1	0.58	0.35	2.42	1.31	0.88	8.88E-09
4	GNLY	0.89	0.85	100.65	53.09	0.92	1.03E-08
4	CD96	0.58	0.38	3.14	1.72	0.86	1.18E-08
4	PPP1CA	0.43	0.22	1.36	0.74	0.87	1.36E-08
4	TTC1	0.30	0.13	0.83	0.39	1.10	1.89E-08
4	BST2	0.56	0.36	2.79	1.43	0.96	1.99E-08
4	RASAL3	0.36	0.18	1.34	0.62	1.11	2.16E-08
4	LCP1	0.90	0.78	9.87	6.80	0.54	3.43E-08
4	SURF4	0.54	0.33	2.06	1.23	0.74	3.52E-08
4	PLAAT4	0.54	0.33	2.61	1.33	0.98	3.64E-08
4	RBX1	0.48	0.27	1.81	0.94	0.94	3.65E-08
4	IFI27L2	0.28	0.12	0.93	0.42	1.16	4.45E-08
4	APOBEC3G	0.71	0.51	4.30	2.67	0.69	5.36E-08
4	CYTOR	0.52	0.32	2.88	1.28	1.17	6.36E-08
4	SUMO2	0.89	0.77	6.71	4.81	0.48	8.10E-08
4	AKAP5	0.29	0.13	0.97	0.48	1.02	1.21E-07
4	KIR2DL4	0.26	0.11	1.05	0.42	1.31	1.22E-07
4	COX6C	0.60	0.40	2.53	1.58	0.68	1.50E-07
4	ITGAE	0.31	0.15	1.22	0.54	1.17	2.36E-07
4	TIGIT	0.46	0.29	2.75	1.36	1.02	2.40E-07
4	GIMAP4	0.42	0.24	1.87	1.01	0.89	2.59E-07
4	SMC3	0.52	0.32	2.14	1.24	0.79	3.42E-07
4	NDUFC1	0.25	0.11	0.76	0.35	1.14	4.62E-07
4	CD52	0.55	0.36	2.62	1.58	0.73	6.02E-07
4	RALA	0.42	0.25	1.69	0.84	1.02	6.42E-07
4	NCK1	0.37	0.20	1.35	0.64	1.07	6.76E-07
4	CALM3	0.43	0.26	1.68	0.86	0.97	7.09E-07
4	ITM2A	0.58	0.37	3.46	2.03	0.77	7.18E-07
4	COX7A2	0.67	0.47	3.01	1.92	0.65	7.77E-07
4	ARPC3	0.76	0.60	4.61	2.96	0.64	9.26E-07
4	ELOB	0.72	0.49	2.97	2.09	0.51	9.44E-07
4	RGS10	0.35	0.19	1.30	0.62	1.07	9.61E-07
4	IL32	0.72	0.56	9.01	5.90	0.61	1.05E-06
4	UQCRB	0.79	0.67	5.42	3.71	0.55	1.12E-06
4	HDAC1	0.31	0.15	0.78	0.45	0.80	1.28E-06
4	PSMB9	0.54	0.35	2.13	1.40	0.60	1.28E-06
4	SURF2	0.27	0.13	0.86	0.40	1.11	1.47E-06
4	CD2	0.74	0.59	6.58	4.02	0.71	1.49E-06
4	GGA2	0.35	0.19	1.31	0.65	1.01	1.91E-06
4	CARD19	0.42	0.24	1.50	0.83	0.85	1.91E-06
4	CAP1	0.58	0.40	2.36	1.60	0.56	2.14E-06
4	PMF1	0.27	0.13	0.84	0.40	1.07	2.36E-06
4	CORO1A	0.74	0.56	4.20	2.76	0.61	2.39E-06
4	ARPC1B	0.52	0.32	1.88	1.17	0.69	2.47E-06
4	APOBEC3C	0.33	0.18	1.40	0.63	1.16	2.48E-06
4	SERPINB1	0.48	0.31	2.37	1.35	0.82	2.76E-06
4	GPRIN3	0.56	0.36	2.61	1.53	0.77	2.88E-06
4	PRDX1	0.66	0.49	4.55	2.57	0.82	2.91E-06
4	ARPC4	0.39	0.24	1.43	0.77	0.90	3.03E-06

4	BAX	0.37	0.21	1.21	0.70	0.80	3.45E-06
4	GLIPR1	0.49	0.31	2.14	1.36	0.66	3.58E-06
4	NDUFB4	0.49	0.32	1.85	1.08	0.77	3.58E-06
4	TRG-AS1	0.50	0.32	2.34	1.46	0.68	3.79E-06
4	NDUFA13	0.51	0.32	1.86	1.15	0.69	3.87E-06
4	C19orf53	0.59	0.40	2.38	1.51	0.66	4.38E-06
4	NEDD8	0.44	0.26	1.42	0.88	0.69	4.61E-06
4	FABP5	0.28	0.14	1.05	0.57	0.88	4.85E-06
4	SPCS3	0.53	0.33	1.93	1.22	0.66	5.13E-06
4	LMAN2	0.40	0.23	1.28	0.77	0.73	5.43E-06
4	GBP5	0.44	0.28	2.42	1.36	0.84	5.61E-06
4	LAT2	0.46	0.29	2.13	1.13	0.92	5.70E-06
4	SLC7A5	0.54	0.39	3.21	1.75	0.88	5.91E-06
4	PTTG1	0.29	0.15	0.99	0.59	0.74	6.40E-06
4	PARK7	0.61	0.46	3.21	1.98	0.70	6.57E-06
4	FAS	0.36	0.20	1.34	0.77	0.81	8.13E-06
4	ATP5MG	0.82	0.70	5.75	3.94	0.54	8.14E-06
4	EP300	0.40	0.24	1.24	0.78	0.66	8.41E-06
4	SPCS1	0.41	0.25	1.37	0.82	0.74	8.45E-06
4	UBL5	0.77	0.63	4.30	3.08	0.48	9.69E-06
4	UQCRC2	0.40	0.24	1.44	0.80	0.85	1.00E-05
4	CLEC2D	0.62	0.47	4.96	3.11	0.67	1.06E-05
4	LSM10	0.29	0.15	0.85	0.46	0.90	1.15E-05
4	CHCHD10	0.27	0.14	0.87	0.43	1.03	1.16E-05
4	SEC61G	0.53	0.35	2.24	1.34	0.74	1.25E-05
4	PRDX5	0.44	0.27	1.57	0.96	0.71	1.34E-05
4	GIMAP7	0.41	0.24	1.72	1.07	0.68	1.43E-05
4	APBB1IP	0.44	0.27	1.59	1.02	0.64	1.48E-05
4	ATP2A2	0.34	0.19	1.18	0.63	0.91	1.64E-05
4	NDUFAB1	0.34	0.19	1.02	0.59	0.79	1.78E-05
4	GZMH	0.56	0.37	4.42	3.02	0.55	1.94E-05
4	LSM2	0.31	0.17	0.95	0.53	0.83	2.04E-05
4	SMC4	0.38	0.23	1.92	0.83	1.21	2.20E-05
4	SLFN12L	0.46	0.29	2.18	1.28	0.76	2.27E-05
4	PBX4	0.27	0.14	0.99	0.53	0.91	2.27E-05
4	EVL	0.48	0.32	2.51	1.39	0.86	2.29E-05
4	TRAF5	0.35	0.21	1.38	0.78	0.82	2.55E-05
4	PGK1	0.72	0.57	4.81	3.42	0.49	2.55E-05
4	HOPX	0.62	0.46	3.81	2.60	0.55	2.77E-05
4	TBC1D10C	0.31	0.17	1.25	0.64	0.97	2.82E-05
4	SRSF9	0.61	0.45	2.56	1.78	0.53	3.07E-05
4	CFL1	0.93	0.84	9.98	7.63	0.39	3.20E-05
4	PSMA7	0.74	0.59	4.00	3.01	0.41	3.25E-05
4	MYL12B	0.85	0.74	6.72	5.07	0.41	3.42E-05
4	SYNGR2	0.28	0.15	0.89	0.49	0.86	3.43E-05
4	ROMO1	0.47	0.30	1.53	1.06	0.53	3.51E-05
4	SNRPF	0.44	0.29	1.66	1.00	0.73	3.56E-05
4	USP48	0.25	0.14	0.87	0.41	1.07	3.80E-05
4	TSPO	0.37	0.24	1.46	0.78	0.89	3.94E-05
4	PTPN22	0.66	0.52	4.00	2.72	0.56	3.94E-05
4	ASXL2	0.27	0.14	0.99	0.48	1.06	4.18E-05
4	MYH9	0.75	0.56	3.91	2.85	0.46	4.38E-05
4	EDF1	0.80	0.70	5.28	3.88	0.44	4.43E-05
4	PKM	0.52	0.35	2.24	1.55	0.53	4.61E-05
4	SLFN5	0.44	0.28	2.06	1.29	0.67	4.64E-05
4	UBE2L3	0.44	0.28	1.47	0.92	0.67	4.82E-05
4	SRI	0.39	0.24	1.30	0.80	0.69	5.21E-05
4	GSTO1	0.46	0.30	1.58	1.09	0.53	5.22E-05
4	PPP1R14B	0.45	0.30	2.13	1.23	0.80	5.44E-05

4	OCIAD2	0.26	0.14	0.91	0.42	1.11	5.96E-05
4	PRRC2A	0.28	0.15	0.79	0.43	0.87	6.05E-05
4	PIK3AP1	0.40	0.24	1.26	0.85	0.57	6.10E-05
4	TNFSF10	0.28	0.16	1.21	0.63	0.94	6.32E-05
4	GSPT1	0.58	0.43	3.36	2.02	0.74	6.42E-05
4	PPP1R16B	0.41	0.27	1.60	0.94	0.77	6.43E-05
4	COPS6	0.28	0.16	0.87	0.49	0.82	6.86E-05
4	ABI3	0.28	0.15	0.96	0.56	0.77	6.92E-05
4	LPIN2	0.34	0.20	1.15	0.73	0.66	7.17E-05
4	ITM2C	0.44	0.29	2.45	1.41	0.80	7.49E-05
4	SYTL2	0.27	0.15	1.12	0.55	1.01	7.53E-05
4	CD81	0.72	0.59	4.05	2.89	0.49	7.66E-05
4	WNK1	0.50	0.35	2.11	1.36	0.63	7.76E-05
4	HMGB2	0.62	0.47	4.02	2.72	0.56	7.82E-05
4	CHST12	0.50	0.34	2.31	1.56	0.57	9.29E-05
4	TMA7	0.79	0.71	6.12	4.30	0.51	9.42E-05
4	UBA2	0.32	0.19	1.03	0.58	0.84	9.63E-05
4	MDFIC	0.26	0.14	0.99	0.50	1.00	9.66E-05
4	SEPTIN7	0.87	0.73	6.03	4.59	0.39	9.83E-05
4	PRPF4B	0.66	0.49	3.22	2.40	0.42	9.89E-05
4	DUSP4	0.44	0.31	3.00	1.80	0.73	0.00010251
4	PSMB8	0.57	0.43	2.57	1.67	0.63	0.00010969
4	RBPJ	0.50	0.36	2.61	1.64	0.67	0.00011781
4	CHST11	0.31	0.18	1.03	0.59	0.79	0.00012105
4	CDC42SE2	0.80	0.67	4.98	3.76	0.41	0.00012224
4	MGST3	0.29	0.17	0.93	0.53	0.81	0.00012328
4	TMCO1	0.30	0.18	1.11	0.59	0.91	0.00012346
4	CLDND1	0.60	0.47	4.16	2.59	0.68	0.00013671
4	COX8A	0.58	0.43	2.42	1.67	0.53	0.00013839
4	PSMB1	0.55	0.41	2.33	1.56	0.58	0.00013921
4	OAZ1	0.93	0.91	17.28	13.15	0.39	0.00014032
4	PPP1CC	0.37	0.22	1.03	0.72	0.53	0.00015789
4	FIS1	0.33	0.20	1.09	0.64	0.76	0.00017022
4	HDLBP	0.33	0.20	1.17	0.66	0.84	0.00017686
4	NDUFS5	0.67	0.51	3.09	2.30	0.43	0.00019252
4	STUB1	0.38	0.24	1.20	0.77	0.64	0.00019427
4	IL2RB	0.71	0.57	4.36	3.16	0.47	0.00020183
4	PSMA4	0.39	0.26	1.43	0.86	0.73	0.00020729
4	SRP14	0.87	0.80	7.84	6.06	0.37	0.00021505
4	CD99	0.62	0.50	3.42	2.32	0.56	0.00021616
4	EMC7	0.39	0.24	1.06	0.75	0.50	0.000218
4	CIAO1	0.31	0.18	0.88	0.58	0.59	0.00022292
4	ITGB1	0.82	0.74	7.61	5.77	0.40	0.0002322
4	PRDX2	0.25	0.14	0.77	0.45	0.78	0.00023606
4	MPC2	0.36	0.22	0.96	0.67	0.52	0.00024025
4	PET100	0.39	0.25	1.31	0.89	0.57	0.00025622
4	DNAJB14	0.44	0.30	1.43	1.00	0.52	0.00025962
4	SEM1	0.34	0.21	1.08	0.69	0.64	0.00026181
4	REEP5	0.41	0.28	1.46	0.90	0.70	0.00026462
4	ANXA2	0.49	0.33	1.88	1.39	0.43	0.00026496
4	RABAC1	0.52	0.38	2.17	1.45	0.58	0.00027194
4	PPM1G	0.52	0.35	1.83	1.34	0.45	0.00027232
4	CLTC	0.29	0.17	0.92	0.50	0.87	0.00028048
4	PPP4C	0.35	0.22	1.12	0.74	0.61	0.00028814
4	ANXA5	0.34	0.21	1.41	0.80	0.82	0.00029443
4	NDUFB11	0.56	0.43	2.23	1.59	0.49	0.00029747
4	LAMTOR5	0.42	0.27	1.33	0.90	0.57	0.00030108
4	NUDC	0.59	0.44	2.72	1.93	0.49	0.00030367
4	PDCD4	0.61	0.47	3.57	2.32	0.62	0.00031108

4	PGAM1	0.38	0.26	1.39	0.89	0.65	0.00031187
4	PPP1R35	0.28	0.16	0.79	0.52	0.60	0.00031795
4	ZBTB38	0.31	0.18	1.18	0.70	0.76	0.00033565
4	MSI2	0.27	0.16	0.83	0.48	0.79	0.00033776
4	DNAJA2	0.55	0.39	1.96	1.36	0.53	0.0003491
4	NAMPT	0.66	0.53	4.79	3.71	0.37	0.00035462
4	RPS27L	0.52	0.35	1.88	1.39	0.44	0.00035789
4	PRKACB	0.40	0.27	1.49	1.02	0.55	0.00038278
4	NOL7	0.41	0.27	1.30	0.93	0.49	0.00040295
4	N4BP1	0.41	0.28	1.49	1.02	0.55	0.00041693
4	NDUFA12	0.41	0.27	1.38	0.94	0.55	0.0004435
4	CASP4	0.40	0.27	1.31	0.85	0.62	0.00044509
4	FASLG	0.42	0.28	1.66	1.14	0.55	0.00046621
4	CIAO2B	0.48	0.35	1.78	1.28	0.47	0.00046651
4	SUB1	0.81	0.74	5.81	4.52	0.36	0.00049231
4	ATP5MK	0.47	0.33	1.83	1.22	0.59	0.00049362
4	RNF213	0.70	0.55	4.92	3.25	0.60	0.00050155
4	SHFL	0.31	0.19	1.09	0.61	0.83	0.00050512
4	PSME2	0.50	0.38	2.19	1.45	0.59	0.00051491
4	TMEM248	0.31	0.18	0.82	0.56	0.55	0.00052424
4	DR1	0.42	0.28	1.37	0.96	0.51	0.00052705
4	HUWE1	0.48	0.36	2.25	1.37	0.71	0.00053102
4	PAIP2	0.66	0.50	3.26	2.31	0.50	0.00054209
4	SRP19	0.38	0.25	1.15	0.81	0.51	0.00055271
4	CAPG	0.27	0.16	1.08	0.59	0.87	0.00055378
4	NDUFA6	0.43	0.30	1.50	1.01	0.57	0.0005816
4	ANXA7	0.27	0.16	0.76	0.46	0.72	0.00060142
4	LCK	0.42	0.29	1.55	1.03	0.59	0.00060619
4	TXK	0.36	0.25	2.00	1.04	0.95	0.00062237
4	SPN	0.45	0.30	1.66	1.14	0.54	0.00062721
4	ATP5IF1	0.48	0.34	1.86	1.33	0.48	0.00062801
4	ACTN4	0.66	0.54	3.46	2.54	0.45	0.0006389
4	RTF2	0.36	0.22	0.98	0.70	0.47	0.00065278
4	METAP2	0.29	0.19	1.16	0.62	0.89	0.00066531
4	COMMD6	0.74	0.59	3.80	2.87	0.40	0.00067979
4	FAM3C	0.31	0.19	0.97	0.60	0.70	0.00068286
4	NDUFB8	0.44	0.31	1.55	1.08	0.52	0.00068925
4	LSP1	0.49	0.35	2.11	1.44	0.55	0.00069654
4	DOCK10	0.35	0.22	1.10	0.74	0.57	0.00071228
4	CCT7	0.32	0.20	0.90	0.60	0.59	0.00072134
4	ATP5MF	0.41	0.29	1.55	1.05	0.56	0.00072606
4	CCT6A	0.47	0.34	1.58	1.18	0.43	0.00074671
4	CREBZF	0.46	0.32	1.71	1.24	0.47	0.00076839
4	LY6E	0.42	0.30	1.86	1.10	0.75	0.00077539
4	SELENOW	0.50	0.37	2.27	1.55	0.55	0.0007986
4	SAMD3	0.62	0.48	3.27	2.44	0.42	0.00082486
4	TPR	0.52	0.40	2.48	1.70	0.54	0.00082826
4	UQCR10	0.45	0.30	1.63	1.11	0.56	0.00083871
4	NIBAN1	0.33	0.22	1.41	0.89	0.66	0.00084009
4	PDCL3	0.29	0.18	0.96	0.60	0.69	0.00086409
4	MAP2K2	0.46	0.32	1.53	1.09	0.49	0.00087134
4	TIAL1	0.44	0.30	1.58	1.11	0.51	0.00088609
4	FKBP4	0.58	0.47	4.80	3.35	0.52	0.00089409
4	PSMB2	0.32	0.21	1.06	0.64	0.73	0.00092309
4	RAB27A	0.47	0.35	2.21	1.36	0.70	0.00092802
4	PSMB4	0.40	0.28	1.29	0.87	0.57	0.00094249
4	DYNLT1	0.35	0.23	1.20	0.79	0.60	0.0009462
4	PPDPF	0.52	0.41	2.21	1.55	0.52	0.0009568
4	BLOC1S1	0.31	0.19	0.88	0.62	0.50	0.00096875

4	COPB2	0.26	0.15	0.71	0.46	0.63	0.00097198
4	STAT5B	0.26	0.16	0.83	0.56	0.57	0.00097538
4	GPR174	0.36	0.24	1.39	0.88	0.67	0.00102291
4	ATP5F1C	0.36	0.24	1.28	0.82	0.64	0.00103677
4	MZT2B	0.64	0.48	2.45	1.88	0.38	0.00104248
4	OTUB1	0.27	0.16	0.90	0.50	0.86	0.00105361
4	MAGOH	0.40	0.28	1.37	0.95	0.54	0.0010704
4	TNIP2	0.31	0.19	0.96	0.61	0.64	0.0010885
4	GSTP1	0.59	0.45	2.84	2.05	0.47	0.00110985
4	ZMAT2	0.29	0.18	0.84	0.53	0.66	0.00114701
4	ACTB	0.91	0.85	14.30	11.12	0.36	0.00125095
4	EIF3G	0.68	0.57	3.71	2.65	0.49	0.00130466
4	SNRPG	0.57	0.44	2.23	1.72	0.37	0.00131534
4	LPIN1	0.36	0.25	1.34	0.89	0.59	0.00135455
4	ATP5PF	0.38	0.27	1.38	0.90	0.62	0.0013644
4	CSTB	0.38	0.26	1.31	0.92	0.52	0.00143397
4	ATP6V1F	0.38	0.27	1.32	0.85	0.64	0.00145744
4	TTC39C	0.36	0.24	1.26	0.86	0.55	0.00152949
4	GADD45GIF	0.33	0.21	1.03	0.69	0.59	0.00154616
4	NCOA7	0.29	0.18	1.12	0.71	0.65	0.00155413
4	COX7A2L	0.37	0.25	1.30	0.86	0.58	0.00156238
4	METTL9	0.33	0.21	0.93	0.68	0.46	0.00158021
4	SOD1	0.70	0.66	6.92	5.00	0.47	0.00165047
4	TYROBP	0.81	0.74	7.04	5.41	0.38	0.00167004
4	DYNLRB1	0.32	0.21	0.98	0.67	0.56	0.00168501
4	PRELID1	0.56	0.44	2.46	1.80	0.45	0.00171543
4	HEBP2	0.27	0.16	0.72	0.49	0.55	0.0017328
4	MAP4	0.29	0.19	1.04	0.61	0.77	0.00174124
4	STOM	0.40	0.29	1.49	1.03	0.53	0.00178867
4	CALCOCO2	0.35	0.24	1.14	0.76	0.57	0.00183659
4	STXBP3	0.31	0.19	0.92	0.61	0.59	0.00184712
4	TRAPPC1	0.31	0.19	0.95	0.61	0.65	0.00197596
4	HSBP1	0.26	0.16	0.68	0.48	0.50	0.00204121
4	IK	0.52	0.39	2.13	1.53	0.48	0.00205276
4	LSM8	0.43	0.30	1.48	1.07	0.47	0.00210252
4	HCLS1	0.42	0.31	1.57	1.12	0.49	0.0021403
4	SSU72	0.32	0.21	1.07	0.66	0.70	0.00218991
4	NDUFA1	0.50	0.39	1.98	1.46	0.44	0.00225024
4	SPCS2	0.50	0.36	1.77	1.37	0.37	0.0022747
4	PAK2	0.56	0.43	2.48	1.88	0.40	0.00229788
4	MDH2	0.25	0.16	0.76	0.48	0.65	0.00243536
4	RASGRP1	0.32	0.21	1.20	0.82	0.55	0.00244259
4	PSMB3	0.33	0.23	1.14	0.73	0.65	0.00245132
4	TIMM13	0.26	0.16	0.68	0.48	0.50	0.00250163
4	COPE	0.39	0.28	1.56	0.98	0.67	0.00257974
4	EIF2S3	0.45	0.34	1.61	1.20	0.42	0.00268673
4	C21orf91	0.27	0.18	0.92	0.57	0.69	0.00271509
4	LDHB	0.41	0.29	1.44	1.02	0.50	0.00277367
4	VPS28	0.34	0.23	1.17	0.78	0.58	0.00278208
4	SF3B6	0.45	0.33	1.64	1.16	0.51	0.00289528
4	NDUFB1	0.33	0.22	1.36	0.78	0.80	0.00289656
4	SYNRG	0.40	0.29	1.44	1.03	0.48	0.00294891
4	GSTK1	0.31	0.20	0.99	0.66	0.58	0.00297767
4	ATP5MC3	0.53	0.43	2.25	1.67	0.43	0.00306648
4	TBL1XR1	0.37	0.26	1.23	0.91	0.43	0.00309668
4	PSMD13	0.39	0.29	1.51	1.04	0.54	0.00328867
4	ABCB1	0.27	0.17	1.06	0.60	0.82	0.00333687
4	ANXA6	0.34	0.24	1.19	0.77	0.62	0.00333808
4	MRPL57	0.31	0.20	0.95	0.63	0.60	0.00340319

4	BATF	0.25	0.16	0.87	0.57	0.60	0.00345672
4	TMEM258	0.40	0.29	1.47	1.07	0.46	0.00346615
4	MZT2A	0.39	0.28	1.32	0.90	0.54	0.0035223
4	PIK3CG	0.28	0.18	1.00	0.60	0.75	0.00355014
4	GID8	0.26	0.16	0.68	0.48	0.50	0.00358396
4	PTPN1	0.31	0.20	0.97	0.67	0.53	0.00365414
4	SUPT16H	0.31	0.21	1.02	0.69	0.55	0.00374661
4	BRK1	0.40	0.28	1.21	0.90	0.43	0.00380131
4	MAN1A1	0.26	0.17	0.98	0.57	0.78	0.00385305
4	PDCD10	0.34	0.24	1.19	0.79	0.59	0.00390731
4	UBA6	0.32	0.21	0.89	0.68	0.39	0.00406429
4	HARS1	0.25	0.16	0.65	0.47	0.45	0.0040682
4	PDIA6	0.40	0.28	1.38	1.00	0.47	0.00415131
4	PSMD8	0.50	0.36	1.60	1.21	0.40	0.0042155
4	SERINC1	0.48	0.37	1.79	1.34	0.42	0.00422789
4	RAB10	0.34	0.26	1.34	0.83	0.69	0.00424373
4	MYDGF	0.30	0.20	0.94	0.69	0.43	0.00460767
4	NCBP3	0.29	0.19	0.97	0.60	0.71	0.00469313
4	MRPL33	0.36	0.26	1.41	0.94	0.59	0.00485459
4	COX14	0.39	0.27	1.18	0.89	0.41	0.00488572
4	PSMA5	0.38	0.29	1.58	1.01	0.64	0.00490216
4	RESF1	0.50	0.38	2.33	1.74	0.42	0.0049066
4	POMP	0.54	0.44	2.45	1.81	0.44	0.00514296
4	ROCK1	0.53	0.42	2.29	1.70	0.43	0.00516584
4	TMEM160	0.29	0.20	0.93	0.66	0.51	0.00530237
4	GTF2I	0.41	0.30	1.48	1.07	0.47	0.00542312
4	CYSTM1	0.36	0.25	1.11	0.84	0.40	0.0054688
4	CCND2	0.55	0.44	2.66	2.01	0.41	0.00566979
4	DHRS7	0.40	0.29	1.55	1.10	0.50	0.00570529
4	TYMP	0.26	0.17	0.87	0.61	0.51	0.00581002
4	CIB1	0.54	0.43	2.24	1.70	0.40	0.00585516
4	NCKAP1L	0.31	0.22	1.15	0.73	0.65	0.00591042
4	NDUFB10	0.37	0.26	1.18	0.89	0.41	0.00591809
4	MRPL52	0.29	0.19	0.83	0.59	0.49	0.00595367
4	ZNF217	0.31	0.21	1.12	0.73	0.62	0.00597755
4	STAM	0.41	0.31	1.65	1.21	0.45	0.00601749
4	SPSB3	0.40	0.29	1.48	0.99	0.58	0.00619557
4	BACH1	0.27	0.18	0.84	0.57	0.55	0.00620829
4	VTI1B	0.25	0.16	0.71	0.52	0.46	0.00627224
4	NT5C	0.30	0.20	0.98	0.64	0.61	0.00631349
4	TMF1	0.48	0.36	1.77	1.35	0.39	0.00635146
4	EIF3M	0.38	0.26	1.13	0.85	0.42	0.00639672
4	HERC1	0.28	0.19	1.05	0.61	0.78	0.00644101
4	MIS18BP1	0.35	0.24	1.20	0.87	0.46	0.00653551
4	MRPL51	0.31	0.21	0.96	0.68	0.50	0.00665161
4	LSM7	0.38	0.28	1.31	0.93	0.50	0.00699596
4	ITM2B	0.77	0.64	4.70	3.52	0.42	0.00722192
4	NIPBL	0.50	0.39	2.24	1.60	0.48	0.00730384
4	PITPNC1	0.38	0.28	1.52	1.11	0.46	0.00733861
4	CTSD	0.38	0.29	1.93	1.13	0.78	0.00742603
4	RB1CC1	0.35	0.24	1.11	0.85	0.37	0.00754924
4	SH2D2A	0.42	0.31	1.72	1.28	0.42	0.0075648
4	MRPS21	0.27	0.18	0.85	0.59	0.53	0.00764233
4	ARF5	0.29	0.20	1.01	0.65	0.63	0.00768453
4	SELENOF	0.29	0.20	0.89	0.60	0.56	0.00822381
4	COPS9	0.37	0.26	1.16	0.88	0.41	0.00827777
4	DGUOK	0.26	0.17	0.86	0.55	0.64	0.00833127
4	RWDD1	0.44	0.33	1.61	1.24	0.38	0.00837968
4	TBCB	0.26	0.18	0.90	0.59	0.60	0.00838109

4	DCXR	0.34	0.25	1.22	0.86	0.50	0.00853132
4	SLAMF7	0.41	0.31	1.71	1.30	0.39	0.00870085
4	LIMS1	0.26	0.17	0.83	0.56	0.58	0.00890903
4	PLP2	0.41	0.31	1.60	1.09	0.56	0.00892853
4	BIRC3	0.70	0.64	14.74	10.57	0.48	0.00901912
4	LSM1	0.27	0.17	0.69	0.53	0.39	0.00902074
4	ADAM8	0.35	0.27	1.57	0.98	0.68	0.00913478
4	CANX	0.45	0.34	1.60	1.23	0.38	0.00927481
4	HERPUD2	0.33	0.23	1.16	0.85	0.45	0.00938434
4	CASP3	0.28	0.19	0.94	0.65	0.53	0.00962934
4	PRR13	0.36	0.27	1.34	0.90	0.58	0.00967358
4	AP2M1	0.29	0.20	0.90	0.62	0.54	0.01003678
4	BRWD1	0.27	0.20	1.21	0.62	0.96	0.01007472
4	SNRPD3	0.36	0.27	1.17	0.86	0.44	0.01023766
4	HES4	0.26	0.18	1.96	1.06	0.88	0.0104737
4	RAB6A	0.28	0.19	0.83	0.60	0.48	0.01055574
4	EVI2B	0.48	0.37	1.94	1.50	0.37	0.01060708
4	ACADVL	0.34	0.24	1.17	0.83	0.50	0.01100993
4	MBP	0.66	0.60	4.93	3.69	0.42	0.01104086
4	UBE2K	0.34	0.25	1.13	0.84	0.43	0.01127594
4	XRN1	0.39	0.30	1.54	1.06	0.54	0.01167414
4	MBD2	0.33	0.23	1.17	0.78	0.58	0.01236188
4	PREX1	0.46	0.35	2.04	1.42	0.53	0.0123733
4	DEGS1	0.27	0.19	0.78	0.58	0.44	0.01237859
4	ANAPC16	0.39	0.29	1.41	1.09	0.37	0.01283787
4	TRBC2	0.48	0.38	2.17	1.62	0.42	0.012914
4	ATP5F1D	0.51	0.42	2.24	1.62	0.47	0.01329637
4	EIF6	0.31	0.22	0.93	0.69	0.42	0.01347247
4	ERGIC1	0.33	0.23	1.03	0.80	0.37	0.01372851
4	SEC61B	0.59	0.52	2.86	2.22	0.36	0.01397658
4	TUFM	0.34	0.26	1.27	0.87	0.56	0.01422354
4	EIF2S2	0.42	0.31	1.62	1.25	0.37	0.01425459
4	ATP5PD	0.26	0.18	0.77	0.56	0.47	0.01472497
4	NDUFA4	0.51	0.43	2.21	1.66	0.41	0.01516973
4	SUZ12	0.34	0.24	1.15	0.82	0.49	0.01530448
4	CMTM3	0.27	0.19	0.85	0.55	0.65	0.01609143
4	BBLN	0.42	0.34	1.89	1.22	0.63	0.01696338
4	RPS19BP1	0.36	0.28	1.33	0.91	0.55	0.01718764
4	UBE2N	0.34	0.26	1.05	0.81	0.37	0.01747458
4	TCEA1	0.38	0.31	1.51	1.06	0.51	0.01759372
4	PHF12	0.27	0.19	0.84	0.61	0.45	0.01831399
4	PPIB	0.62	0.52	3.10	2.30	0.43	0.01891244
4	EMB	0.34	0.26	1.22	0.87	0.49	0.01939378
4	ATP5MJ	0.36	0.29	1.41	1.00	0.49	0.01945041
4	ZNHIT1	0.25	0.17	0.74	0.57	0.39	0.01967854
4	ATP2B4	0.29	0.21	1.08	0.78	0.46	0.01987596
4	ERH	0.34	0.25	1.12	0.82	0.45	0.02014978
4	USP10	0.27	0.19	0.74	0.55	0.43	0.02126953
4	S100A10	0.76	0.73	8.58	6.47	0.41	0.02144656
4	CEP350	0.38	0.28	1.39	1.04	0.43	0.02205117
4	PSMA1	0.36	0.28	1.48	0.95	0.63	0.02239738
4	GHITM	0.45	0.35	1.66	1.27	0.38	0.02293712
4	SMARCC1	0.29	0.21	0.97	0.68	0.51	0.02332595
4	FAM118A	0.29	0.21	1.14	0.69	0.72	0.02340784
4	IL16	0.25	0.18	0.98	0.63	0.63	0.02344701
4	TXNL4A	0.29	0.21	0.90	0.64	0.48	0.02378257
4	SIPA1	0.29	0.20	0.94	0.67	0.47	0.02401977
4	TRIP12	0.29	0.20	0.93	0.66	0.49	0.02429582
4	RPS6KA3	0.37	0.31	1.81	1.18	0.62	0.02460827

4	NDUFB9	0.29	0.21	0.87	0.66	0.40	0.02561679
4	SLK	0.27	0.20	0.92	0.67	0.45	0.02581951
4	NEMF	0.25	0.18	0.93	0.58	0.69	0.02641916
4	KRAS	0.44	0.36	1.82	1.30	0.49	0.02647177
4	SETD2	0.47	0.39	2.05	1.57	0.39	0.02684038
4	CELF1	0.34	0.27	1.25	0.91	0.46	0.02698476
4	CDC26	0.31	0.22	0.95	0.71	0.41	0.02753176
4	C4orf3	0.38	0.30	1.48	1.10	0.42	0.02776644
4	CD58	0.27	0.18	0.79	0.60	0.40	0.02874781
4	SKAP1	0.31	0.23	1.12	0.79	0.51	0.02895219
4	MTHFD2	0.30	0.22	0.95	0.73	0.38	0.02944947
4	TAF15	0.36	0.28	1.28	0.97	0.40	0.03144895
4	FERMT3	0.29	0.22	1.02	0.74	0.47	0.03200437
4	JPT1	0.33	0.26	1.26	0.90	0.48	0.0325232
4	FYB1	0.40	0.31	1.74	1.33	0.39	0.03434884
4	RAC1	0.56	0.48	2.57	1.96	0.39	0.03457494
4	SACM1L	0.41	0.34	1.57	1.20	0.39	0.03474303
4	ACTR2	0.52	0.45	2.36	1.78	0.40	0.03511883
4	NDUFA3	0.26	0.19	0.88	0.66	0.41	0.0357787
4	M6PR	0.33	0.25	1.10	0.82	0.42	0.03615685
4	PHB2	0.33	0.25	1.05	0.77	0.44	0.03702691
4	KCTD20	0.29	0.22	0.95	0.71	0.41	0.0374375
4	RAB5C	0.28	0.21	0.90	0.63	0.52	0.03861334
4	TRGC1	0.48	0.40	2.59	1.87	0.48	0.04071784
4	TANK	0.46	0.37	2.07	1.59	0.38	0.04145336
4	MED28	0.25	0.18	0.74	0.56	0.42	0.04184654
4	AHR	0.31	0.24	1.39	0.96	0.53	0.04258731
4	RAC2	0.62	0.57	3.80	2.96	0.36	0.04454672
4	NKTR	0.51	0.45	2.85	2.02	0.50	0.04519438
4	SLC4A7	0.27	0.20	0.99	0.69	0.52	0.04571954
4	CHIC2	0.31	0.25	1.15	0.76	0.59	0.04620798
4	DNAJC3	0.33	0.25	1.11	0.85	0.37	0.04744715
4	NDUFV2	0.26	0.20	0.81	0.61	0.42	0.04819614
4	PRKD3	0.27	0.20	0.84	0.65	0.37	0.04882629
4	CAPRIN1	0.31	0.24	0.98	0.75	0.39	0.04969352
5	IFIT1	0.52	0.01	3.16	0.02	7.04	2.61E-108
5	IFIT3	0.72	0.03	7.54	0.14	5.74	1.60E-76
5	MX2	0.60	0.05	5.31	0.16	5.10	2.79E-36
5	IFIT2	0.52	0.04	16.62	0.23	6.20	2.07E-35
5	IFI44	0.52	0.04	3.19	0.12	4.74	1.54E-31
5	MX1	0.60	0.06	4.20	0.20	4.41	4.78E-29
5	EPSTI1	0.64	0.09	3.82	0.30	3.65	3.00E-22
5	OAS1	0.28	0.02	1.61	0.05	4.89	7.91E-20
5	DDX58	0.36	0.03	1.45	0.10	3.82	1.38E-18
5	IFI6	0.52	0.08	4.56	0.27	4.06	2.92E-17
5	HERC5	0.52	0.08	6.25	0.25	4.65	8.81E-17
5	SAMD9L	0.48	0.08	2.16	0.30	2.86	1.10E-12
5	ISG15	0.92	0.49	32.57	3.40	3.26	1.37E-12
5	OAS2	0.32	0.05	1.17	0.14	3.05	1.02E-09
5	DDX60L	0.28	0.04	1.16	0.12	3.24	3.20E-09
5	PARP14	0.72	0.28	4.08	1.05	1.95	4.09E-09
5	IFI44L	0.28	0.04	2.09	0.17	3.59	1.15E-08
5	PLSCR1	0.44	0.11	1.96	0.33	2.58	1.75E-08
5	IFITM1	0.92	0.63	13.64	4.03	1.76	4.47E-08
5	RNF213	0.92	0.55	10.54	3.28	1.68	7.20E-08
5	LY6E	0.68	0.30	5.44	1.10	2.30	9.02E-08
5	ISG20	0.88	0.58	15.87	4.00	1.99	1.20E-07
5	XAF1	0.52	0.16	2.75	0.58	2.24	1.39E-07
5	PARP9	0.28	0.05	1.12	0.17	2.73	6.39E-07

5	DRAP1	0.76	0.40	6.10	1.56	1.97	9.25E-07
5	ANGEL2	0.28	0.05	0.89	0.18	2.29	9.94E-07
5	IRF1-AS1	0.52	0.18	2.67	0.66	2.01	1.15E-06
5	TRIM22	0.60	0.26	4.42	1.00	2.14	1.59E-06
5	EIF2AK2	0.44	0.15	3.05	0.50	2.61	3.09E-06
5	SP110	0.52	0.19	1.99	0.66	1.60	9.22E-06
5	KLF13	0.68	0.33	3.49	1.19	1.55	1.79E-05
5	HLA-E	1.00	0.93	22.27	11.84	0.91	2.60E-05
5	SP100	0.76	0.44	4.65	1.85	1.33	2.62E-05
5	CCL5	0.96	0.81	44.73	21.39	1.06	2.69E-05
5	NT5C3A	0.52	0.22	2.44	0.67	1.87	2.81E-05
5	SAMD9	0.52	0.23	3.01	0.80	1.91	4.29E-05
5	STAT2	0.32	0.09	1.57	0.28	2.48	5.94E-05
5	LAP3	0.36	0.11	1.39	0.33	2.09	6.45E-05
5	HLA-C	1.00	0.97	32.93	20.79	0.66	8.02E-05
5	PPM1K	0.44	0.18	2.71	0.58	2.23	0.00010072
5	SYTL3	0.88	0.61	10.39	3.93	1.40	0.00010098
5	PSME1	0.84	0.53	4.76	2.37	1.01	0.00010344
5	UBTF	0.36	0.12	1.37	0.39	1.82	0.00012609
5	ZBP1	0.28	0.08	1.08	0.27	2.01	0.0001409
5	PML	0.32	0.10	1.10	0.28	1.96	0.00015083
5	TYROBP	0.92	0.75	9.86	5.47	0.85	0.00019845
5	IFITM3	0.68	0.39	5.88	1.91	1.62	0.00020583
5	RBIS	0.56	0.26	2.02	0.88	1.21	0.00033347
5	LAG3	0.32	0.11	2.00	0.43	2.23	0.00035011
5	B2M	1.00	1.00	143.08	97.12	0.56	0.00038179
5	TRAPPC2L	0.28	0.09	0.94	0.25	1.92	0.00040828
5	TRANK1	0.28	0.09	1.17	0.27	2.12	0.00041419
5	BST2	0.64	0.37	3.26	1.50	1.11	0.0004723
5	XIST	0.48	0.19	2.38	1.22	0.97	0.00047774
5	ADAR	0.56	0.34	3.50	1.23	1.51	0.0005099
5	REEP5	0.56	0.28	2.01	0.92	1.12	0.000617
5	RARS2	0.32	0.11	1.21	0.36	1.74	0.00063238
5	DUSP12	0.28	0.09	0.80	0.25	1.65	0.00071275
5	NDUFB1	0.48	0.23	2.29	0.80	1.51	0.0008769
5	PRCC	0.28	0.10	1.08	0.26	2.06	0.00110184
5	LNPEP	0.60	0.32	2.49	1.25	1.00	0.00125106
5	CCL4	0.72	0.55	80.84	22.48	1.85	0.0013257
5	PLAAT4	0.60	0.35	2.83	1.40	1.02	0.00141666
5	HSH2D	0.48	0.24	2.59	0.96	1.44	0.00146336
5	SLFN5	0.52	0.28	3.46	1.32	1.39	0.00156463
5	CCL4L2	0.48	0.26	30.68	6.69	2.20	0.00203793
5	BTN2A1	0.40	0.18	1.53	0.57	1.43	0.00204582
5	CCL3L1	0.32	0.13	9.41	1.01	3.23	0.00207679
5	CWC22	0.28	0.10	0.99	0.32	1.64	0.00211218
5	PSMB9	0.60	0.36	4.23	1.42	1.57	0.00216195
5	ITGAE	0.36	0.16	1.80	0.57	1.65	0.00217013
5	MED29	0.28	0.10	0.91	0.33	1.48	0.00218253
5	STAT1	0.32	0.13	1.91	0.42	2.19	0.00248674
5	SLA	0.68	0.43	4.34	2.23	0.96	0.00248713
5	ADD3	0.40	0.18	1.41	0.61	1.20	0.00250908
5	GNG5	0.52	0.31	2.27	1.05	1.11	0.00284682
5	KLRC4	0.40	0.18	1.32	0.64	1.03	0.00293582
5	FIP1L1	0.36	0.16	1.28	0.47	1.44	0.0029733
5	STRN3	0.28	0.10	0.75	0.27	1.46	0.00297763
5	TXNDC15	0.28	0.11	1.16	0.33	1.81	0.00307725
5	TMSB10	1.00	0.96	42.34	21.74	0.96	0.00310679
5	ANXA5	0.44	0.22	2.18	0.82	1.41	0.00327448
5	AUTS2	0.44	0.22	2.18	0.83	1.39	0.00337196

5	IRF9	0.32	0.13	1.36	0.44	1.63	0.00342307
5	SAMD3	0.68	0.49	4.73	2.47	0.93	0.00344494
5	CCDC85B	0.60	0.37	3.05	1.47	1.05	0.00396113
5	MT2A	0.68	0.48	18.13	6.27	1.53	0.00401804
5	NKG7	0.96	0.92	33.29	23.17	0.52	0.00435568
5	TMEM123	0.56	0.37	3.74	1.53	1.29	0.00442176
5	MYL12A	0.88	0.68	6.59	4.93	0.42	0.0046333
5	IFITM2	1.00	0.85	16.20	10.75	0.59	0.00496123
5	ATP5MC2	0.72	0.57	4.66	2.65	0.82	0.00568215
5	IRF7	0.40	0.21	2.31	0.77	1.59	0.00591938
5	PMAIP1	0.60	0.36	6.58	2.24	1.55	0.00637191
5	CYBC1	0.28	0.11	1.00	0.39	1.34	0.00657246
5	SRSF1	0.36	0.16	0.96	0.47	1.01	0.00690884
5	MED15	0.36	0.17	1.09	0.51	1.10	0.00719508
5	RNMT	0.56	0.39	3.16	1.47	1.10	0.0074314
5	TXNIP	0.80	0.59	8.64	5.50	0.65	0.00817275
5	JAK3	0.28	0.12	1.23	0.36	1.78	0.00830034
5	GZMB	0.88	0.69	20.16	14.05	0.52	0.00830315
5	KLF9	0.28	0.11	1.00	0.40	1.32	0.00853567
5	SEPTIN7	0.88	0.74	6.95	4.66	0.58	0.00891406
5	C1orf21	0.48	0.28	2.08	1.05	0.99	0.0089335
5	CD47	0.48	0.28	2.43	1.00	1.29	0.00896098
5	SECISBP2L	0.28	0.12	0.87	0.36	1.28	0.00954935
5	GZMA	0.60	0.45	13.01	5.72	1.19	0.00958919
5	PPP2R5C	0.76	0.59	6.08	3.69	0.72	0.00967723
5	CCL3	0.52	0.34	34.95	7.37	2.24	0.01026725
5	SH2D2A	0.52	0.31	2.44	1.30	0.91	0.01063901
5	PARP8	0.56	0.38	3.25	1.51	1.10	0.01066448
5	GBP4	0.44	0.25	2.31	1.02	1.18	0.01067814
5	ME2	0.32	0.15	1.05	0.50	1.08	0.01071643
5	RPA2	0.28	0.12	0.97	0.37	1.38	0.0107429
5	GOLGA8B	0.36	0.19	1.43	0.62	1.22	0.0111486
5	ZNF652	0.28	0.12	1.10	0.41	1.42	0.01130212
5	EVL	0.52	0.33	3.42	1.44	1.25	0.01134767
5	GFOD1	0.40	0.22	1.97	0.76	1.38	0.01163881
5	IL18R1	0.36	0.19	2.22	0.73	1.60	0.01170651
5	TNFSF10	0.32	0.16	2.72	0.64	2.08	0.01191496
5	OASL	0.48	0.34	9.53	1.49	2.67	0.01232207
5	SHFL	0.36	0.20	1.50	0.64	1.24	0.01275841
5	CHMP5	0.28	0.13	1.49	0.39	1.95	0.0133866
5	PRKACB	0.52	0.27	1.59	1.05	0.60	0.01364711
5	PLCG2	0.32	0.16	1.73	0.54	1.69	0.01364985
5	GSTK1	0.36	0.21	1.74	0.67	1.38	0.01547689
5	MACROH2A	0.40	0.21	1.37	0.64	1.10	0.01582996
5	DHRS7	0.48	0.30	2.39	1.11	1.10	0.01626586
5	SNHG3	0.32	0.16	1.08	0.47	1.19	0.01640023
5	ZBTB38	0.36	0.19	1.56	0.72	1.12	0.01652054
5	ARL4C	0.84	0.68	8.56	5.95	0.52	0.01663925
5	AAK1	0.60	0.44	3.18	1.86	0.77	0.0168149
5	RER1	0.28	0.13	1.19	0.40	1.57	0.0169295
5	PSMB2	0.40	0.21	1.21	0.66	0.87	0.01698422
5	PSMC3	0.28	0.13	0.91	0.38	1.27	0.01700811
5	IFI16	0.72	0.62	6.53	3.76	0.79	0.01737506
5	GON4L	0.36	0.19	1.39	0.62	1.16	0.01832576
5	NEDD8	0.44	0.27	2.03	0.91	1.17	0.01890343
5	COX7C	0.88	0.76	6.30	4.57	0.46	0.01939348
5	RNF149	0.48	0.34	2.95	1.32	1.16	0.01964138
5	TRIM56	0.28	0.13	1.00	0.43	1.20	0.01987152
5	N4BP1	0.44	0.28	2.41	1.04	1.22	0.02035313

5	UBE2J1	0.28	0.14	1.80	0.40	2.17	0.02059144
5	TOB1	0.40	0.21	1.78	0.94	0.92	0.02125886
5	PDCD7	0.40	0.23	1.47	0.74	0.98	0.02264428
5	LINC01871	0.60	0.45	6.45	3.14	1.04	0.0232307
5	DAZAP2	0.48	0.34	2.42	1.18	1.04	0.02428588
5	PHF11	0.28	0.14	1.25	0.46	1.45	0.02430256
5	CEP78	0.32	0.17	1.47	0.66	1.16	0.02456967
5	PSMB8	0.60	0.44	2.88	1.71	0.75	0.02532449
5	F2R	0.28	0.14	1.71	0.49	1.79	0.02756173
5	PLCL2	0.28	0.13	0.72	0.38	0.92	0.02786248
5	CPNE1	0.48	0.32	2.10	1.17	0.85	0.02866445
5	SMDT1	0.48	0.30	1.75	0.98	0.84	0.0292149
5	UXT	0.64	0.46	2.71	1.74	0.63	0.02938044
5	ANKIB1	0.28	0.14	0.83	0.40	1.05	0.02965203
5	KLRK1	0.28	0.13	0.82	0.41	1.01	0.02971231
5	NFATC2	0.36	0.22	1.47	0.70	1.07	0.03347188
5	SH3BGRL3	0.84	0.76	9.32	5.93	0.65	0.03412786
5	ETS1	0.84	0.68	6.30	4.38	0.52	0.03415483
5	SP3	0.36	0.21	1.35	0.68	0.98	0.03439554
5	GBP5	0.44	0.29	2.52	1.41	0.83	0.03494941
5	SIPA1	0.36	0.21	1.20	0.69	0.81	0.03619195
5	ARMCX3	0.44	0.31	2.27	1.11	1.03	0.03685695
5	IK	0.56	0.40	2.84	1.55	0.87	0.03798962
5	BTN3A1	0.28	0.15	1.27	0.50	1.35	0.0385377
5	TMEM87A	0.28	0.14	0.91	0.43	1.09	0.03934942
5	GLUL	0.40	0.24	1.69	0.93	0.86	0.03938409
5	EIF3H	0.56	0.46	2.88	1.77	0.71	0.03967732
5	KLRC2	0.52	0.36	3.80	1.84	1.04	0.03984173
5	ARFGAP2	0.28	0.15	1.01	0.40	1.32	0.03993781
5	TBRG1	0.28	0.14	1.06	0.48	1.14	0.0420227
5	USP16	0.40	0.24	1.41	0.82	0.78	0.04233353
5	H2AZ2	0.76	0.57	3.87	2.69	0.53	0.04384626
5	S100A4	0.72	0.64	11.46	5.60	1.03	0.04450403
5	ABHD2	0.48	0.33	2.24	1.32	0.77	0.04811866
5	HADHA	0.40	0.26	1.64	0.82	1.00	0.04851733
5	ZBTB20	0.28	0.15	1.20	0.51	1.24	0.04906389
5	PHF3	0.52	0.36	2.12	1.34	0.67	0.04952757

Table S5 | Signature genes for gene set analysis		
Tumor stress signatures	NK cell activation signatures	GR target genes
DNAJB1	CCL3	FN1
DNAJB4	CCL4	KLF9
DNAJC1	CCL5	ANKRD1
DNAJC21	CXCL13	MT2A
DNAJC3	CXCL8	SNAI2
DNAJC7	GNLY	VIM
DNAJC8	GZMA	ID3
HIF1A	GZMB	POU5F1
HSP90AA1	GZMH	WNT5A
HSPA1A	GZMK	NFKBIA
HSPA1B	GZMM	DUSP1
HSPA4	PRF1	IRS2
HSPA5	LTB	RAMP1
HSPA6	NKG7	TXNIP
HSPA8	CST7	CD55
HSPA9	ZAP70	RGS2
HSPB1	TYROBP	GPR183
HSPD1	XCL1	FOSL2
HSPE1	XCL2	ANXA1
HSPH1	CSF1	ZFP36
GADD45A	EOMES	TNFAIP3
GADD45B	IL32	GADD45A
	FCGR3A	GADD45B
	FCER1G	BCL6
	IFNG	FKBP5
		TSC22D3

Table S6 | DEgenes of Dex treatment		
P <0.01, |Log2FC|>0.5		
gene name	meidum only vs Dex log2FC	medium only vs Dex p value
AREG	6.296576447	2.43834E-10
RN7SL832P	5.210629067	0.001879597
GFPT2	4.810620631	0.00063653
INSR	4.461524704	0.00167963
MGAM	4.455843882	0.002424043
FKBP5	3.941482143	3.13686E-35
PCSK2	3.598273837	0.005597773
FXYD7	3.566302999	0.004036219
DDIT4	3.193880578	3.436E-19
CXXC11	3.106175043	0.003419284
XYLT1	3.099577252	9.36173E-07
TSC22D3	3.053089618	9.65441E-14
RASGRP2	3.011115083	1.13665E-06
BCAT1	2.687097696	0.007938813
BNC2	2.642550028	0.000889196
DUSP1	2.500157601	0.000797199
ZBTB16	2.385264276	3.39505E-05
SMAP2	2.297516335	1.24983E-11
RGS1	2.253618047	4.33317E-05
LEF1	2.251291234	0.005572039
CXCR2	2.2484651	3.10227E-05
HPGD	2.024432499	0.000314993
OSBPL5	1.97528331	4.20811E-07
MMAB	1.9475172	0.00618255
HS3ST3B1	1.93584726	0.004774214
TLE1	1.93405308	0.000470141
RAP1GDS1	1.893067408	9.2697E-06
TXNIP	1.88384567	5.9727E-07
ADAM19	1.870519777	0.008521582
FAM129A	1.813705377	2.69383E-09
ZBED4	1.732642406	0.00094722
ANKRD9	1.720528185	0.009924738
KIR2DL3	1.715492261	0.003546276
PRDM1	1.695643438	2.12152E-06
SYTL3	1.693898383	3.74144E-06
WHSC1	1.690150068	0.004939435
TMEM173	1.684527313	0.003996836
LRRC8C	1.683081692	3.10618E-05
OGFRL1	1.670464485	0.006288269
TUBA4A	1.64776039	0.000507423
RNF130	1.647065318	0.009131806
SLC1A5	1.617881181	0.00027273
CD55	1.590484603	0.000247571
IL6ST	1.579284368	1.13692E-05
KIR2DL4	1.572710396	0.000486699
KLF9	1.571710066	0.000122539
SLC7A5	1.555160068	0.000187089
TMEM2	1.513898945	1.28525E-05
TNFAIP8	1.506216713	0.001380955
GPATCH8	1.439119777	8.50353E-06
CASC7	1.422664589	5.59182E-05
SPON2	1.416311037	0.002112859
ELOVL6	1.414324879	0.003698467
HERPUD1	1.41066479	0.001838478
TSPAN14	1.405532522	0.00180366
CCND3	1.369386878	5.61E-05

SH3BP5	1.358132128	0.001009764
SLC1A4	1.353165908	0.008840062
KLF2	1.349741132	0.003730295
PIK3IP1	1.342086358	0.003220173
KIF13B	1.330435772	0.000319171
CEBPB	1.323560219	0.008895788
AGO2	1.290440178	0.004582372
ETS1	1.285697295	2.57616E-06
HMGB2	1.27501757	0.000286778
PXN	1.270780613	0.001104541
DDI2	1.25330982	0.001248711
ZMYND8	1.243122734	0.0010387
FOXO1	1.237885434	0.000782011
NEAT1	1.233328536	0.007404282
EMB	1.231671028	0.000663748
CYTIP	1.23035984	1.01244E-05
IRF2BPL	1.229901384	0.006007675
FAIM3	1.21977315	0.006546005
GFOD1	1.211068089	0.001650821
CNOT6L	1.197856866	0.000343619
XPO6	1.184795806	0.005605266
SSH2	1.18038635	0.005768179
CD7	1.180004985	5.58645E-05
PARP8	1.175385705	0.000427296
CELF2	1.145350859	3.29262E-05
SRGN	1.138981445	4.77006E-05
IKZF1	1.119689424	0.001944128
FGFBP2	1.113291549	0.001060493
GALNT10	1.101496532	0.00215501
MCTP2	1.098876563	0.006155812
CXCR4	1.081581807	0.005321649
PTGER2	1.069660577	0.001676092
CHST11	1.04482673	0.002224091
SYNE1	1.04004579	0.008691204
ZFP36L2	1.035400828	0.001879153
TSPYL2	1.034668006	0.005914345
PPP2R5C	1.028717733	0.003271968
FOXN3	1.010675187	5.53071E-05
MAPK1	1.005633042	0.008814597
PIP4K2A	0.956506703	0.00045056
CEP78	0.946086124	0.009924402
MYH9	0.913258503	0.001558758
GLIPR1	0.909608942	0.006330655
HECA	0.819723272	0.008388118
CD53	0.797214393	0.001126431
CFLAR	0.742624867	0.003985771
CD164	-0.764441713	0.009796686
PLEK	-0.891867954	0.001381547
LSP1	-0.901097271	0.000940347
IL2RB	-0.931285195	0.001763753
MCL1	-0.996124628	0.001961784
ADAR	-1.03558784	0.003849879
TAPBP	-1.051899971	0.003905663
BCOR	-1.124108366	0.009827168
PPP1CA	-1.141290856	0.001209068
BZRAP1-AS1	-1.147602335	0.003159585
PLP2	-1.155539018	0.001775028
IL32	-1.163965872	0.003626731
PSMB9	-1.166722805	0.002444652

BIRC3	-1.18358659	0.00032543
SP110	-1.215377951	0.009605773
MRPS6	-1.221096397	0.005319444
TOX	-1.261816611	0.001108557
PHLDA1	-1.276841013	0.00647335
CD74	-1.29458757	9.52801E-08
PIM2	-1.301235885	0.002497341
SAMD9	-1.316268308	2.88717E-05
SBK1	-1.328271869	0.005418545
GBP5	-1.333214033	0.003395589
DENND3	-1.355907918	0.009604126
GBP4	-1.371438773	0.004218374
PARP14	-1.381249545	0.000654058
BTG2	-1.389742079	0.006502528
FAM105B	-1.396151802	0.001728706
IFITM9P	-1.398869877	0.008850878
IDH2	-1.408819543	0.00069379
C5orf56	-1.409503447	0.004302722
NAPA	-1.413055306	0.003035748
HLA-DRA	-1.420828544	0.005525605
NR3C1	-1.421784961	2.11847E-06
ORAI2	-1.423451041	0.006762657
NT5C3A	-1.430685032	0.001483797
TRIM22	-1.432369619	0.000372798
RARRES3	-1.448392546	0.000365351
APOL1	-1.500101941	0.005148456
WWC3	-1.535241989	0.000534554
BCL2L1	-1.550735626	0.000637087
CFL1P2	-1.564452934	0.00607379
SLC25A1	-1.57137887	0.00057295
DRAP1	-1.597391735	0.000628067
TMSB4XP6	-1.602714332	0.008364371
SCARB2	-1.615472663	0.008838042
LAP3	-1.618166504	0.005111353
ZCCHC2	-1.61902681	0.000370329
TMEM123	-1.623891871	2.77286E-05
APOL3	-1.653485831	0.000940508
FASLG	-1.687910952	0.000150744
PDIA3P	-1.705321357	0.009085529
GBP1P1	-1.711266824	0.005283224
TIGIT	-1.713374648	5.59994E-05
YIPF5	-1.732327511	0.000988891
PRMT7	-1.760937691	0.003448236
GBP1	-1.773038796	0.000967265
TRIM69	-1.792082746	0.000552177
SQLE	-1.808243274	0.001266225
PARP9	-1.823237402	0.000436809
C2orf68	-1.852354174	0.000827396
IFIT5	-1.858821678	0.000950583
XAF1	-1.863759579	1.62993E-06
PARP12	-1.864883553	0.0007554
MYD88	-1.866505762	0.001295288
DDX60	-1.868742034	1.5179E-07
PTMS	-1.874918474	0.00233377
SLAMF7	-1.889713435	0.000103635
BZRAP1	-1.897862215	0.000768743
TRIM21	-1.920347541	0.001854435
TRIM5	-1.924044661	0.008265123
EIF2AK2	-1.929873808	1.53539E-06

EHD4	-1.937759714	0.003098593
BST2	-1.941321365	0.000562132
IRF9	-1.943105679	0.004598973
DDX58	-1.953335628	1.00651E-05
CD226	-1.961179777	0.000604686
OAS2	-2.003509777	0.000218123
IL15RA	-2.012705968	0.002199703
DTX3L	-2.018565085	8.53993E-07
C15orf39	-2.018635193	0.001852639
TRAF1	-2.042651184	0.00026186
TREX1	-2.080236633	0.003458793
GLIPR2	-2.10359823	1.16464E-05
SAMD9L	-2.106934065	4.96556E-06
LAG3	-2.111930806	0.001067625
DDX60L	-2.135618	1.39941E-06
LDLR	-2.143091102	8.01723E-06
EPSTI1	-2.148570445	2.37236E-06
STAT1	-2.161079929	6.95894E-07
TRDC	-2.183193507	3.20996E-09
TNFSF10	-2.228946464	4.65682E-05
RAB37	-2.258466589	0.007607479
NCS1	-2.301353963	0.003080081
RASGEF1B	-2.306283076	0.007534367
OASL	-2.30871901	6.44055E-07
PARP10	-2.324360283	0.001547255
ADAP1	-2.345528575	0.003178393
XCL2	-2.373221382	0.006944423
BCL3	-2.39071305	6.61343E-06
KLF10	-2.399640035	0.006458489
MT2A	-2.401331232	1.67214E-05
HERC6	-2.408574889	2.46146E-05
HERC5	-2.433649349	0.008330399
ARHGAP31	-2.440230856	0.007816143
AC092580.4	-2.455428631	0.001905575
PLSCR1	-2.491392531	3.17409E-07
TCF7	-2.568608781	0.000185699
RTP4	-2.609586086	8.05361E-05
SPATS2L	-2.612854865	8.02755E-06
RELB	-2.630771271	2.9636E-05
RGS3	-2.630881369	4.8194E-06
DHX58	-2.632976927	0.001309923
TRAC	-2.704761607	0.000127384
IFIH1	-2.745083767	1.09483E-07
IFI44	-2.780335784	2.4789E-07
IRF7	-2.8070866	0.003173094
CD82	-2.817001559	2.30581E-08
USP49	-2.818025032	0.002303572
USP18	-2.830639001	0.004535263
ITM2C	-2.859268143	0.00275393
IFI6	-2.960413609	0.000171466
TNFSF14	-2.97007046	2.12737E-06
DNPH1	-3.030539445	0.001750866
LINC00299	-3.229579982	0.000897108
OAS1	-3.380277713	0.004505519
DENND5A	-3.422267882	0.005219281
CTC-512J14.7	-3.437789055	0.002728398
OAS3	-3.440471394	0.000942227
MX1	-3.470887319	0.000994382
IFI44L	-3.50980718	0.001528504

C1orf61	-3.548460774	0.005407629
CMPK2	-3.612874475	0.000209733
RSAD2	-3.627942927	5.8695E-05
TACO1	-3.751368843	0.000381598
ETV7	-3.903340752	0.005862492
IFIT2	-3.929901123	0.000105113
IFIT3	-4.102226081	0.004097288
IL1RN	-4.159617746	0.009471023
TTC26	-4.390952147	0.003377369
USP30-AS1	-4.420994538	0.006471802
IFIT1	-4.814855238	0.000142683
N4BP3	-4.828458234	0.001181234
TMEM78	-4.860267527	0.005092175
C1orf173	-6.649298267	4.06286E-06
SAMD7	-7.678600858	0.009504592

gene name	cytokine vs cytokine + Dex log2FC	cytokine vs cytokine + Dex p value
AREG	5.043843254	3.36739E-12
SPON2	4.848799958	7.26609E-24
THAP8	4.813189072	0.006493324
FFAR2	4.707601102	0.004903609
CXCR4	4.608253294	3.55679E-30
CH25H	3.773403098	0.005386792
TSC22D3	3.702040143	9.02038E-18
DUSP1	3.575363024	1.57628E-05
PIK3IP1	3.572319061	1.77702E-13
FKBP5	3.538799548	2.2646E-33
RASGRP2	2.967590491	1.47768E-05
KLF9	2.789895271	5.48421E-12
TLE1	2.747363259	0.000105104
TRABD2A	2.651203188	0.000511987
FSD1	2.638247004	0.00663685
KLF2	2.569273245	6.07337E-08
ELOVL6	2.455060394	6.29295E-09
VAV3	2.3097256	3.23814E-06
AGPAT4	2.301752404	1.66535E-05
YPEL1	2.230359712	0.000202741
CCNG2	2.110140163	0.004818035
HPGD	2.106494426	7.00425E-05
ENPP5	2.084805698	0.009358099
FCRL6	2.013421563	0.000318776
LPAR6	1.972106227	0.000128157
BNC2	1.964331499	0.009778585
TMEM173	1.91699234	0.000399592
ZFP36	1.875760039	2.26459E-05
ZFP36L2	1.834388855	2.99747E-08
TXNIP	1.830091369	8.77489E-07
GLUL	1.812156841	1.71338E-05
SMAP2	1.796626511	7.31404E-09
FAM115C	1.794554063	0.000616337
ABCB1	1.718080935	0.000125101
FAM102A	1.703086787	0.005059418
PLEKHG3	1.684609654	0.004337878
PARP8	1.651194758	5.09886E-07
FAIM3	1.648022122	0.001682198
RCBTB2	1.630075765	0.000781824
TMCC3	1.615789034	0.000607999

ABLIM1	1.612482096	0.003416037
MAPK1	1.583055597	1.95916E-05
AIM1	1.575119118	0.00083424
OGFRL1	1.566820022	0.008608822
BIN1	1.564970922	0.000462625
SLAMF6	1.535265091	0.000657431
OSBPL5	1.473839868	0.000100317
RGS1	1.45220068	0.002964234
DYRK2	1.446385179	0.001285707
CARD11	1.411138483	1.13551E-05
TRERF1	1.393726502	0.00095667
TSPAN5	1.386439518	0.006302498
SYNE1	1.33864478	0.000637909
PXN	1.326836223	0.000178389
SSH2	1.299691789	0.00164886
GLCCI1	1.225573494	0.003559196
TUBA4A	1.207663141	0.003258579
PARP4	1.197142061	0.000338119
FAM65B	1.189896168	0.00013488
SELPLG	1.16553563	0.003697083
CD55	1.146242741	0.001153328
BTG1	1.116915067	0.000804325
CBFB	1.104993401	8.20273E-05
TMEM2	1.099635418	0.00079159
NR1D2	1.050811873	0.002787404
KIAA1551	1.042499258	0.000547079
ST3GAL1	1.032815252	0.001970142
SMARCA2	1.027942786	0.003310796
FOXO1	1.020538508	0.003642932
EVI2B	1.008741026	0.003864572
POLR3GL	0.991723248	0.008760098
CASP8	0.989761136	0.005283292
CCDC69	0.986523867	0.006935976
TMEM66	0.985719425	0.008923417
DDIT4	0.984699198	0.00149051
SSH1	0.905089486	0.004251249
CD7	0.861770693	0.001666199
SRGN	0.780863699	0.004270824
MSN	0.694713635	0.001323824
SLC38A1	0.59183351	0.009950435
ID2	-0.738090434	0.00532609
RBPJ	-0.807975257	0.002049489
CCL5	-0.813858337	0.005261917
VASP	-0.830544635	0.009460254
SH2D2A	-0.856659669	0.002694664
IL2RB	-0.859259968	0.00183395
PRKX	-0.861891676	0.00355197
CMTM6	-0.908983075	0.00535513
PLEK	-0.910009723	0.000548299
CD44	-0.945516299	0.006238405
TMSB4XP4	-0.956683124	0.000392379
TGFBR3	-0.990688091	0.003387914
ZFP36L1	-1.080145651	0.001873318
GADD45B	-1.10083103	0.000650396
TRGC1	-1.156981249	0.000893749
REL	-1.159243573	0.001370546
TUBBP1	-1.160918171	0.002663144
PRR5L	-1.173545761	0.0022813
STAT5A	-1.206841254	0.003312977

BHLHE40	-1.215610187	2.53085E-05
TRGC2	-1.257616668	0.001043913
MAP3K8	-1.26717728	0.000341644
PDE4B	-1.269866385	0.006678497
BATF	-1.289809275	0.007509247
IER3	-1.308853624	0.009123558
CREM	-1.310207068	0.001149936
VIM	-1.337127623	7.5426E-05
PTMS	-1.339149087	0.009609039
TNFSF14	-1.349395791	0.00942813
IRF2BP2	-1.354792299	4.58531E-05
FRMD4B	-1.359049377	0.006682612
TIGIT	-1.404863917	0.000498991
BCL6	-1.406273859	0.000549132
PDE4A	-1.412096383	0.000460493
TRDC	-1.412497115	5.74336E-05
NFAT5	-1.418190046	0.001214154
TNFAIP8	-1.422097892	0.000800025
FOXP4	-1.446555187	0.001337341
ITGA1	-1.462599114	0.005170582
KDM6B	-1.529372143	0.001415732
SOCS3	-1.628302447	0.006005922
JUNB	-1.633450476	6.03898E-07
LTB	-1.656557958	0.001985663
LGALS1	-1.656679886	0.001015806
TNFSF10	-1.664170271	0.000755773
NCS1	-1.751781511	0.00144436
TRAF1	-1.760168533	0.000261394
TRAC	-1.760771818	0.007718554
TNFRSF18	-1.818785405	0.000464477
SATB1	-1.861145419	1.58267E-07
TPM3P8	-1.943909229	0.006881615
FEZ1	-2.045040708	0.000145393
RGS16	-2.065659343	9.02523E-10
TNF	-2.156979893	2.27488E-06
CD83	-2.217384016	2.06317E-08
CD274	-2.226145361	0.000120979
MFSD2A	-2.311035708	0.004687912
CCR7	-2.324021166	0.001689433
BCL2A1	-2.341071025	0.00048591
CD82	-2.381449736	5.2578E-08
TMEM217	-2.440233784	0.009525414
EGR2	-2.48394869	0.000126826
RGCC	-2.488755814	0.002640976
CSF1	-2.493895707	2.73335E-05
ADAM19	-2.59074428	1.51552E-08
DUSP4	-2.66846923	1.51835E-09
GRAMD1B	-2.681751436	0.000506191
TNFRSF9	-2.975052357	1.29189E-06
BATF3	-3.009516493	0.000441672
HBEGF	-3.141112394	0.000234841
LTA	-3.275528828	3.00389E-16
TNFRSF4	-3.328205338	5.19627E-07
XCL1	-3.431044241	1.89323E-05
XCL2	-3.586179936	1.32689E-05
IL1RN	-3.895222537	0.000501944
GPR183	-5.519477275	8.88639E-08

Table S7 | Reagent and resource		
**Reagent or resource**	**Source**	**Identifier**
**Antibodies**		
Anti-Human CD3	Biolegend	Cat# 317306
Anti-Human CD4	Biolegend	Cat# 317408
Anti-Human TCRα/β	Biolegend	Cat# 306706
Anti-Human TCRγ/δ	Biolegend	Cat# 331208
Anti-Human CD19	Biolegend	Cat# 302206
Anti-Human CD20	Biolegend	Cat# 302304
Anti-Human CD22	Biolegend	Cat# 363508
Anti-Human CD14	Biolegend	Cat# 325604
Anti-Human CD34	ebioscience	Cat# 11-0349-42
Anti-Human FcεR1α	ebioscience	Cat# 11-5899-42
Anti-Human CD1a	Biolegend	Cat# 300104
Anti-Human CD11c	Biolegend	Cat# 301604
Anti-Human CD123	Biolegend	Cat# 306014
Anti-Human BDCA2	Biolegend	Cat# 354208
Anti-Human TBX21	ebioscience	Cat# 25-5825-82
Anti-Human IFN-γ	ebioscience	Cat# 17-7319-82
Anti-Human IFN-γ	ebioscience	Cat# 45-7319-42
Anti-Human TNF-α	ebioscience	Cat# 45-7349-42
Anti-Human AREG	ebioscience	Cat# 17-5370-42
Anti-Human CD45	ebioscience	Cat# 56-9459-42
Anti-Human CD56	Biolegend	Cat# 318306
Anti-Human CD56	BD	Cat# 564057
Anti-Human CD107a	ebioscience	Cat# 12-1079-42
Anti-Human Granzyme A	Biolegend	Cat# 507219
Anti-Human Granzyme B	Biolegend	Cat# 396413
Anti-Human NKG2A	Biolegend	Cat# 375111
Anti-Human NKG2D	Biolegend	Cat# 339513
Anti-Human CD158	Biolegend	Cat# 320831
Anti-Human AREG	R&D	Cat# AF262-SP
Anti-Human IFN-γ	eBioscience	Cat# 16-7318-85
**Biological Samples**		
PBMCs	Jiangsu province blood center	https://www.jsblood.com.cn/
A375	ATCC	Cat# CRL-1619
A431	ATCC	Cat# CRL-1555
A2058	ATCC	Cat# CRL-3601
HepG2	NA	Provided by Dr. Ningning Liu, Institute of Microbiology, Chinese Academy of Sciences
Huh7	NA	Provided by Dr. Ningning Liu, Institute of Microbiology, Chinese Academy of Sciences
HCT116	NA	Provided by Dr. Ningning Liu, Institute of Microbiology, Chinese Academy of Sciences
**Chemicals, Peptides, and Recombinant Proteins**		
IL-2	Novoprotein	Cat# C013
IL-12	Novoprotein	Cat# CI58
IL-15	Novoprotein	Cat# C016
IL-18	Novoprotein	Cat# CH29
IL-21	Novoprotein	Cat# CC45
4-1BBL	Novoprotein	Cat# CH04
MICA	Novoprotein	Cat# C489
Transferrin	Novoprotein	Cat# CJ41
IGF-I	Novoprotein	Cat# C032
IGF-II	Novoprotein	Cat# CF61

Insulin	Solarbio	Cat# I8830
PMA	Beyotime	Cat# S1819
Ionomycin	Beyotime	Cat# S1672
Mifepristone	MedChemExpress	Cat# HY-13683
Dexamethasone Sodium Phosphate Injection	Shiyao YINHU Pharmaceutical Co., Ltd.	Lot# 1022122362
Methylprednisolone Sodium Succinate	Sinopharm Ronshyn Pharmaceutical Co., Ltd.	LOT# 22101707
Compound Betamethasone Injection	Schering-Plough Labo N.V.	LOT# 00001473561
PF-04418948 (PTGER2 inhibitor)	MCE	Cat# HY-18966
L-161982 (PTGER4 inhibitor)	MCE	Cat# HY-108559
SQ22536 (adenylate cyclase inhibitor)	MCE	Cat# HY-100396
666-15 (CREB inhibitor)	MCE	Cat# HY-101120
Cell stimulation cocktail	ebioscience	Cat# 00-4970-03
Protein transport inhibitor	ebioscience	Cat# 00-4980-03
TRIzol reagent	Invitrogen	Cat# 15596026
RNAClean XP	Beckman Coulter	Cat# A63987
Alt-R S.p. Cas9 Nuclease V3	Integrated DNA Technologies	Cat# 1081059
ExoSAP-IT	Affymetrix	Cat# 78200
SuperScript™ III Reverse Transcriptase	Invitrogen	Cat# 18080-093
Fixable Viability Dye efluor 780	Invitrogen	Cat# 65-0865-18
Corticosterone	MCE	Cat# HY-B1618
Dydrogesterone	MCE	Cat# HY-B0257A
Estrone	MCE	Cat# HY-B0234
Progesterone	MCE	Cat# HY-N0437
Salmeterol	MCE	Cat# HY-14302
Testosterone	Sigma	Cat# T-037
Triiodothyronine	Beyotime	Cat# ST1677
Calcein AM	Beyotime	Cat# C2012
Recombinant Human AREG	Novoprotein	Cat# CG04
Recombinant Human IFN gamma	Novoprotein	Cat# CI57
Gefitinib	MCE	Cat#HY-50895
SNX-5422	MCE	Cat#HY-10213
Critical Commercial Assays		
Universal DNA purification kit	Tiangen	Cat# DP214-02
Blood genomic DNA isolation kit	Karroten	Cat# K2302
RNA fragmentation reagents	Invitrogen	AM8740
P3 Primary Cell 4D-NucleofectorTM X Kit S	Lonza	Cat# V4XP-3032
EasySep Human NK Cell Isolation Kit	STEMCELL	Cat# 17955
Hyperactive ATAC-Seq Library Prep Kit for Illumina	Vazyme	Cat# TD711
NK MACS Medium human	MACS	Cat# 130-114-429
Single Cell 3’ Reagent Kits v3.1	10 X Genomics	Cat# PN-1000121
NEBNext Ultra II Non directional Second Strand Synthesis Module	NEB	Cat# E6111L
HiScribe T7 High Yield RNA Synthesis Kit	NEB	Cat# E2040S
**Software and Algorithms**		
FlowJo	FlowJo, LLC	https://www.flowjo.com/
GraphPad Prism	GraphPad Software, Inc	https://www.graphpad.com/scientific-software/prism/

Cell Ranger	10x Genomics	https://support.10xgenomics.com/single-cell-gene-expression/software/pipelines/latest/what-is-cell-ranger
SAMtools	Li H. et al.	http://www.htslib.org
R Statistical Computing Software	The R Foundation	https://www.r-project.org/
JASPAR	JASPAR	http://jaspar.genereg.net
DESeq2	Love M. et al.	https://bioconductor.org/packages/release/bioc/html/DESeq2.htmll
Seurat 3.0	Stuart T. et al.	http://satijalab.org/seurat/
Bowtie2	Langmead B. et al.	https://bowtie-bio.sourceforge.net/bowtie2/index.shtml
CEL-Seq2	Hashimshony T. et al.	https://github.com/yanailab/celseq2
MACS2	Zhang Y. et al.	https://github.com/macs3-project/MACS/wiki/Install-macs2
Trimmomatic	Bolger A.et al.	https://github.com/usadellab/Trimmomatic
IGVTools	Robinson J. et al.	https://software.broadinstitute.org/software/igv/download
